# Functional understanding of secondary cell wall cellulose synthases in *Populus* *trichocarpa* via the Cas9/gRNA‐induced gene knockouts

**DOI:** 10.1111/nph.17338

**Published:** 2021-06-10

**Authors:** Wenjing Xu, Hao Cheng, Siran Zhu, Jiyao Cheng, Huanhuan Ji, Baocai Zhang, Shenquan Cao, Chong Wang, Guimin Tong, Cheng Zhen, Liqiang Mu, Yihua Zhou, Yuxiang Cheng

**Affiliations:** ^1^ State Key Laboratory of Tree Genetics and Breeding Northeast Forestry University Harbin 150040 China; ^2^ School of Forestry Northeast Forestry University Harbin 150040 China; ^3^ Institute of Genetics and Developmental Biology Chinese Academy of Sciences Beijing 100101 China

**Keywords:** Cas9, cellulose synthase (CesA), gelatinous layer (G‐layer), gene knockout, gRNA, phloem fibre, *Populus* *trichocarpa*, secondary cell wall (SCW), tension wood

## Abstract

- Plant cellulose is synthesized by a large plasma membrane‐localized cellulose synthase (CesA) complex. However, an overall functional determination of secondary cell wall (SCW) CesAs is still lacking in trees, especially one based on gene knockouts.
- Here, the Cas9/gRNA‐induced knockouts of *PtrCesA4*, *7A*, *7B*, *8A* and *8B* genes were produced in *Populus trichocarpa*. Based on anatomical, immunohistochemical and wood composition evidence, we gained a comprehensive understanding of five SCW *PtrCesAs* at the genetic level.
- Complete loss of *PtrCesA4*, *7A/B* or *8A/B* led to similar morphological abnormalities, indicating similar and nonredundant genetic functions. The absence of the gelatinous (G) layer, one‐layer‐walled fibres and a 90% decrease in cellulose in these mutant woods revealed that the three classes of SCW PtrCesAs are essential for multilayered SCW structure and wood G‐fibre. In addition, the mutant primary and secondary phloem fibres lost the *n*(G + L)‐ and G‐layers and retained the thicker S‐layers (L, lignified; S, secondary). Together with polysaccharide immunolocalization data, these findings suggest differences in the role of SCW *PtrCesAs*‐synthesized cellulose in wood and phloem fibre wall structures.
- Overall, this functional understanding of the SCW *PtrCesAs* provides further insights into the impact of lacking cellulose biosynthesis on growth, SCW, wood G‐fibre and phloem fibre wall structures in the tree.

## Introduction

Plant cellulose microfibrils (CMFs), which are fibrillar crystalline aggregates of β‐1,4‐glucans, are synthesized by the large plasma membrane (PM)‐localized cellulose synthase (CesA) complex (CSC) (Somerville, 2006; McFarlane *et al*., 2014). In earlier studies, plant CSCs have been visualized as rosette structures by freeze‐fracture studies and each rosette has six particles, each of which is postulated to contain six CesA subunits (Herth, 1983; Kimura *et al*., 1999; Doblin *et␣al*., 2002). The CSC is supposed to contain 36 CesAs, which might synthesize 36‐chain CMFs. Other studies have suggested 24‐chain CMFs in celery collenchyma and spruce wood (Fernandes *et␣al*., 2011; Thomas *et␣al*., 2013). The data from Nixon *et␣al*. (2016) and Purushotham *et␣al*., (2020) support a rosette CSC with 18 CesAs that mediate the synthesis of a fundamental microfibril composed of 18 glucan chains. For higher plant CSCs, the identity and arrangement of CSC CesAs in diverse plant species, especially in trees, remain to be widely investigated at the genetic level.

Higher plants have evolved a gene family including multiple CesAs that are classified into primary cell wall (PCW) and secondary cell wall (SCW) CesA groups. In *Arabidopsis*, CesA1, 3 and 6 (‐like) synthesize PCW cellulose, whereas CesA4, 7 and 8 synthesize SCW cellulose (Taylor *et␣al*., 2003; Desprez *et␣al*., 2007; Persson *et␣al*., 2007). Mutation of any of the three SCW AtCesAs causes the same irregular xylem (irx) phenotype with collapsed tracheary elements, demonstrating the nonredundant function of each SCW AtCesA (Taylor *et␣al*., 2004). In cellulose‐rich trees, genetic studies leading to a functional understanding of the SCW CesAs, especially studies using the complete loss‐of‐function, are limited. *Populus trichocarpa*, as a model tree species with a well‐annotated genome (Tuskan *et␣al*., 2006; Jansson & Douglas, 2007), has 17 *CesA* genes and multiple expression data show the involvement of several *CesA* genes in SCW synthesis (Kalluri & Joshi, 2004; Djerbi *et␣al*., 2005; Suzuki *et␣al*., 2006; Dharmawardhana *et␣al*., 2010). A phylogeny‐based CesA nomenclature presents five *Populus* CesAs (PtiCesA4, 7A, 7B, 8A and 8B) into the SCW CesA group (Kumar *et␣al*., 2009). Proteomic data have implied that CesA4, 7A/B and 8A/B, and CesA1A/B, 3C/D and 6E/F assemble into two types of *Populus* CSCs, which might contribute simultaneously to cellulose biosynthesis in wood SCWs (Song *et␣al*., 2010; Xi *et␣al*., 2017). Overexpression of an aspen mutant CesA8 lacking one of the N‐terminal methionines unexpectedly resulted in a silencing of the transgene and revealed the role of CesA8 in vertical tree growth (Joshi *et␣al*., 2011; Liu *et␣al*., 2012). Most recently, SCW CesA stoichiometry in developing aspen xylem was suggested by quantitative proteomics to be 3 : 2 : 1 (Zhang *et␣al*., 2018). To gain insight into the roles of SCW CesAs and their CSC models in trees, it is essential to knock out each of the SCW CesAs.

The wood cell wall is a multilayered structure that includes, from outside to inside, the middle lamella (ML), PCW and SCW. These wall layers have differences in CMF organization and ratios of cellulose to wall matrix components (Plomion *et␣al*., 2001; Barnett & Bonham, 2004). The SCWs of wood cells comprise three layers (S1–S3), of which S2 is the most important for mechanical support and accounts for 75–85% of the SCW thickness. In response to certain environmental cues (e.g. wind and slope), the stems and branches of trees perceive gravity to determine their orientation and, in response, produce reaction wood to reinforce their position (Du & Yamamoto, 2007; Felten & Sundberg, 2013; Groover, 2016). In angiosperms, the upper sides of these stems and branches create tension wood (TW) to pull them upward. At the ultrastructural level, the TW of poplar stems has shown specific anatomical changes, such as a decrease in vessel density and an increase in fibre length (Jourez *et␣al*., 2001). In addition, the fibres of the TW develop a thick gelatinous (G) layer in their walls, and so are called G‐fibres, where the G‐layer can replace the S3 or S2 + S3 layer (Wardrop & Dadswell, 1955; Nishikubo *et␣al*., 2007; Kwon, 2008). The chemical composition of the G‐fibres includes high cellulose content and low lignin, and the polysaccharide composition of the wall matrix in the G‐layers differs from that of the PCWs and SCWs (Mellerowicz & Gorshkova, 2012; Fagerstedt *et␣al*., 2014) and can produce high glucose yields for biofuel production (Brereton *et␣al*., 2011, 2012). In phloem fibres, the cell wall structures are classified into three types: S1 + S2, S1 + S2 + G and S1 + S2 + *n*(G + L), where *n* indicates the number of repetitions of the G‐layer and the thin lignified layer (L) (Nanko, 1979; Nakagawa *et␣al*., 2012, 2014). Considering that the G‐layers are developed after the PCWs and SCWs and have a distinct composition, architecture and physical properties (Yamamoto *et␣al*., 2010; Mellerowicz & Gorshkova, 2012), they should be considered as tertiary cell walls (TCWs; Gorshkova *et␣al*., 2018). However, little is known about the roles of SCW CesAs in the TCWs of wood and phloem fibres of trees at the genetic level.

Knockout mutants are a crucial genetic tool for uncovering gene functions and biological mechanisms. Genome editing techniques are promising for the production of tree gene knockouts, and Cas9/gRNA genome editing has been demonstrated in poplar, apple and grape to generate null mutations in the first generation (Fan *et␣al*., 2015; Zhou *et␣al*., 2015; Osakabe *et␣al*., 2018). In the present study, we produced multiple knockouts of *PtrCesA4*, *7A*, *7B*, *8A* and *8B* genes in *P*.* trichocarpa* through Cas9/gRNA‐targeted mutagenesis. A comprehensive functional analysis revealed that complete loss of *PtrCesA4*, *7A/B* or *8A/B* led to similar morphological abnormalities, reduced wood cellulose content by 90% and suggested similar and differential roles in wood and phloem fibre wall structures.

## Materials and Methods

### Plant material and growth conditions

The *Populus* *trichocarpa* genotype Nisqually‐1 was used in this study. Sterile plantlets were propagated for genetic transformation in a growth chamber (25–27°C, 16 h : 8 h, light : dark photoperiod) with a light intensity of 60–80 μmol m^−2^ s^−1^. The asexual propagation of the transgenic plants was performed using three methods: apical bud cloning, lateral bud cloning and shoot regeneration. Apical and lateral buds were cut from young trees and water‐cultivated to rooting for three weeks, and shoot regeneration propagation was performed as described previously (Li *et␣al*., 2017). The plantlets generated were grown for phenotypic analysis in a glasshouse (25–28°C, 16 h : 8 h, light : dark photoperiod) with a light intensity of *c*. 250 μmol m^−2^ s^−1^. In addition, the wild‐type (WT), and *ptrcesa4*, *7ab*, *8a*, *8b* and *8ab* mutants were fixed to sticks to grow straight for 4 months in the glasshouse and TW was induced by inclining the stems to a 45° angle from the vertical direction for 10 d.

### gRNA design and vector construction

We used the CRISPRdirect (http://crispr.dbcls.jp/; Naito *et␣al*., 2015) to acquire efficient gRNA target sites for genes of interest. In addition, the gRNAs were selected as close to the 5′‐ends of the gene coding sequences (CDS) as possible so that the induced frameshift mutations at the target sites would conveniently result in loss‐of‐function alleles. To ensure targeting specificity, the target sequence of each gRNA selected was used in a BlastN search against the *P*.* trichocarpa* genome and did not contain single nucleotide polymorphisms (SNPs) or small insertion/deletion polymorphisms (indels).

For cloning of the Cas9/gRNA constructs, we used the method described previously, and pCBC‐DT1T2 and pHSE401 plasmids kindly were provided by Qi‐Jun Chen from China Agricultural University (Xing *et␣al*., 2014). The PCR fragment was amplified using pCBC‐DT1T2 as a template, and the purified PCR fragment and pHSE401 plasmid were set up for the Golden Gate reaction using *Bsa*I and T_4_ ligase. The obtained pHSE401‐2gRNA vector, after sequencing, was transferred into *Agrobacterium* strain GV3101.

### Genetic transformation of Nisqually‐1

*Agrobacterium*‐mediated transformation of Nisqually‐1 was performed according to our protocol (Li *et␣al*., 2017), and hygromycin selection concentrations for transformants were determined. *Agrobacterium* carrying the pHSE401‐2gRNA binary vector was incubated to an OD_600_ of 0.5–0.6, and the pellet after centrifugation was resuspended for transformation. Stem explants from 1‐month‐old sterile plantlets were excised to produce fragments of 1.0–1.2 cm in length and infected for 25 min. The infected explants were co‐cultivated for 2 d, and shoot transformants were induced for 25–30 and 10–15 d in selection media supplemented with 10 and 5 mg l^−1^ hygromycin, respectively. The hygromycin‐resistant shoots were transferred to rooting medium with 5 mg l^−1^ hygromycin for 10–15 d, and the rooting shoots were objective transformants.

### Identification of mutations and verification of genotype stability

Genomic DNA was extracted from the leaves of WT plants and transformants using a Plant Genomic DNA Extraction Kit (BioTeke Corp., Wuxi, China). The presence of the transgene was determined in the transformants by genomic DNA PCR with *zCas* (*Zea mays*‐codon optimized *Cas9*) and *Hyg* primers. After the growth of the transgenic plants in the glasshouse for 30 d, their genomic DNA was used for PCR amplification with gene‐specific primers spanning target sites; all primers are shown in Table S1. PCR‐amplified fragments were cloned using the pMD18‐T vector (TaKaRa, Beijing, China), and 25 positive clones for each amplicon were sequenced to identify editing at the target site. To observe the inheritance of the Cas9/gRNA‐induced mutations, the progeny of the *ptrcesa* mutants were propagated asexually using three methods: apical bud cloning, axillary bud cloning and shoot regeneration. Each target locus was amplified, and the PCR amplicon was sequenced as described above.

### Antibody production, protein isolation, and western blot

Two specific peptides for each class of the SCW PtrCesAs (PtrCesA4, 7A/B and 8A/B) were synthesized by Hangzhou HuaBio (http://www.huabio.com) as antigens to raise antibodies in rabbits. Three groups of peptides KDELRPPTRQSATLC/IKHHDHDESNQKNVC, EHKPLKNLDGQVC/CGRGHDDEENSQFP and STMASHLNNSQDVC/CPAQDPAEVYKDAKR represent PtrCesA4, PtrCesA7A/B and PtrCesA8A/B, respectively. Two rabbits were injected for each peptide. The immunoglobulin fraction was purified from rabbit antiserum with protein A‐agarose beads. The obtained antibody each was applied to Western blot with *Populus* xylem protein as an antigen to examine antibody specificity.

For Western blot analysis, the xylem tissues were ground into powder in liquid nitrogen and homogenized with ultrasonic shaking in extraction buffer (100 mM Tris‐HCl, pH 8.0; 1% SDS) for 30 min at 4°C. The homogenate was boiled in a water bath for 10 min and isolated by centrifugation at 14 000 ***g*** for 20 min. Protein extracts were loaded onto an 8% SDS‐PAGE gel and transferred to a PVDF membrane. Each membrane was incubated in blocking solution containing anti‐PtrCesA4 antibody (1 : 1500), anti‐PtrCesA7A/B antibody (1:500), or anti‐PtrCesA8A/B antibody (1 : 500) for 1 h, and then washed five times in wash buffer (1 × TBS with 0.1% Tween 20). Subsequently, the membrane was incubated in blocking solution containing HRP‐conjugated anti‐rabbit secondary antibody (1:5000, ab205718; Abcam, Cambridge, UK). The signals were captured using ECL Western Blotting Substrate (Pierce Biotechnology 32106; Thermo Fisher Scientific, Waltham, MA, USA) by exposure to X‐ray films. The ACTIN was detected as a loading control using an anti‐Actin antibody (ab197345; Abcam).

### Immunolocalization of cell wall polysaccharides

Samples were fixed in FAA buffer (50% ethanol, 5% acetic acid and 3.7% formaldehyde) and embedded in paraffin. Transverse sections of 8 μm thickness were cut with a sliding microtome (HM340E; MICROM International GmbH, Walldorf, Germany). Immunolocalization of wall polysaccharides (xylan, β‐(1 → 4)‐galactan, mannan and crystalline cellulose) was conducted in TW and phloem fibres of WTs and mutants. The sections were incubated with carbohydrate‐specific antibody (LM10/5/21, Plant probes, https://plantcellwalls.leeds.ac.uk/plantprobes/) or CBM3a‐6 × His protein (Plant probes) in the dilution buffer (1 : 100). Signals were detected with Alexa Fluor 633 goat anti‐rat IgG (A21094, Invitrogen) in dilution buffer (1 : 100) or anti‐His tag (1 : 500, ab18184, Abcam) as secondary antibodies and then incubated with Alexa Fluor 633 goat anti‐rat IgG (1 : 100). Sections were observed under a Zeiss LSM800 confocal laser‐scanning microscope.

### Microscopy analyses

For light microscopy, stem, petiole and root samples were fixed, embedded in paraffin, sectioned and stained with toluidine blue and phloroglucinol‐HCl, as described previously (Liu *et␣al*., 2015). For scanning electron microscopy (SEM), free‐hand cross‐sections of fresh stem segment samples were coated with gold (Au), transferred to an SEM (S‐4800; Hitachi, Tokyo, Japan) chamber and imaged to analyse the wood wall thickness and cell shapes of xylem fibres, vessels, rays, pith and phloem fibres. For transmission electron microscopy (TEM) observation as described previously (Zhou *et␣al*., 2009), the stem xylem and bark samples of WT and mutants were cut into 0.5‐mm pieces and fixed. After postfixing in 1% OsO_4_, the samples were embedded in Epon 812 resin and 80‐nm‐thick ultrathin sections were prepared, which were stained in uranium acetate followed by lead citrate. Section‐mounted grids were observed using a TEM (CM120; Phillips, Eindhoven, the Netherlands) at an acceleration voltage of 80 kV and imaged for analyzing the wood wall thickness and structure.

### Wood composition assay

The basal stems from 6‐month‐old mutant and WT trees were peeled, air‐dried and ball‐milled into the fine powder. After the power was washed with 70% aqueous ethanol, chloroform/methanol (1 : 1 v/v) solution and acetone successively, the obtained insoluble residues were prepared into cell wall materials, as previously described (Foster *et␣al*., 2010b), for crystalline cellulose and lignin content assays. The lignin content was determined using two methods: the acetyl bromide spectrophotometric method (Foster *et␣al*., 2010a) and Klason lignin and acid‐soluble lignin for the total lignin content, as described previously (Nakano & Meshitsuka, 1992). The crystalline cellulose content was measured according to a method described previously (Foster *et␣al*., 2010b). The hemicellulose content was determined by the GC‐MS method, as described previously (Zhang *et␣al*., 2017).

### Statistical analysis

Data were analyzed by ANOVA in Spss (17.0). Student’s *t*‐test was used to determine statistical significance between the mutant and WT samples. Values are the means ± SD, and asterisks indicate statistical significance at different levels (*, *P* < 0.05; **, *P* < 0.01; ***, *P* < 0.001).

### Accession numbers

The sequences used in this study are available in Phytozome (v.12.0) under the following accessions: *PtrCesA4* (Potri.002G257900), *PtrCesA7A* (Potri.006G181900), *PtrCesA7B* (Potri.018G103900), *PtrCesA8A* (Potri.011G069600), and *PtrCesA8B* (Potri.004G059600).

## Results

### Cas9/gRNA‐induced knockout of SCW *PtrCesAs* in *P*.* trichocarpa*

In order to produce gene knockouts in *P*.* trichocarpa*, we developed the Cas9/gRNA‐targeted mutagenesis system in genotype Nisqually‐1 (Supporting information, Fig. S1). Owing to an observable albino phenotype of *chli* mutants (Huang & Li, 2009), *PtrCHLI* genes were tested for the gene editing mutagenesis in Nisqually‐1. For single*‐*gene editing, 11 biallelic/homozygous (61.1%) and five monoallelic mutation lines showed albino/pale‐green phenotypes, indicating knockout/knockdown of *PtrCHLI1* (Fig. S2a–d; Table S2). The Cas9/gRNA‐induced editing efficiency was further tested in *PtrCHLI1*/*2* double genes (Fig. S2e–f; Table S3). Cas9/gCHLI1/2‐a and ‐b caused many homozygous/biallelic mutations in both *PtrCHLI1* and *PtrCHLI2*, suggesting that knockout of double genes is as efficient as that of a single gene. However, Cas9/gCHLI1/2‐c and ‐d produced more mutations in *PtrCHLI1* than in *PtrCHLI2*, suggesting that gRNA specificity is crucial for editing efficiency. Six randomly selected T_0_ lines with Cas9/gRNA‐induced mutations were analyzed for each off‐target event. No mutation was detected in these potential off‐target sites among the 48 (six lines × eight putative off‐target sites) sequenced samples (Table S4; Methods S1).

**Fig. 1:**
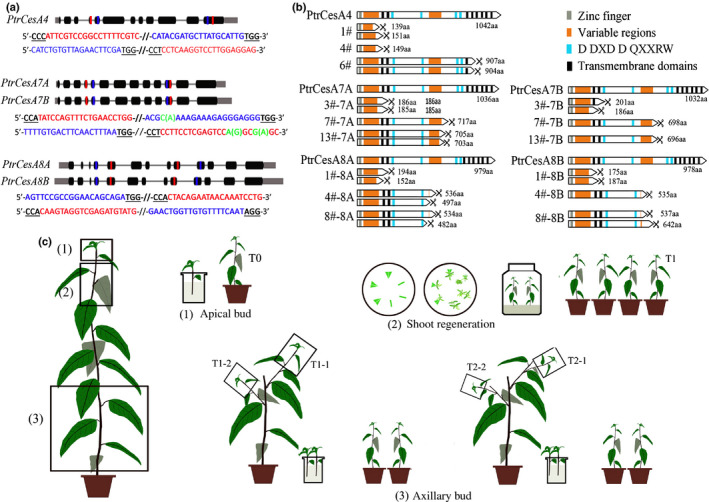
Cas9/gRNA‐induced mutations in *PtrCesA4*, *PtrCesA7A/B*, and *PtrCesA8A/B* genes of *Populus trichocarpa* (CesA, cellulose synthase). (a) Twelve gRNAs were designed in *PtrCesA4*, *PtrCesA* *7A* */B* and *PtrCesA* *8A* */B* genes. Nucleotides in blue and red represent the target sites. (b) The deduced amino acids of protein‐coding regions from the Cas9/gRNA‐edited genes in nine putative *ptrcesa* knockout mutants (*ptrcesa4‐1#*, *−4#* and − *6#*, *ptrcesa* *7a* */b‐3#*, −*7#* and − *13#*, *ptrcesa* *8a* */b‐1#*, −*4#* and − *8#*). The scissors indicate protein‐coding termination. (c) Three asexual propagation methods (apical bud cloning, axillary bud cloning and shoot regeneration) were used to generate progeny from the *ptrcesa* mutants with the Cas9/gRNA‐induced mutations. The apical buds and axillary buds were rooted hydroponically and planted in soils.

In order to investigate the roles of SCW *CesA*s in trees, the loss‐of‐function mutations in *PtrCesA4*, *7A*, 7*B*, *8A* and *8B* were performed using the Cas9/gRNA gene editing method. Two pairs of gRNAs were selected for each class of SCW *PtrCesA*s to generate multiple mutants (Figs 1a, S3a). After detection of the edited target sites, a total of six, seven, eight, 13, seven, five and eight mutation lines were obtained for the *PtrCesA4*, *7A*, *7B*, *7A/B*, *8A*, *8B* and *8A/B* genes, respectively (Table S5). In most cases, nucleotide deletions and insertions at target sites by Cas9/gRNA cause frameshift mutations in protein‐coding sequences, which result in the knockout of the target gene. According to the amino acids deduced from the coding sequence, three lines (1#, 4# and 6#) with different mutations showed a putative knockout of *PtrCesA4*, and multiple knockouts of double genes were generated in *PtrCesA7A/B* and *PtrCesA8A/B*, respectively (Fig. 1b). Likewise, knockout of *PtrCesA7A*, *7B*, *8A* or *8B* gene was shown in a number of the mutant lines (Fig. S3b–c; Table S5). Furthermore, the *ptrcesa* mutation lines were analyzed for off‐target assays, and no mutation was detected at the potential off‐target sites (Table S6; Methods S1), suggesting the specificity of the selected gRNA for each *PtrCesA*.

We assessed the inheritance of Cas9/gRNA‐induced mutations in the progeny of *ptrcesa* mutants. Two T_0_ lines arbitrarily selected from *ptrcesa4*, *ptrcesa7ab* or *ptrcesa8ab* mutants served as mother plants for generating the progeny by three asexual propagation methods (Fig. 1c; Table S7). Of the 100 sites sequenced, 99 edited sites in *PtrCesA4*, *7A/B* and *8A/B* did not change in apical and axillary bud clones, suggesting faithful transmission of the mutations to the next generation (Table S8). A considerable proportion (22 of 60) of new mutations arose in target sites of the progeny propagated by the shoot regeneration method (Table S8). Thus, apical and axillary bud cloning is optimal for asexual propagation of Cas9/gRNA‐induced mutations in *P*.* trichocarpa*.

### Analysis of the SCW PtrCesA protein concentrations in the Cas9/gRNA‐induced *ptrcesa* mutants

**Fig. 2:**
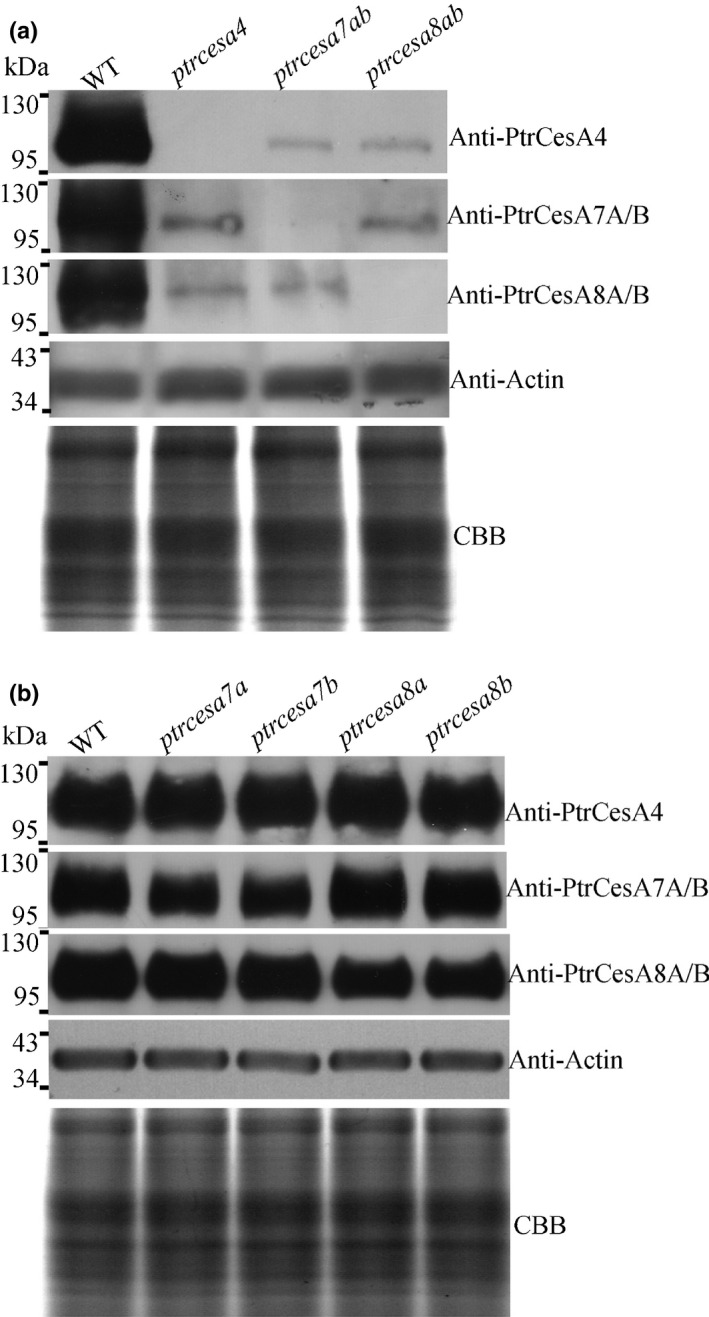
Analysis of the secondary cell wall (SCW) cellulose synthase PtrCesA protein concentrations in the Cas9/gRNA‐induced *Populus trichocarpa ptrcesa* knockout mutants. (a) Immunoblot analysis of PtrCesA4, PtrCesA7A/B and PtrCesA8A/B protein concentrations in secondary xylem of wild‐type (WT), *ptrcesa4*, *ptrcesa7ab* and *ptrcesa8ab* young trees. (b) Immunoblot analysis of PtrCesA4, PtrCesA7A/B and PtrCesA8A/B protein concentrations in secondary xylem of WT, *ptrcesa7a*, *ptrcesa7b*, *ptrcesa8a* and *ptrcesa8b* young trees. The ACTIN as control was detected using an anti‐Actin antibody (ab197345, Abcam), indicating equal loading proteins. A replicate Coomassie Brilliant Blue (CBB)‐stained gel is shown to confirm equal loading.

In order to examine the PtrCesA4, 7A/B and 8A/B protein concentrations in the Cas9/gRNA‐induced *ptrcesa* mutants, we produced anti‐PtrCesA4, anti‐PtrCesA7A/B and anti‐PtrCesA8A/B antibodies as a tool. Two specific peptides for each class of the SCW PtrCesAs were synthesized as antigens to raise antibodies in rabbits (Fig. S4a,b). As a result, we obtained effective anti‐PtrCesA4, anti‐PtrCesA7A/B and anti‐PtrCesA8A/B polyclonal antibodies, respectively (Fig. S4c). Western blot analysis showed high concentrations of PtrCesA4, 7A/B or 8A/B proteins in WT xylem and their molecular weights of *c*. 118, 117 and 110 kDa (Fig. 2). However, PtrCesA4, 7A/B and 8A/B proteins were undetectable in putative *ptrcesa4*, *ptrcesa7a/b* and *ptrcesa8a/b* knockout mutants (Fig. 2a), suggesting that they are null mutants. In addition, the abundance of PtrCesA7A/B (or PtrCesA8A/B) proteins decreased significantly in putative *ptrcesa7a* and *ptrcesa7b* (or *ptrcesa8a* and *ptrcesa8b*) knockout mutants (Fig. 2b).

In comparison with the WT, the concentrations of PtrCesA7A/B and 8A/B proteins decreased sharply in *ptrcesa4* knockout mutants, and likewise, those of PtrCesA4 and 8A/B (or 7A/B) proteins were significantly reduced in *ptrcesa7a/b* (or *ptrcesa8a/b*) knockout mutants (Fig. 2a). These data indicate that deletion of one class of the SCW PtrCesAs (PtrCesA4, 7A/B or 8A/B) diminished the protein concentrations of the other two classes of the SCW PtrCesAs. In addition, PtrCesA7A and PtrCesA7B (or PtrCesA8A and PtrCesA8B) with presumed functional redundancies had no obvious mutual complementation at protein concentrations in the corresponding *ptrcesa* mutants (Fig. 2b). In addition, RT‐PCR analysis showed that the transcriptional level of each SCW *PtrCesA* moderately decreased in the corresponding knockout mutant, but those of other SCW*PtrCesAs* did not change significantly in the mutant (Fig. S5; Methods S2).

### *Ptrcesa4*, *7a/b* and *8a/b* knockout mutants exhibit similar morphological abnormalities

**Fig. 3:**
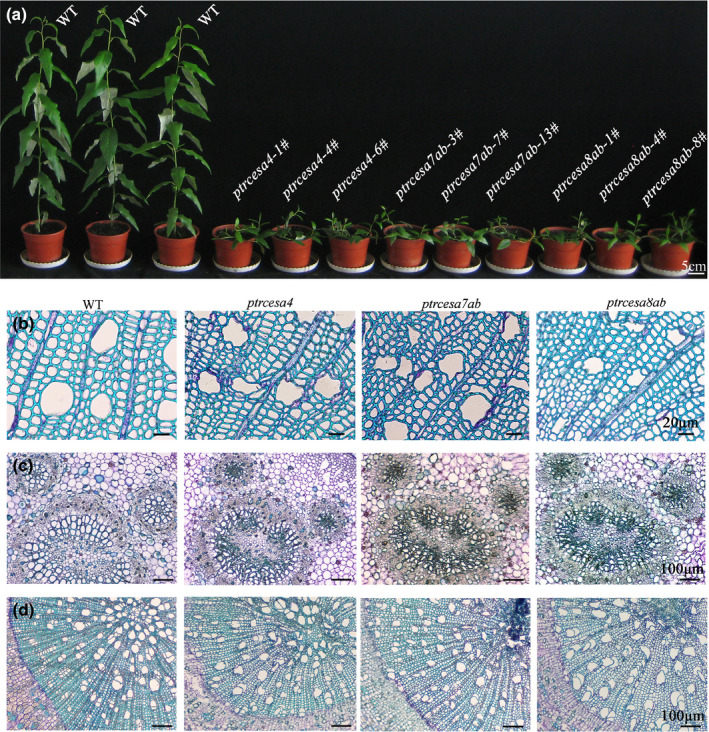
Phenotypes of *Populus trichocarpa* *ptrcesa4*, *ptrcesa7a*/*b*, and *ptrcesa8a*/*b* mutants (CesA, cellulose synthase). (a) Morphology of multiple *ptrcesa* mutant and wild‐type (WT) trees grown for 3 months in a glasshouse. (b‐d) Light microscopic analysis of stems (b), petioles (c) and roots (d) from 3‐month‐old *ptrcesa* mutant and WT trees. Sections were stained with toluidine blue. Stem cross‐sections are from the 10^th^ internode; petiole cross‐sections are from the 8^th^ leaf below the apical bud. Bars: (a) 5 cm; (b) 20 μm; (c–d) 100 μm.

Knockout of *PtrCesA4* caused serious defects in growth and development in transgenic trees (Fig. 3a). The *ptrcesa4* mutants showed complete prostrate growth and formed twisted stems. After *c*. 6 months of growth in the glasshouse, the *ptrcesa4* mutants completely lost apical dominance, resulting in weeping woody plants (Fig. S6b). The stems of *ptrcesa4* were extremely brittle, revealing major changes in stem mechanical properties (Fig. S6d). In addition, the *ptrcesa4* mutants showed significant reductions in stem diameter, internode length, leaf size and root structure, and significantly decreased the sizes of the pavement and guard cells in the leaves (Fig. S7; Methods S3). The *ptrcesa7a*/*b* (or *ptrcesa8a*/*b*) double mutants presented the same defects as the *ptrcesa4* mutants (Figs 3a, S6, S7), implying similar and nonredundant roles of *PtrCesA4*, *7A/B* and *8A/B* in *P*.* trichocarpa*. Compared with the *ptrcesa7a*/*b* double mutant, the knockouts of *ptrcesa7a* or *7b* showed slight growth defects (Fig. S6a,c), indicating redundant roles for *PtrCesA7A* and *7B*. Likewise, the redundant roles of *PtrCesA8A* and *8B* were suggested by comparative phenotypes of single and double mutants.

**Fig. 4:**
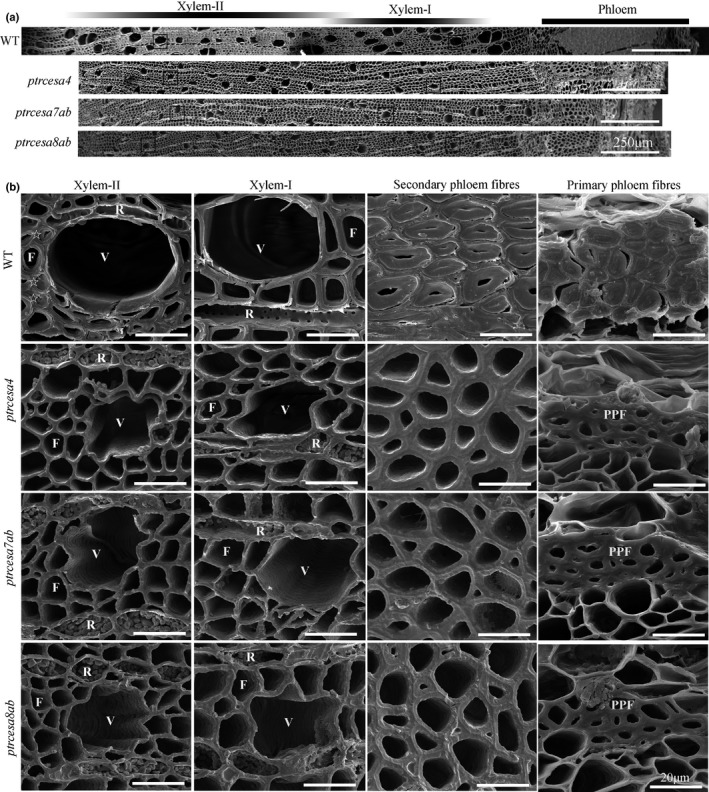
Scanning electron microscopy analysis of stem tissues in *Populus trichocarpa* *ptrcesa4*, *ptrcesa7a/b* and *ptrcesa8a/b* mutants (CesA, cellulose synthase). (a) Scanning electron microscopy (SEM) images of cross‐sections of basal stems from 6‐month‐old wild‐type (WT) and *ptrcesa* mutant trees. (b) Xylem fibres, vessels, and ray cells within inset boxes in the (a) and secondary and primary phloem fibres (SPF and PPF) in the WT and these mutants were magnified under SEM. Some mature wood fibres (indicated by pentangles) have developed the thick G‐layers inside the secondary cell walls (SCWs). Xylem‐I, developing xylem; Xylem‐II, mature xylem; V, vessel cell; F, fibre cell; R, ray cell. Bars: (a) 250 μm; (b) 20 μm.

Light microscopy analysis showed that the stem xylem was severely collapsed in *ptrcesa4*, *7a*/*b* and *8a*/*b* mutants but not in *ptrcesa7a*, *7b*, *8a* and 8*b* mutants (Figs 3b, S8, S9). The vessel cells of the 2^nd^ to 4^th^ stem internodes undergoing predominantly primary growth, leaf petioles, and root tissues also displayed collapse in these mutants (Figs 3c,d, S8), which might be the main reason for impaired leaf and root growth. In addition, the inner surfaces of pith parenchyma cell walls of these mutants were undulating (or folded) and had few pits, whereas those of the WT were smooth and had numerous ones (Fig. S10). Approximately 3‐month‐old mutants in the glasshouse showed earlier accumulation of starch granules in pith ray and parenchyma cells, whereas > 6‐month‐old WT trees were capable of accumulating starch granules in these cells (Figs 4, S10), suggesting that the SCW *PtrCesAs‐*mediated cellulose synthesis is a key pathway that is integrated into carbohydrate metabolism in trees. Thus, the above complete loss‐of‐function phenotypes indicate that *PtrCesA4*, *7A*/*B* and *8A*/*B* play crucial, similar and nonredundant roles in the growth and development of *P*.* trichocarpa*.

### The crucial roles of *PtrCesA4*, *7A/B* and *8A/B* in wood cell wall structure

**Fig. 5:**
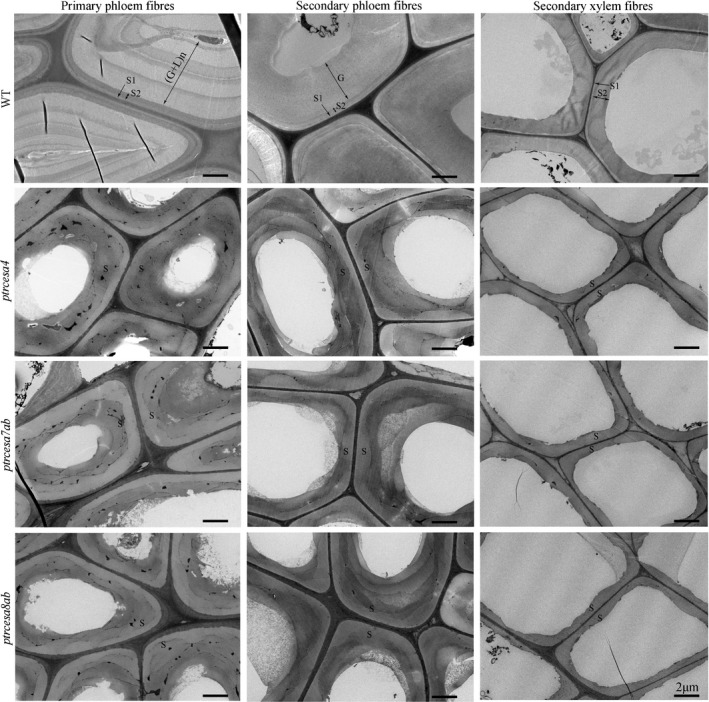
Transmission electron microscopy analysis of the phloem and wood fibre wall structures in *Populus trichocarpa ptrcesa4*, *ptrcesa7a/b* and *ptrcesa8a/b* mutants (CesA, cellulose synthase). Transmission electron microscopy (TEM) images from the basal stem phloem and xylem fibres in 6‐month‐old wild‐type (WT) and *ptrcesa* mutant trees. The WT primary and secondary phloem fibres showed S1 + S2 + *n*(G + L) and S1 + S2 + G wall structures, respectively. L, lignified layer; G, gelatinous (G)‐layer; *n*, number of repetitions of the G and L; S, S‐layer of SCW with S1, S2 and S3. Bars, 2 μm.

We explored the roles of *PtrCesA4*, *7A/B* and *8A/B* in wood wall structure using SEM analysis. As shown in Fig. 4, the wood fibres of WT xylem‐I (developing xylem) developed the SCWs, and some wood fibres of xylem‐II (mature xylem) produced a thick G‐layer (indicated by the pentangle) inside the SCW. However, in addition to the severe collapse of wood fibres and vessels, thin one‐layer‐walled fibres were observed in the xylem‐I and xylem‐II of *ptrcesa4*, *7a*/*b* and *8a*/*b* mutants. Further TEM analysis clearly showed a multilayered SCW structure in WT wood fibres with visible S1 and S2 layers, whereas these mutants displayed thin one‐layer‐walled fibres in woods (Fig. 5). The thickness of wood fibre walls in these mutants was reduced by approximately half compared with the WT, and the lengths of the mutant wood fibres and vessels were significantly shorter than those of the WT (Figs S11, S12; Methods S4). These data indicate that *PtrCesA4*, *7A/B* and *8A/B* are essential for a multilayered SCW structure of wood in *Populus*.

**Fig. 6:**
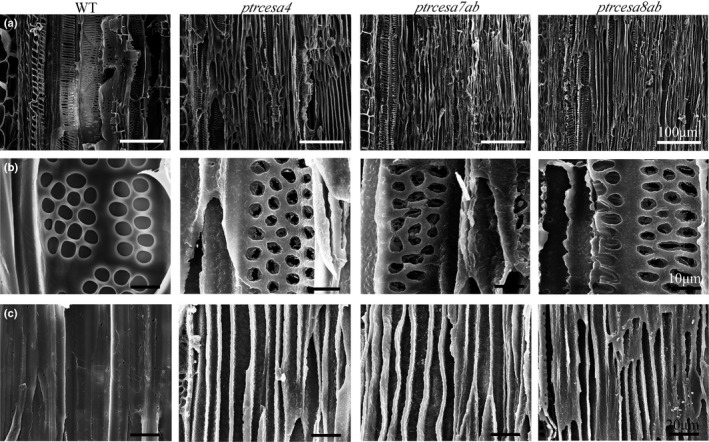
Scanning electron microscopic images of xylem fibres and vessels in longitudinal sections of basal stems from *Populus trichocarpa* wild‐type (WT) and *ptrcesa4*, *7a* */b* and *8a* */b* mutants (CesA, cellulose synthase). (a‐c) Primary and secondary xylem (a), the pitted pattern vessels of secondary xylem (b) and the fibres of secondary xylem (c). Bars: (a) 100μm; (b) 10 μm; (c) 20 μm.

Furthermore, we examined the inner surfaces of fibre and vessel cell walls in longitudinal sections of the mutant wood under SEM. From the pith parenchyma outwards spiral and annular vessels were observed in protoxylem (primary xylem) of WT stems, and subsequently, reticulate and pitted vessels were observed in the metaxylem (secondary xylem) (Fig. 6a). Overall, the mutants also developed these types of vessels in protoxylem and metaxylem, whereas the stem xylem was severely collapsed (Fig. 6a). Nevertheless, the inner surfaces of vessel walls in the mutant woods were undulating and uneven, and pit membranes were broken in the mutant wood vessels (Fig. 6b), suggesting that SCW cellulose is an essential component. Likewise, the fibre wall features (wall pits and their patterns) were significantly impaired in the mutant woods (Fig. 6c). Taken together, loss of *PtrCesA4*, *7A/B* or *8A/B* leads to serious damage to fibre and vessel wall structure in Populus.

### *PtrCesA4*, *7A/B*, and *8A/B* are indispensable for G‐layer formation of TW fibres in *Populus*

**Fig. 7:**
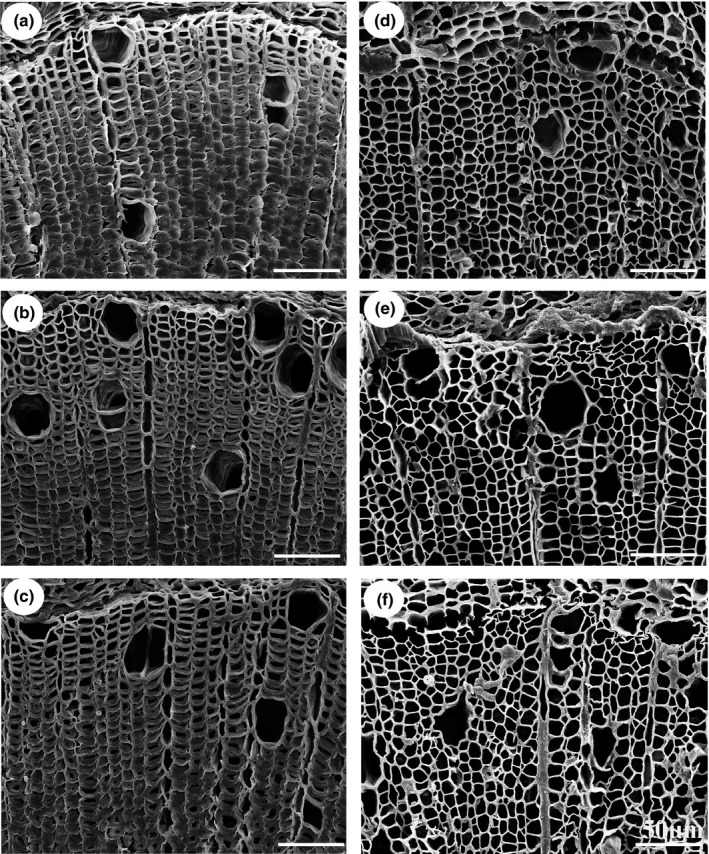
Scanning electron microscopy analysis of tension wood (TW) gelatinous (G)‐fibres in *Populus trichocarpa* wild‐type (WT) and *ptrcesa* mutants under gravi‐stimulation (CesA, cellulose synthase). (a–f) The WT (a), and *ptrcesa8a* (b), *ptrcesa8b* (c), *ptrces8ab* (d), *ptrces7ab* (e) and *ptrces4* (f) mutants grown straight in a glasshouse for 4 months were inclined to induce TW by a 45˚ angle from the vertical direction for 10 d. The scanning electron microscopy (SEM) images were taken from freehand cross‐sections of the 16^th^ internode of each sample. Bars, 50 μm.

We investigated the roles of the SCW *PtrCesAs* in G‐layer formation of *Populus* TW fibres at genetic level. In the leaning stem of young WT trees, the TW side showed eccentric growth, a reduction in the number of vessels and the production of G‐fibres under SEM (Figs 7, S13), which have been considered as the main TW features. Likewise, the first two features were observable in the TW sides of *ptrcesa4*, *ptrcesa7ab* and *ptrcesa8ab* mutants (Fig. S13), indicating a primary response to the incline‐induced gravity stimuli. However, no G‐layer was visible in any of the TW fibres of the *ptrcesa4*, *ptrcesa7ab* and *ptrcesa8ab* mutants (Fig. 7). In addition, the ability of G‐layer formation was slightly impaired in *ptrcesa8a* or *ptrcesa8b* mutants, suggesting a redundant role of *PtrCesA8A* and *8B* in this aspect. These data indicate that *PtrCesA4*, *7A/B* and *8A/B* are indispensable for the G‐layer formation of TW fibres.

**Fig. 8:**
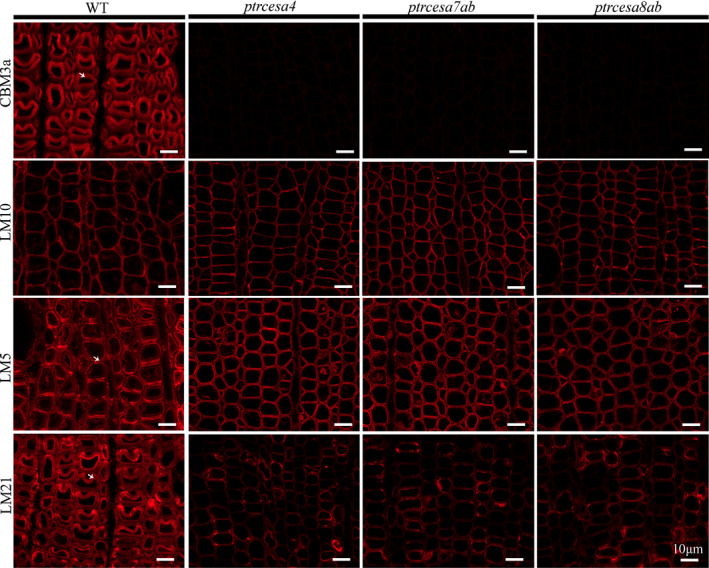
Immunolocalization of crystalline cellulose, xylan, β‐(1 → 4)‐galactan and mannan in the tension wood (TW) side in *Populus trichocarpa* wild‐type (WT), and *ptrcesa4*, *ptrcesa7ab* and *ptrcesa8ab* mutants (CesA, cellulose synthase). The 8‐μm transverse cross‐sections of the 16^th^ internode from each sample were incubated with carbohydrate‐specific antibody (LM10/5/21) or CBM3a‐6 × His protein and anti‐His antibody. Signals (red) were detected with Alexa Fluor 633 goat anti‐rat IgG. CBM3a, LM10, LM5 and LM21 bind crystalline cellulose, xylan, β‐(1 → 4)‐galactan and mannan in plant cell walla, respectively. Arrowheads indicate TW fibre gelatinous (G)‐layers labelled with immunofluorescence in the WT. Bars, 10 μm.

Furthermore, the distribution of cell wall polymers was examined using the fluorescent immunolabelling technique in the mutant TW and opposite wood (OW). Crystalline cellulose, confirmed by CBM3a immunolabelling, was distributed intensively in the G‐layer of TW fibres and SCWs of OW in the WT, but nearly invisible in TW and OW of these mutants (Figs 8, S14), suggesting that PtrCesA4, 7A/B and 8A/B synthesized complete crystalline cellulose in SCWs of OW and TCWs of TW. Using LM10 to detect xylan (a main noncellulosic polysaccharide of the S‐layer), the fluorescence outlines were narrowed in S‐layers of the mutant OW compared with the WT, but those were likewise thin in S‐layers of TW in the WT and mutants (Figs 8, S15), implying that loss of crystalline cellulose impaired SCW structure in OW, but did not affect SCW thinning during TW. The β‐(1 → 4)‐galactan‐rich G‐layers of TW (Gorshkova *et␣al*., 2015) were not detected in WT and mutant OW, and conversely, strong fluorescence signals appeared in fibres of mutant TW without TCWs, which was similar to that observed in TW of the WT (Figs 8, S16). In addition, the immunofluorescence for detecting mannan was weak in fibres of the mutant TW but strong in those of the WT (Figs 8, S17), indicating that loss of crystalline cellulose impairs deposition of mannans in TCWs of TW.

### The roles of *PtrCesA4*, *7A/B* and *8A/B* in stem phloem fibre wall structures

In order to investigate the role of the SCW PtrCesAs in phloem fibres, we observed wall structures in *ptrcesa4*, *ptrcesa7ab* and *ptrcesa8ab* mutants under SEM. The WT primary and secondary phloem fibres (PPFs, SPFs) showed extremely thick walls with multilayers and S + G layers, respectively (Fig. 4b). However, the mutant SPF showed a larger lumen, lost the G‐layer, but retained the thick wall with no obvious collapse (Fig. 4b). The mutant PPF also lost the multilayered structure and retained the thicker wall, and cell size was greatly reduced (Fig. 4b). Under TEM, WT SPF showed S1 + S2 + G layers in the wall, and interestingly, the PPF developed an unique wall structure with S1 + S2 + *n*(G + L) layers, where G and L layers are formed alternately (Fig. 5). By contrast, the mutant SPF and PPF lost S1 + S2 + G and S1 + S2 + *n*(G + L) wall structures and both remained a thick wall with 3–5 μm thickness, even exceeding that of SCWs in WT wood fibres (Figs 5, S11). The thick walls of the mutant SPF and PPF should be composed of S‐layers, where the boundaries of S1 and S2 layers were indistinguishable under TEM.

**Fig. 9:**
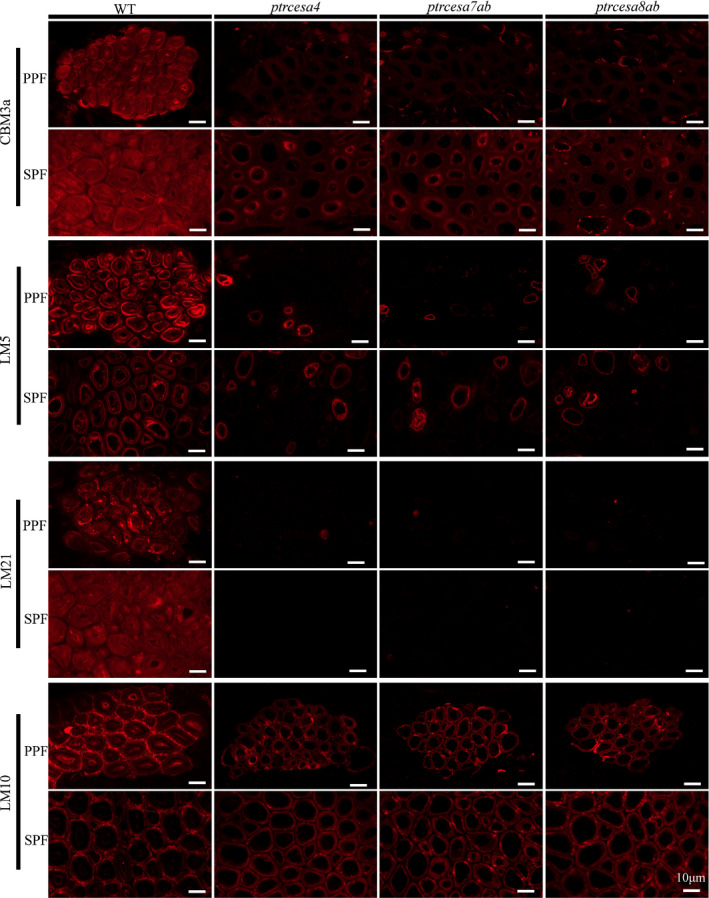
Immunolocalization of crystalline cellulose, xylan, β‐(1 → 4)‐galactan and mannan in phloem fibres in *Populus* *trichocarpa* wild‐type (WT), and *ptrcesa4*, *ptrcesa7ab* and *ptrcesa8ab* mutants (CesA, cellulose synthase). Primary and secondary phloem fibers (PPF, SPF) in the 8‐μm transverse cross‐sections of the 20^th^ internode from each sample were incubated with carbohydrate‐specific antibody (LM10/5/21) or CBM3a‐6 × His protein and anti‐His antibody. Signals (red) were detected with Alexa Fluor 633 goat anti‐rat IgG. CBM3a, LM10, LM5 and LM21 bind crystalline cellulose, xylan, β‐(1 → 4)‐galactan and mannan in plant cell walls, respectively. Bars, 10 μm.

We further analyzed the distribution of cell wall polymers in the mutant phloem fibres. The S + G layers of SPF and S + *n*(G + L) layers of PPF in the WT showed strong CBM3a‐immunolabelled fluorescence (indicating crystalline cellulose), and conversely, the signals in the mutant SPF and PPF were weak but could be detected (Figs 9, S18). LM5‐immunolabelled fluorescence revealed rich β‐(1 → 4)‐galactan in G‐layers of SPF and *n*(G + L) layers of PPF in the WT, whereas most of the mutant SPFs and PPFs had no significant deposition of β‐(1 → 4)‐galactan in the walls, but only several SPFs (or PPFs) in each phloem fibre region showed clear deposition of β‐(1 → 4)‐galactan (Figs 9, S19). In addition, signals indicating mannans did not appear in the mutant SPF and PPF (Figs 9, S20), whereas they were visible (but weak) in the mutant TW fibres (Fig. 8). Xylan‐immunolabelled signals gathered strongly in S‐layers of WT SPF and PPF and were distributed moderately in *n*(G + L) layers of PPF. In the mutants, both SPFs and PPFs losing the G/*n*(G + L) layers showed wider fluorescence outlines (Figs 9, S21), suggesting a thicker S‐layer in the walls. Overall, the findings show that knockout of *PtrCesA4*, *7A/B* or *8A/B* leads to similar but differential wall structures in wood and phloem fibres.

### Contributions of *PtrCesA4*, *7A/B* and *8A/B* to wood chemical composition

We examined the chemical composition of the wood walls in the *ptrcesa4*, *7a*/*b* and *8a*/*b* mutants (Table 1). The cellulose content of the wood of these mutants, as calculated by the Updegraff method, were *c*. 4%, one‐tenth of that in WT wood (*c*. 41%), indicating that *PtrCesA4*, *7A/B* and *8A/B* are essential for wood cellulose synthesis. By contrast, wood lignin content as calculated by the Klason or acetyl bromide soluble lignin method were increased nearly two‐fold in these mutants compared with the WT, and the phloroglucinol staining data were in agreement with this result (Fig. S22). Compared with *ptrcesa7a*/*b* (or *ptrcesa8a*/*b*) double mutants, the decrease in cellulose content and increase in lignin content in wood were not drastic in *ptrcesa7a* and 7*b* (or *ptrcesa8a* and *8b*) mutants (Fig. S23), implying the redundant role of *PtrCesA7A* and *7B* (or *PtrCesA8A* and *8B*) in wood cellulose synthesis.

**Table 1:** Cellulose, lignin and noncellulosic polysaccharides in dry wood of *Populus trichocarpa* wild‐type (WT) and *ptrcesa* mutants (CesA, cellulose synthase).

Wood composition	WT	*ptrcesa4*	*ptrcesa7ab*	*ptrcesa8ab*
Cellulose (%)				
Crystalline	41.36 ± 0.62	3.88 ± 0.11***	3.89 ± 0.06***	4.03 ± 0.15***
Lignin (%)				
Klason	20.36 ± 0.41	39.02 ± 0.79***	39.38 ± 0.51***	39.45 ± 0.41***
Acid‐soluble	2.19 ± 0.06	2.12 ± 0.04	2.11 ± 0.06	2.13 ± 0.05
Total lignin	22.83 ± 0.36	41.14 ± 0.75***	41.49 ± 0.45***	41.58 ± 0.49***
Acetyl bromide soluble lignin	22.20 ± 0.55	41.62 ± 0.92***	42.09 ± 0.88***	42.13 ± 0.92***
Polysaccharide^a^				
Xylose	203.29 ± 5.63	505.52 ± 10.48***	507.39 ± 10.77***	504.26 ± 6.10***
Rhamnose	4.38 ± 0.19	8.12 ± 0.15***	8.24 ± 0.16***	8.44 ± 0.24***
Fucose	1.02 ± 0.04	1.32 ± 0.04***	1.33 ± 0.04***	1.32 ± 0.03***
Arabinose	4.15 ± 0.06	9.87 ± 0.09***	9.93 ± 0.09***	9.45 ± 0.09***
Galactose	8.61 ± 0.27	10.95 ± 0.28***	10.38 ± 0.29***	11.06 ± 0.17***
Mannose	14.62 ± 0.22	5.13 ± 0.11***	4.84 ± 0.09***	4.73 ± 0.07***
Glucose	56.15 ± 1.98	13.18 ± 0.20***	11.33 ± 0.18***	13.59 ± 0.40***

Values are means ± SD (*n* = 3). Asterisks denote significant difference from WT by Student’s *t*‐test (***, *P* < 0.001). ^a^Data are shown as µg mg^−1^ cell wall residues (AIRs).

The content of xylose, which is a major monosaccharide of hemicelluloses (xylan), increased 2.5‐fold in *ptrcesa4*, *7a*/*b* and *8a*/*b* mutants, and other low concentrations of monosaccharides (rhamnose, arabinose, fucose and galactose) increased significantly (Table 1), indicating a large amount of hemicellulose accumulation in these mutant woods. However, the mannose and glucose contents of these mutant woods decreased three‐to four‐fold compared with those in the WT (Table 1), which might suggest a significant decrease in mannans and/or glucomannans by the loss of *PtrCesA4*, *7A/B* or *8A/B*.

## Discussion

Approximately half of the woody biomass synthesized by trees is cellulose. In *P*. *trichocarpa*, five cellulose synthases PtrCesAs (PtrCesA4, 7A, 7B, 8A and 8B) are classified into the secondary cell wall (SCW) CesA group (Kumar *et␣al*., 2009). Here, the defective phenotypes of the Cas9/gRNA‐induced gene knockouts show that the set of SCW *PtrCesAs* plays a crucial role in growth and development, multilayered wall structure and wood chemical composition. In addition, each class of the SCW *PtrCesAs* is essential for developing wood gelatinous (G)‐fibres, and our findings suggest differences in the role of SCW *PtrCesAs*‐synthesized cellulose in the wall architecture of wood and phloem fibres. Overall, this functional understanding of the SCW *PtrCesAs* provides further insights into the impact of lacking cellulose biosynthesis on growth, SCW, wood G‐fibre and phloem fibre wall structures in the tree.

Genetic cessation of SCW cellulose biosynthesis can impair growth and development in transgenic *Populus* trees. Here, the *ptrcesa4*, *7a/b* and *8a/b* knockout mutants exhibited serious and similar morphological abnormalities. A recent study reported similar and moderate defects in *PtrCesA* RNAi transgenic plants in *P*. *trichocarpa* (Abbas *et␣al*., 2020). Serious defects of these null mutants exhibited prostrate growth, loss of apical dominance and significant reductions in stem diameters, internode lengths and leaf sizes (Figs 3, S6, S7). Small organs of these mutants suggest the possible roles of *PtrCesA4*, *7A/B* and *8A/B* in cell‐size determination, as manifested by the decrease in sizes of wood fibres and vessels, pith parenchyma, and guard and pavement cells (Figs S7g, S10, S12). Although the shapes and sizes of plant cells are defined largely by their surrounding walls (Bögre *et␣al*., 2008), plant cell expansion should not be undermined by the reduced secondary cellulose. This is because cell expansion generally terminates before SCW deposition. It is very likely that water deficit led to smaller organs in these mutants. Lack of secondary cellulose gave rise to serious collapse in vessel cells of leaf petioles and xylem of stems and roots in these mutants (Figs 3b–d, 6a, S8), which might, in turn, reduce xylem water transport capacity. In *Arabidopsis cesa7* mutants, stem xylem is partially collapsed and the small guard cells result from the decreased water supply to the developing leaves (Liang *et␣al*., 2010). In our mutants (of *ptrcesa4*, *7a/b* and *8a/b*), the loss of SCW cellulose also resulted in broken pit membranes in the xylem vessels (Fig. 6b). Pits are essential components in the water transport system and pit membranes account for *c*. 50% of the hydraulic resistivity of the water transported through xylem conduits (Sperry *et␣al*., 2006; Choat *et␣al*., 2008).

The SCW cellulose synthesis could be one of the key pathways integrated into plant carbohydrate metabolism, especially in trees. Starch, as one of the main nonstructural carbohydrates, is stored in the wood parenchyma as long‐term reserves. During spring growth or in adverse environments, starch is mobilized or converted to sucrose for physiological metabolism in trees (Richardson *et␣al*., 2013; Bellasio *et␣al*., 2014; Yoshimura *et␣al*., 2016). Deletion of PtrCesA4, 7A/B or 8A/B caused earlier accumulation of starch granules in the mutant stem pith rays and parenchyma (Figs 4b, S10), implying that the cessation of SCW cellulose synthesis directs the flow of carbohydrates into starch as a sink. The sucrose synthase (SuSy) associated with the CesA complex (CSC) supplies uridine diphosphate (UDP)‐glucose as a substrate for CesAs, and overexpression of the cotton SuSy results in increased cellulose production in poplar (Coleman *et␣al*., 2009; Fujii *et␣al*., 2010). It is possible that owing to a lack of conversion of UDP‐glucose into cellulose, excessive UDP‐glucose forms a feedback signal and is redirected to starch synthesis in these mutants. We speculate that perturbation of SCW cellulose synthesis would cause pleiotropic effects in complex carbohydrate metabolism in trees.

The wood fibre SCW contains three layers (S1–S3) with different cellulose microfibril (CMF) orientations. Complete knockout of *PtrCesA4*, *7A/B* or *8A/B* disrupted the multilaminar wall structure and formed thin one‐layer‐walled fibres in the wood (Figs 4, 5). Lack of cellulose destroys the multilayer formation of SCWs in mutant wood, presumably due to serious CMF deficiency. It is known that the deficiency of CMFs in SCWs alters the physical properties of wood, as demonstrated by excessive stem brittleness and severe wood vessel and fibre collapse in the mutants (Figs 4, 8, S6). Although *Arabidopsis* does not produce wood, the *AtCesA8*, *7* and *4* mutations (*irx1*, *3* and *5*) have shown irregular xylem (irx) phenotypes (Taylor *et␣al*., 1999, 2000; 2003). The collapsed phenotypes caused by deletion of SCW *CesA*s in *Populus* are more severe than those in *Arabidopsis*. The interfascicular fibres in the *irx1*, *3* or *5* mutants showed no obvious collapse or distortion, possibly analogous to retention of the S‐layers in phloem fibres of the *ptrcesa* mutants. Notably, PtrCesA4, 7A/B and 8A/B contribute to complete wood SCW cellulose synthesis because deletion of any of the three classes reduced wood cellulose content to 4% (Table 1), which is inferred to represent plant cell wall (PCW) cellulose.

The formation of wood G‐fibres in trees responds to gravistimulation, and gravitropism and reaction wood are the organizing centres for this biological process (Groover, 2016). The *ptrcesa4*, *7a/b* and *8a/b* mutants undergoing gravistimulation showed asymmetrical radial growth, thinned the S‐layers in fibres but lost the G‐layers (Figs 7, 8), implying that they have perceived gravistimulation and produced tension wood (TW). It is inferred that PtrCesA4, 7A/B and 8A/B act as basal components in this pathway, and the CMFs that they synthesize are the component core in TCWs of TW fibres. In addition, rhamnogalacturonan‐I (RG‐I), mannan and xyloglucan are the main polysaccharides in TW fibre TCWs (Nishikubo *et␣al*., 2007; Mellerowicz & Gorshkova, 2012). RG‐Is with shortened galactan chains are proposed as spacers between the CMFs to prevent their lateral interaction, potentially creating tension in mature G‐fibres (Gorshkova *et␣al*., 2015). Despite no CMFs of TCWs, the galactans still accumulated in the walls of the mutant TW fibres (Fig. 8), possibly because they interact with the polysaccharides of S‐layer. Additionally, losing CMFs of the TCWs reduced the deposition of mannans in walls of the mutant TW fibres (Fig. 8), seemingly suggesting the association of mannans with the CMFs. In the future, loss‐of‐functions of the genes encoding noncellulosic polysaccharide synthesis enzymes could provide further insights into the architecture of the polymers in TW fibres.

Mature phloem fibres endow structural strength and flexibility to plant stems. Some herbaceous plants normally develop thick G‐layers in mature phloem fibres (Roach *et␣al*., 2011). In the wild‐type (WT) *Populus* of the glasshouse, the mature primary and secondary phloem fibres (PPFs and SPFs) developed *n*(G + L)‐ and G‐layers in the walls, respectively (Figs 4, 5). Differential wall structures in both might be ascribed to different origins, that is, both initiate from the procambium and vascular cambium, respectively. Expression data show that flax phloem fibres recruit both PCW and SCW CesAs during deposition of TCWs (Mokshina *et␣al*., 2017). Here, both PPFs and SPFs lost individualized wall structures in these SCW *ptrcesa* mutants and remained similar thick walls without the TCWs. Accordingly, *PtrCesA4*, *7A/B* and *8A/B* are essential for developing *n*(G + L)‐/G‐layers of *Populus* phloem fibres. The synthesized CMFs are proposed as the skeleton to assemble the wall structures. Additionally, the phenotypes of these mutant phloem and TW fibres (Figs 4, 5, 8, 9) could suggest different roles of the SCW *PtrCesAs* in the two wall structures. First, the SPF walls accumulated a small amount of crystalline cellulose but TW fibres almost did not. Second, phloem fibres remained thicker walls (than TW fibres), which presumably comprised the S‐layers. Third, phloem fibres displayed weaker deposition of mannan and galactan on the walls (than TW fibres) when the two lost the TCWs in structure. Thus, it is proposed that differential wall deposit mode (or additional CesA‐like proteins) might be involved in SCW biosynthesis of phloem fibres.

In *Arabidopsis*, CesA4, 7 and 8 are the core components of the SCW CSC (Taylor *et␣al*., 2000, 2003) and exhibit varying degrees of class specificity (Kumar *et␣al*., 2017). The *ptrcesa4*, *7a/b* and *8a/b* knockout mutants exhibited similar defects in all phenotypes, including growth and development, SCW structure and wood composition (Figs 3, 4, 5, 6; Table 1), indicating that PtrCesA4, 7A/B and 8A/B are not redundant and represent the three classes of SCW CesAs. Deletion of any one class in *Populus* caused a complete lack of wood SCW cellulose synthesis (Table 1), which implies destructive damage to SCW CSC. This notion is further supported by the evidence that deletion of one class of the SCW PtrCesAs exaggeratedly diminished protein concentrations in the other two classes (Fig. 2a). Similar results have been shown in *Arabidopsis cesa4*, *7* or *8* mutants, assuming that these mutants do not assemble a functional CSC (Atanassov *et␣al*., 2009). In this regard, the mode by which the *Populus* CSC employs the SCW CesA classes is similar to that of *Arabidopsis*, thus suggesting the conserved core architecture of SCW CSCs in plants, including trees. Different from those of *Arabidopsis*, *Populus* CesA7 and CesA8 classes each evolve two members owing to recent whole genome duplication events (Takata & Taniguchi, 2015). The phenotypes of single and double mutants (Figs 3, 7, S6, S9) suggested that PtrCesA7A and 7B (and PtrCesA8A and 8B) are redundant in function. Considering that *Populus* CesA7 (or CesA8) class has two members, we speculate that *Populus* SCW CSCs are diversified in the number, identity and arrangement of the PtrCesA units. Whether PtrCesA7A and 7B (or PtrCesA8A and 8B) could heterogeneously constitute a SCW CSC, remains to be identified. In addition, it is a question that a specific CSC is responsible for synthesizing CMFs of the TCWs. A quantitative proteomics study has proposed that the stoichiometries of aspen CesA8A/B‐4‐7A/B are 3 : 2 : 1 and 8 : 3 : 1 in the developing xylem and TW, respectively (Zhang *et␣al*., 2018). In the present study, no G‐layer of TW fibres in these null mutants (Figs 7, 8) indicates that PtrCesA4, 7A/B and 8A/B are indispensable for synthesizing the CMFs of TCWs. Therefore, it is still unclear how the five SCW CesAs of three classes constitute the CSC in *Populus*.

## Author contributions

WX and YC conceived and designed the research; WX, HC, SZ, JC, HJ, BZ, SC, CW, GT, CZ, LM, YZ and YC performed the experiments and analyzed data; YC provided the funding; WX and YC wrote the manuscript; and YC reviewed and edited the manuscript.

## Supporting information

**Fig.␣S1** Cas9/gRNA system and stepwise protocol in Nisqually‐1.
**Fig.␣S2** Cas9/gRNA‐induced mutations in *PtrCHLI1* gene and *PtrCHLI1/2* family genes of *P. trichocarpa*.
**Fig.␣S3** Cas9/gRNA‐induced mutations in PtrCesA7A, PtrCesA7B, PtrCesA8A and PtrCesA8B genes of *P. trichocarpa*.
**Fig.␣S4** Production of anti‐PtrCesA4, −7A/B and −8A/B polyclonal antibodies in rabbits.
**Fig.␣S5** Transcriptional levels of five SCW *PtrCesA* genes in *ptrcesa* mutants using reverse transcription (RT)‐PCR analysis.
**Fig.␣S6** Characterization of the *ptrcesa4*, *7a/b*, *8a/b*, *7a*, *7b*, *8a*, and *8b* mutants.
**Fig.␣S7** Observation of stem internodes, mature leaves, and roots of 3‐month‐old *ptrcesa4*, *7a*/*b* and *8a*/*b* mutants.
**Fig.␣S8** Anatomical analysis of different stem internodes from the WT, an *ptrcesa4*, *7a/b* and *8a/b* mutants.
**Fig.␣S9** Anatomical analysis of different stem internodes from the WT, and *ptrcesa7a*, *7b*, *8a* and *8b* mutants.
**Fig.␣S10** Observation of pith parenchyma in the WT, and *ptrcesa4*, *7a/b* and *8a/b* mutants.
**Fig.␣S11** Wall thickness of xylem and phloem fibres in the basal stems of 6‐month‐old WT and *ptrcesa* mutants.
**Fig.␣S12** Microscopic analysis of the disaggregated xylem fibres and vessels in the WT, and *ptrcesa4*, *7a/b* and *8a/b* mutants.
**Fig.␣S13** Induction of TW in the WT and *ptrcesa* mutants under gravi‐stimulation.
**Fig.␣S14** Immunolocalization of crystalline cellulose in TW and OW sides in the WT, and *ptrcesa4*, *7ab* and *8ab* mutants.
**Fig.␣S15** Immunolocalization of the xylan in TW and OW sides in the WT, and *ptrcesa4*, *7ab* and *8ab* mutants.
**Fig.␣S16** Immunolocalization of β‐(1 → 4)‐galactan in TW and OW sides in the WT, and *ptrcesa4*, *7ab* and *8ab* mutants.
**Fig.␣S17** Immunolocalization of the mannan in TW and OW sides in the WT, and *ptrcesa4*, *7ab* and *8ab* mutants.
**Fig.␣S18** Immunolocalization of crystalline cellulose in phloem fibres of the WT, and *ptrcesa4*, *7ab* and *8ab* mutants.
**Fig.␣S19** Immunolocalization of β‐(1 → 4)‐galactan in phloem fibres of the WT, *ptrcesa4*, *7ab* and *8ab* mutants.
**Fig.␣S20** Immunolocalization of the mannan in phloem fibres of the WT, and *ptrcesa4*, *7ab* and *8ab* mutants.
**Fig.␣S21** Immunolocalization of the xylan in phloem fibres of the WT, and *ptrcesa4*, *7ab* and *8ab* mutants.
**Fig.␣S22** Lignin phloroglucinol staining in the WT, and*ptrcesa4*, *7ab* and *8ab* mutants.
**Fig.␣S23** Crystalline cellulose and lignin content in wood of the WT, and *ptrcesa7a*, *7b*, *8a* and *8b* mutants.
**MethodsS1** Analysis of putative Cas9/gRNA off‐target sites.
**MethodsS2** RNA extraction and RT‐PCR analysis.
**MethodsS3** SEM of leaf epidermal cells.
**MethodsS4** Wood fibre and vessel cell length analysis.
**Table␣S1** Primers used in this study.
**Table␣S2** The Cas9/gRNA‐targeted mutations in a single *PtrCHLI1* gene of *P. trichocarpa*.
**Table␣S3** The Cas9/gRNA‐targeted mutations in the *PtrCHLI1* and *2* genes of *P. trichocarpa*.
**Table␣S4** Analysis of potential off‐target sites among the Cas9/gRNA‐induced *ptrch* *l* *i1* mutants.
**Table␣S5** The Cas9/gRNA‐targeted mutations in *PtrCesA4*, *7A*, *7B*, *8A*, *8B*, *7A* */B* and *8A* */B* genes of *P. trichocarpa*.
**Table␣S6** Analysis of potential off‐target sites among the Cas9/gRNA‐induced *ptrcesa* mutants.
**Table␣S7** Inheritance of the Cas9/gRNA‐induced mutations in progeny of *ptrcesa4*, *7a/b* and *8a/b* mutants through asexual propagation methods.
**Table␣S8** Inheritance of the Cas9/gRNA‐induced mutations in the progeny of the *ptrcesa* lines.
Please note: Wiley Blackwell are not responsible for the content or functionality of any Supporting Information supplied by the authors. Any queries (other than missing material) should be directed to the *New* *Phytologist* Central Office.

## Data Availability

The data used to support the findings of this study appeared in the submitted article and are available from the corresponding author.
