# Supplementary material for: Functional understanding of secondary cell wall cellulose synthases in Populus trichocarpa via the Cas9/gRNA‐induced gene knockouts
Source: New Phytol. 2021 Jun 10;231(4):1478–95. doi: 10.1111/nph.17338 (PMC8362133; doi:10.1111/nph.17338)
Supplement: Supplementary file 1 — Fig.␣S1 Cas9/gRNA system and stepwise protocol in Nisqually‐1. Fig.␣S2 Cas9/gRNA‐induced mutations in PtrCHLI1 gene and PtrCHLI1/2 family genes of P. trichocarpa. Fig.␣S3 Cas9/gRNA‐induced mutations in PtrCesA7A, PtrCesA7B, PtrCesA8A and PtrCesA8B genes of P. trichocarpa. Fig.␣S4 Production of anti‐PtrCesA4, −7A/B and −8A/B polyclonal antibodies in rabbits. Fig.␣S5 Transcriptional levels of five SCW PtrCesA genes in ptrcesa mutants using reverse transcription (RT)‐PCR analysis. Fig.␣S6 Characterization of the ptrcesa4, 7a/b, 8a/b, 7a, 7b, 8a, and 8b mutants. Fig.␣S7 Observation of stem internodes, mature leaves, and roots of 3‐month‐old ptrcesa4, 7a/b and 8a/b mutants. Fig.␣S8 Anatomical analysis of different stem internodes from the WT, an ptrcesa4, 7a/b and 8a/b mutants. Fig.␣S9 Anatomical analysis of different stem internodes from the WT, and ptrcesa7a, 7b, 8a and 8b mutants. Fig.␣S10 Observation of pith parenchyma in the WT, and ptrcesa4, 7a/b and 8a/b mutants. Fig.␣S11 Wall thickness of xylem and phloem fibres in the basal stems of 6‐month‐old WT and ptrcesa mutants. Fig.␣S12 Microscopic analysis of the disaggregated xylem fibres and vessels in the WT, and ptrcesa4, 7a/b and 8a/b mutants. Fig.␣S13 Induction of TW in the WT and ptrcesa mutants under gravi‐stimulation. Fig.␣S14 Immunolocalization of crystalline cellulose in TW and OW sides in the WT, and ptrcesa4, 7ab and 8ab mutants. Fig.␣S15 Immunolocalization of the xylan in TW and OW sides in the WT, and ptrcesa4, 7ab and 8ab mutants. Fig.␣S16 Immunolocalization of β‐(1 → 4)‐galactan in TW and OW sides in the WT, and ptrcesa4, 7ab and 8ab mutants. Fig.␣S17 Immunolocalization of the mannan in TW and OW sides in the WT, and ptrcesa4, 7ab and 8ab mutants. Fig.␣S18 Immunolocalization of crystalline cellulose in phloem fibres of the WT, and ptrcesa4, 7ab and 8ab mutants. Fig.␣S19 Immunolocalization of β‐(1 → 4)‐galactan in phloem fibres of the WT, ptrcesa4, 7ab and 8ab mutants. Fig.␣S20 Immunolocalization of the m [file NPH-231-1478-s001.pdf]

New Phytologist **Supporting Information**

Functional understanding of secondary cell wall cellulose synthases in Populus trichocarpa via the Cas9/gRNA-induced gene knockouts

Wenjing Xu, Hao Cheng, Siran Zhu, Jiyao Cheng, Huanhuan Ji, Baocai Zhang, Shenquan Cao, Chong Wang, Guimin Tong, Cheng Zhen, Liqiang Mu, Yihua Zhou, Yuxiang Cheng

Article acceptance date: 02 March 2021

Supplemental Figures

Fig. S1 Cas9/gRNA system and stepwise protocol in Nisqually-1.

Fig. S2 Cas9/gRNA-induced mutations in *PtrCHLI1* gene and *PtrCHLI1/2* family genes of *Populus trichocarpa.*

Fig. S3 Cas9/gRNA-induced mutations in *PtrCesA7A*, *PtrCesA7B*, *PtrCesA8A*, and *PtrCesA8B* genes of *Populus trichocarpa.*

Fig. S4 Production of anti-PtrCesA4, -7A/B, and -8A/B polyclonal antibodies in rabbits.

Fig. S5 Transcriptional levels of five SCW *PtrCesA* genes in *ptrcesa* mutants using RT-PCR analysis.

Fig. S6 Characterization of the *ptrcesa4*, *7a/b*, *8a/b*, *7a*, *7b*, *8a*, and *8b* mutants.

Fig. S7 Observation of stem internodes, mature leaves, and roots of 3-month-old *ptrcesa4*, *7a/b*, and *8a/b* mutants.

Fig. S8 Anatomic analysis of different stem internodes from WT, *ptrcesa4*, *7a/b*, and *8a/b* mutants.

Fig. S9 Anatomic analysis of different stem internodes from WT, *ptrcesa7a*, *7b*, *8a*, and *8b* mutants.

Fig. S10 Observation of pith parenchyma in WT, *ptrcesa4*, *7a/b*, and *8a/b* mutants.

Fig. S11 Wall thickness of xylem and phloem fibres in the basal stems of 6-month-old WT and *ptrcesa* mutants.

Fig. S12 Microscopic analysis of the disaggregated xylem fibres and vessels in WT, *ptrcesa4, 7a/b*, and *8a/b* mutants.

Fig. S13 Induction of tension wood (TW) in WT and *ptrcesa* mutants under gravi-stimulation.

Fig. S14 Immunolocalization of crystalline cellulose in tension and opposite wood (TW, OW) sides in WT, *ptrcesa4, 7ab*, and *8ab* mutants.
Fig. S15 Immunolocalization of the xylan in tension and opposite wood (TW, OW) sides in WT, *ptrcesa4, 7ab*, and *8ab* mutants.
Fig. S16 Immunolocalization of β-(1→4)-galactan in tension and opposite wood (TW, OW) sides in WT, *ptrcesa4, 7ab*, and *8ab* mutants.
Fig. S17 Immunolocalization of the mannan in tension and opposite wood (TW, OW) sides in WT, *ptrcesa4, 7ab*, and *8ab* mutants.
Fig. S18 Immunolocalization of crystalline cellulose in phloem fibres of WT, *ptrcesa4, 7ab*, and *8ab* mutants.
Fig. S19 Immunolocalization of β-(1→4)-galactan in phloem fibres of WT, *ptrcesa4*, *7ab* and *8ab* mutants.
Fig. S20 Immunolocalization of the mannan in phloem fibres of WT, *ptrcesa4*, *7ab*, and *8ab* mutants.
Fig. S21 Immunolocalization of the xylan in phloem fibres of WT, *ptrcesa4*, *7ab*, and *8ab* mutants.
Fig. S22 Lignin phloroglucinol staining in WT, *ptrcesa4, 7ab* and *8ab* mutants.
Fig. S23 Crystalline cellulose and lignin content in woods of WT, *ptrcesa7a*, *7b*, *8a*, and *8b* mutants.

Supplemental methods
Methods S1 Analysis of putative Cas9/gRNA off-target sites.
Methods S2 RNA extraction and RT-PCR analysis.
Methods S3 Scanning electron microscopy (SEM) of leaf epidermal cells.
Methods S4 Wood fibre and vessel cell length analysis.

Supplemental tables
Table S1. Primers used in this study.
Table S2. The Cas9/gRNA-targeted mutations in single *PtrCHLI1* gene of *Populus trichocarpa.*
Table S3. The Cas9/gRNA-targeted mutations in both *PtrCHLI1* and *2* genes of *Populus trichocarpa.*

Table S4. Analysis of potential off-target sites among the Cas9/gRNA-induced *ptrchli1* mutants.

Table S5. The Cas9/gRNA-targeted mutations in *PtrCesA4, 7A, 7B, 8A, 8B, 7A/B* and *8A/B* genes of *Populus trichocarpa*.

Table S6. Analysis of potential off-target sites among the Cas9/gRNA-induced *ptrcesa* mutants

Table S7. Inheritance of the Cas9/gRNA-induced mutations in progeny of *ptrcesa4*, *7a/b* and *8a/b* mutants through asexual propagation methods.

Table S8. Inheritance of the Cas9/gRNA-induced mutations in the progeny of the *ptrcesa* lines.

Fig. S1 Cas9/gRNA system and stepwise protocol in Nisqually-1. (a) Illustration of the cloning of two gRNA expression cassettes into the Cas9/gRNA binary vector using Golden Gate ligation. zCas9, Zea mays codon-optimized Cas9; U6-26p and U6-29p, two *Arabidopsis* U6 gene promoters; U6-26t, U6-26 terminator with the downstream sequence; SpR, spectinomycin resistance gene; Hyg, hygromycin resistance gene. (b) Production of hygromycin-resistant transgenic plantlets using *Agrobacterium*-mediated transformation of Nisqually-1. Scale bars show 1 or 5 cm. (c) Identification of transgenic plantlets by using a genomic DNA PCR analysis of the *zCas* and *Hyg* genes in all 16 transgenic lines (#) and wild-type (WT). *Actin2* was used as an internal control. (d) A flow chart of the Cas9/gRNA-based mutagenesis in Nisqually-1.

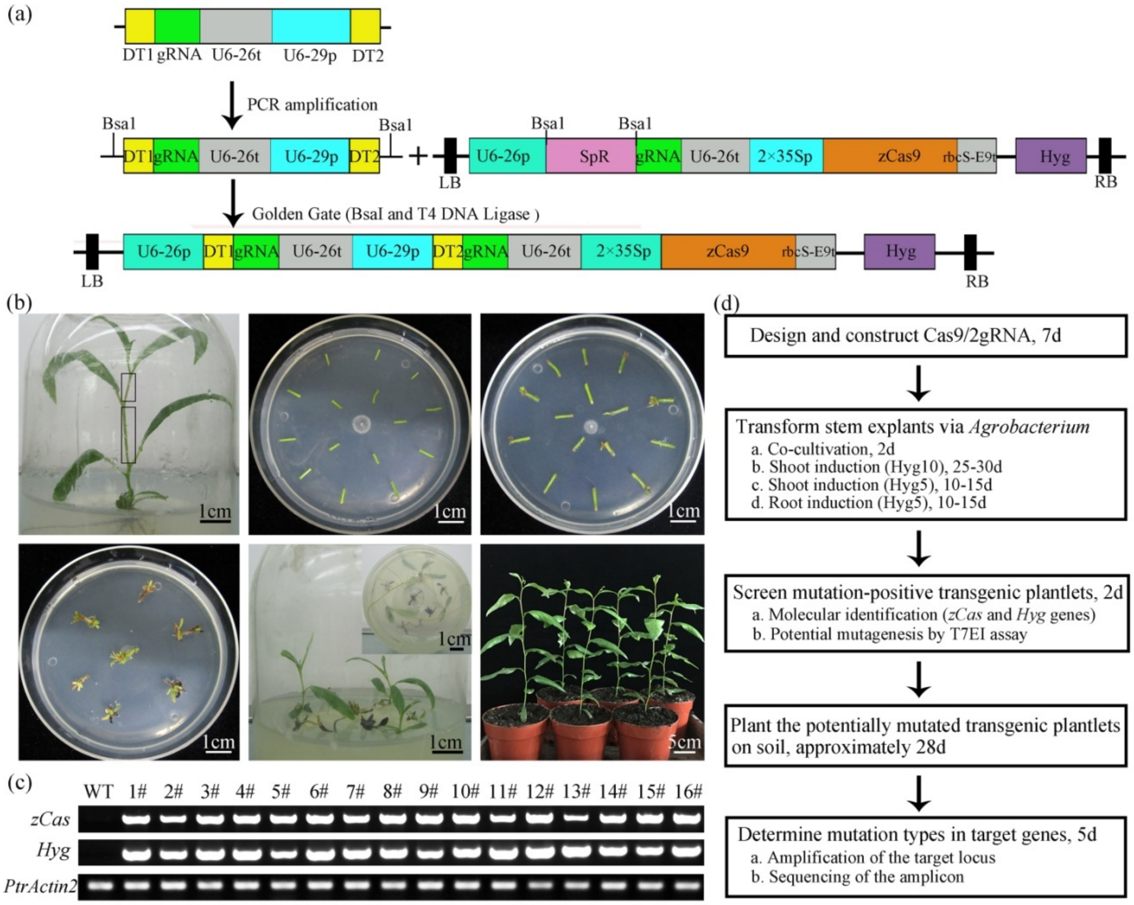

Fig. S2 Cas9/gRNA-induced mutations in *PtrCHLI1* gene and *PtrCHLI1/2* family genes of *Populus trichocarpa*. (a) Two gRNAs (gCHLI1-a and -b) were designed in the third exon of *PtrCHLI1* gene. Nucleotides in blue and red represent the two target sites. (b) Characterization of the target site mutations in 11 homozygous or diallelic lines (#) of all 18 T_0 transgenic plantlets. Dashes and lowercase letters represent the deletion and insertion of nucleotides. Mutation types are indicated on the right. (c) Cas9/gRNA-induced mutagenesis efficiencies in the single *PtrCHLI1* gene. (d) The phenotype of homozygous, diallelic, or heterozygous *ptrchli1* mutants mediated by Cas9/gRNA system. The scale bar shows 1 cm. (e) Two pairs of gRNAs (gCHLI1/2-a and -b; gCHLI1/2-c and -d) were designed in the third exon of the genes *PtrCHLI1* and *PtrCHLI2*, which were simultaneously targeted. (f) Cas9/gRNA-induced mutagenesis efficiencies in both *PtrCHLI1* and *PtrCHLI2* genes. TP and ML represent the transgenic plantlet and mutation lines, respectively.

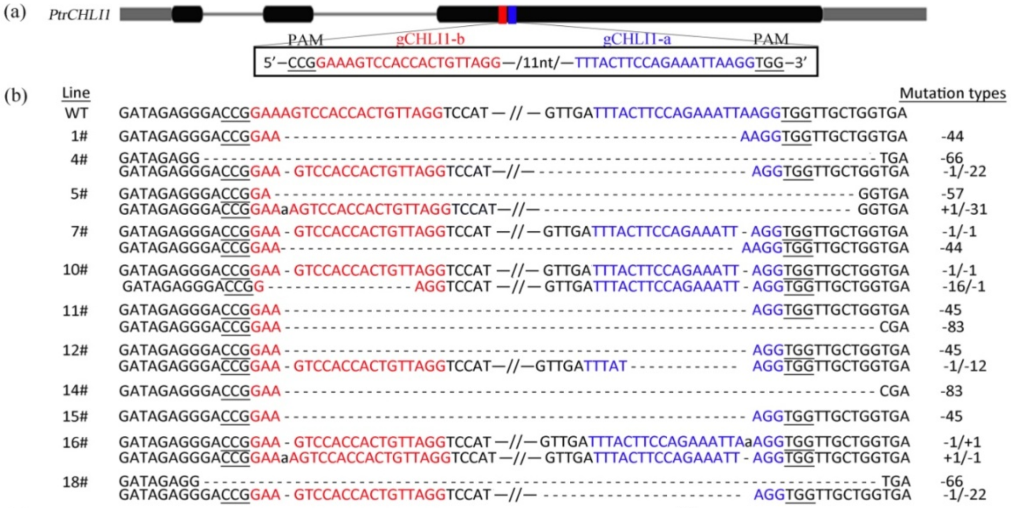

(c)

gRNA	Target gene	TP*	ML*	Mutation rate(%)	Homozygous mutation	Diallelic mutation	Heterozygous mutation	Chimera
gCHLI1-a	CHLI1	18	18	100.0	4 (22.2%)	7 (38.9%)	5 (27.8%)	2 (11.1%)
gCHLI1-b	CHLI1			100.0	3 (16.7%)	8 (44.4%)	5 (27.8%)	2 (11.1%)

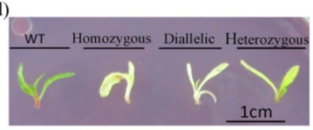

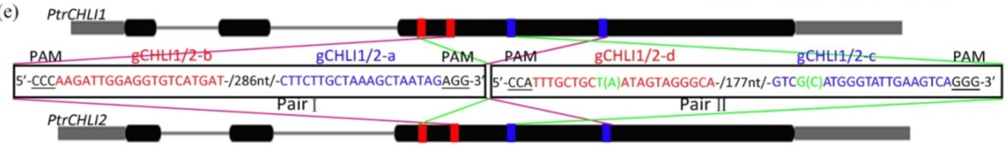

(f)

Pair	gRNA	Target gene	TP*	ML*	Mutation Rate(%)	Homozygous mutation	Diallelic mutation	Heterozygous mutation	Chimera	Wild type
Pair I	gCHLI1/2-a	CHLI1	12	12	100.0	8 (66.7%)	3 (25.0%)	0 (0.0%)	1 (8.3%)	0 (0.0%)
	gCHLI1/2-b	CHLI1			100.0	7 (58.4%)	4 (33.3%)	1 (8.3%)	0 (0.0%)	0 (0.0%)
	gCHLI1/2-a	CHLI2			100.0	2 (16.7%)	6 (50.0%)	2 (16.7%)	2 (16.7%)	0 (0.0%)
	gCHLI1/2-b	CHLI2			100.0	6 (50.0%)	5 (41.7%)	0 (0.0%)	1 (8.3%)	0 (0.0%)
Pair II	gCHLI1/2-c	CHLI1	8	8	100.0	2 (25.0)	5 (62.5%)	1 (12.5%)	0 (0.0%)	0 (0.0%)
	gCHLI1/2-d	CHLI1			75.0	1 (12.5)	3 (37.5%)	2 (25.0%)	0 (0.0%)	2 (25.0%)
	gCHLI1/2-c	CHLI2			87.5	0 (0.0%)	4 (50.0%)	2 (25.0%)	1 (12.5%)	1 (12.5%)
	gCHLI1/2-d	CHLI2			0.0	0 (0.0%)	0 (0.0%)	0 (0.0%)	0 (0.0%)	8 (100.0%)

Fig. S3 Cas9/gRNA-induced mutations in *PtrCesA7A*, *PtrCesA7B*, *PtrCesA8A*, and *PtrCesA8B* genes of *Populus trichocarpa*. (a) Sixteen gRNAs were designed in *PtrCesA7A*, *PtrCesA7B*, *PtrCesA8A* and *PtrCesA8B* genes. Nucleotides in blue and red represent the target sites. (b) Characterization of the target site mutations in 12 *ptrcesa* mutants (*ptrcesa7a-1#*, *-4#* and -7#; *ptrcesa7b*-2#, -4# and -5#; *ptrcesa8a-1#*, *-3#* and *-4#*; *ptrcesa8b-2#*, *-3#* and *-5#*). Dashes and lowercase letters represent the deletion and insertion of nucleotides. Mutation types are indicated on the right. (c) The deduced amino acids of protein-coding regions from the Cas9/gRNA-edited genes in 12 *ptrcesa* mutants (*ptrcesa7a-1#*, *-4#* and -7#; *ptrcesa7b*-2#, -4# and -5#; *ptrcesa8a-1#*, *-3#* and *-4#*; *ptrcesa8b-2#*, *-3#* and *-5#*). The scissors indicate protein-coding termination.

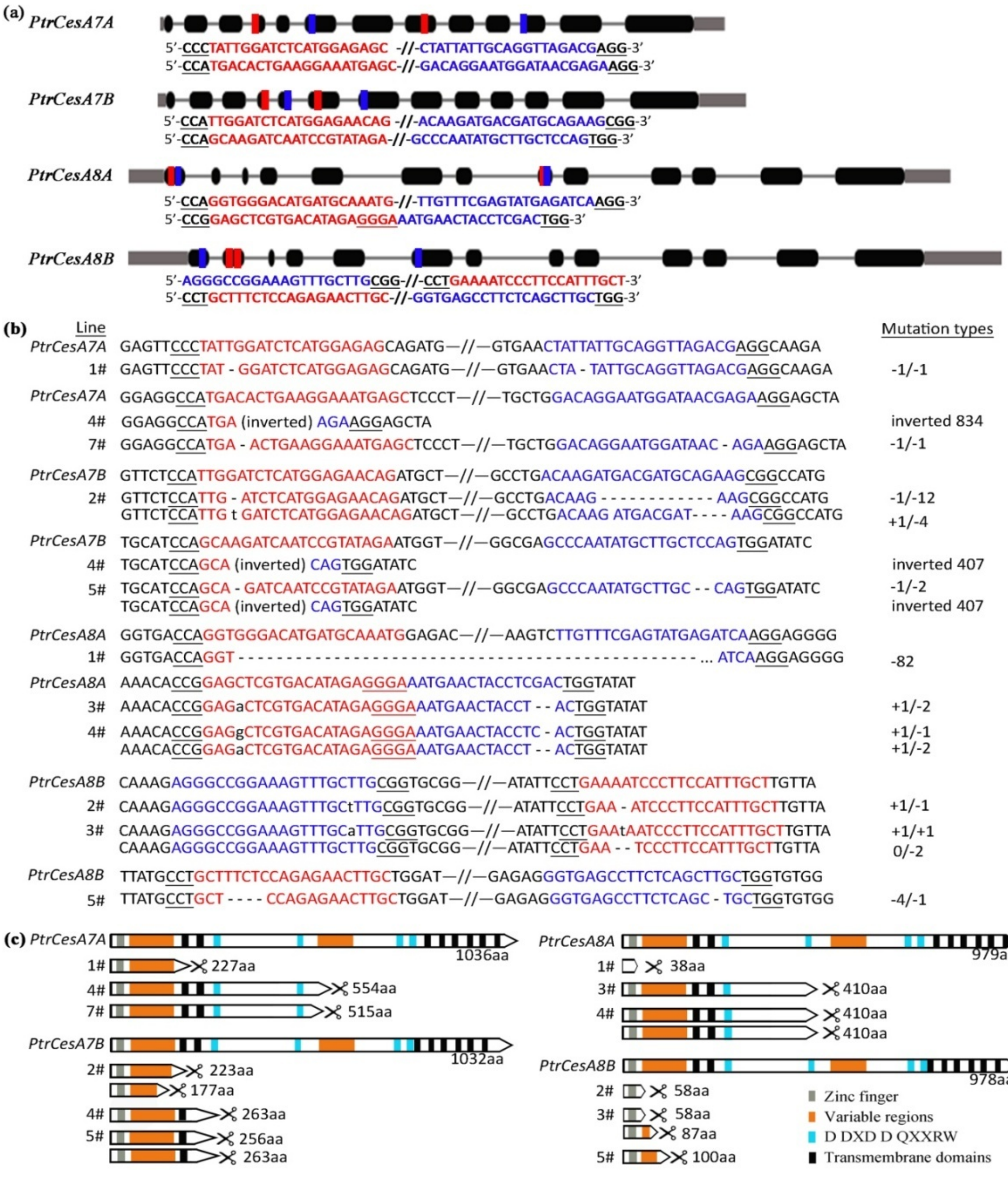

Fig. S4 Production of anti-PtrCesA4, -7A/B, and -8A/B polyclonal antibodies in rabbits. (a) The six specific peptides for each class of the SCW PtrCesAs (*PtrCesA4*, *7A/B*, and *8A/B*) were synthesized as antigens to raise antibodies in rabbits. The peptide with a checkmark produced the effective anti-PtrCesA antibody. (b) Alignment of the *PtrCesA4*, *7A*, *7B*, *8A*, and *8B* sequences. The peptides selected for antibody production are highlighted in yellow. (c) The SCW PtrCesA from xylem protein extracts in the wild-type *Populus* was specifically identified with the corresponding antibody by western blot analysis. The full sizes of the detected PtrCesAs (indicated with asterisks) were consistent with the predicted molecular weights.

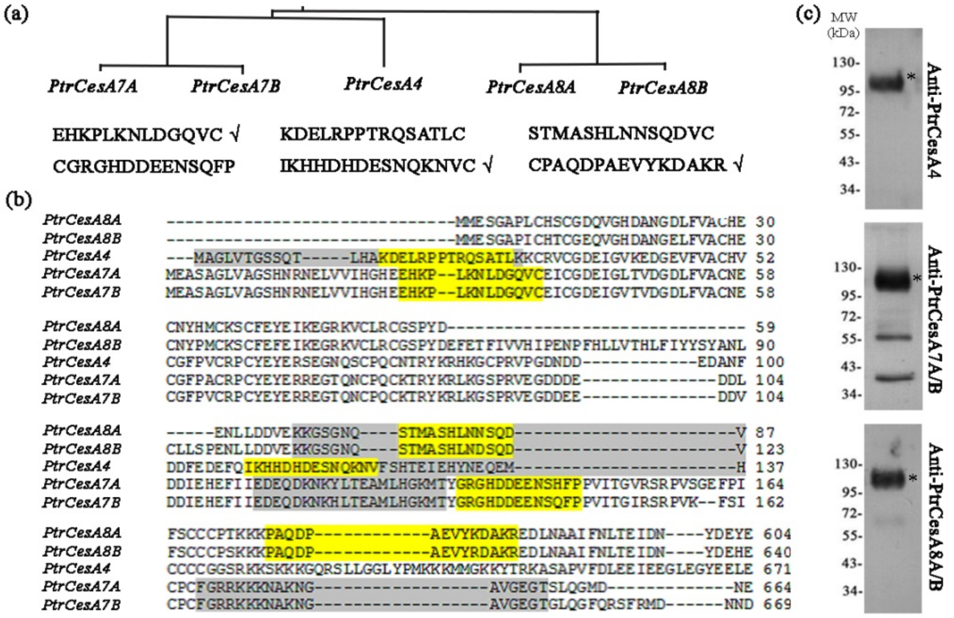

Fig. S5 Transcriptional levels of five SCW *PtrCesA* genes in WT and *ptrcesa* mutants using RT-PCR analysis. The expression of *PtrActin2* gene was used as a reference control.

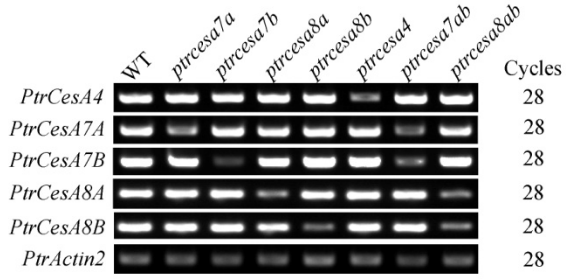

Fig. S6 Characterization of *ptrcesa4*, *7a/b*, *8a/b*, *7a*, *7b*, *8a*, and *8b* mutants. (a) Morphology of *ptrcesa7a*, *7b*, *8a*, *8b*, and wild-type (WT) young trees grown for 3 months in a greenhouse. (b) 6-month-old *ptrcesa4*, *7a/b* and *8a/b* mutant trees grown in a greenhouse. (c-d) Brittleness properties in stems of WT and *ptrcesa* mutants. Scale bars: (a-b) 5 cm; (c-d) 1 cm.

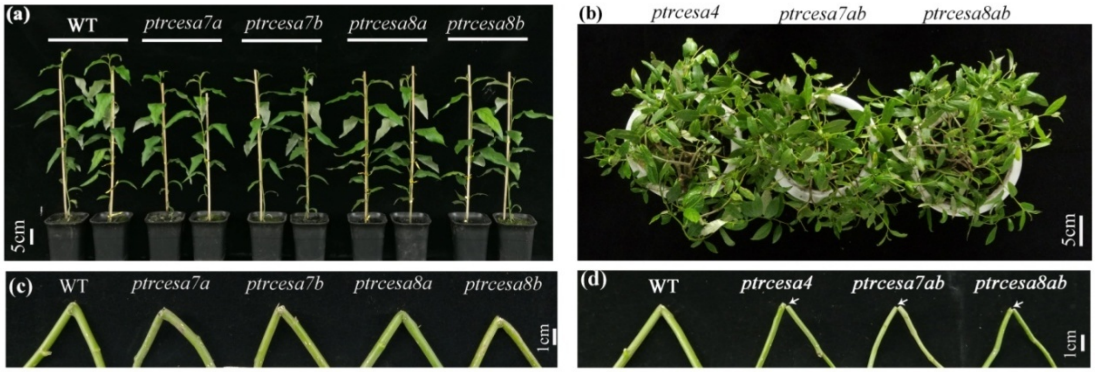

Fig. S7 Observation of stem internodes, mature leaves, and roots of 3-month-old *ptrcesa4*, *7a*/*b*, and *8a*/*b* mutants. (a-b) Stem diameter and internode length of wild-type (WT) and *ptrcesa* mutants. (c-d) Mature leaves and leaf areas of WT and *ptrcesa* mutants. (e-f) Roots and root biomass of WT and *ptrcesa* mutants. (g) Scanning electron microscopy of leaf epidermal cells in WT and *ptrcesa* mutants. The pavement cells were indicated in red. Scale bars: (c, e) 2 cm; (g) 50 μm. Values are means ± SD (n = 3). Asterisks denote significant difference from WT by Student's *t*-test: ***P* < 0.01, ****P* < 0.001.

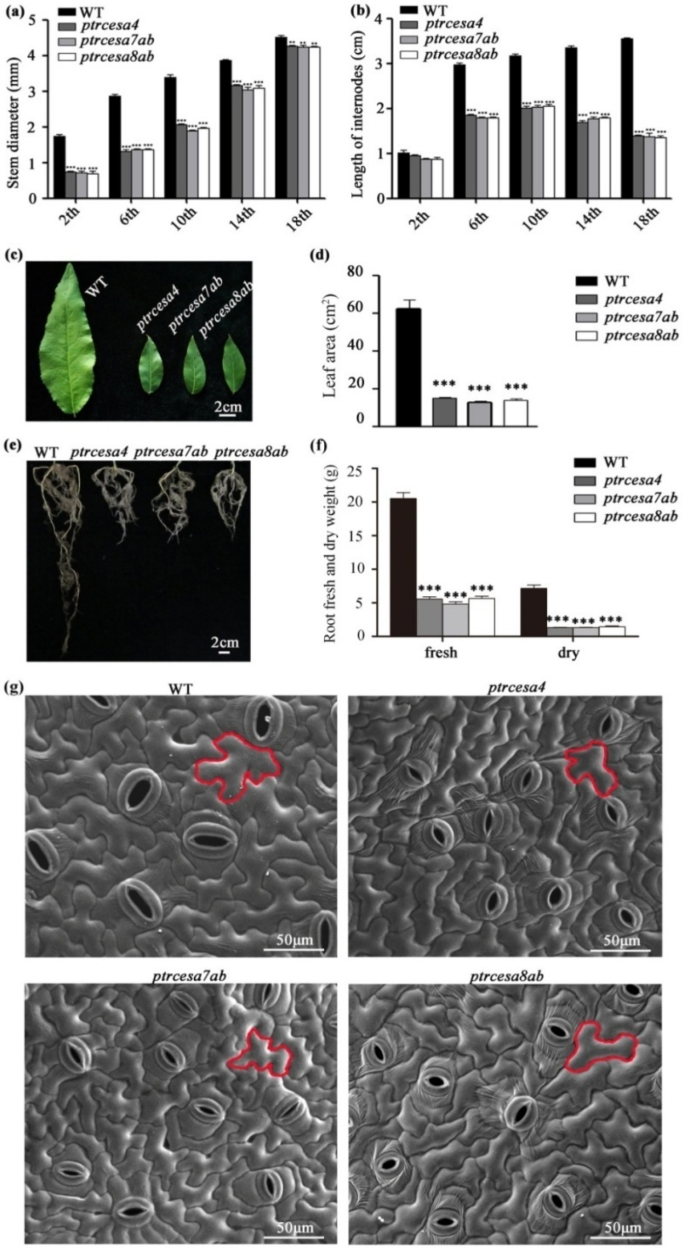

Fig. S8 Anatomic analysis of different stem internodes from WT, *ptrcesa4*, *7a/b*, and *8a/b* mutants. Cross-sections from the 2nd, 4th, 6th, 8th, 10th, 12th, 14th 16th, and 18th stem internodes (IN) were stained with toluidine blue. Scale bars: 200 μm.

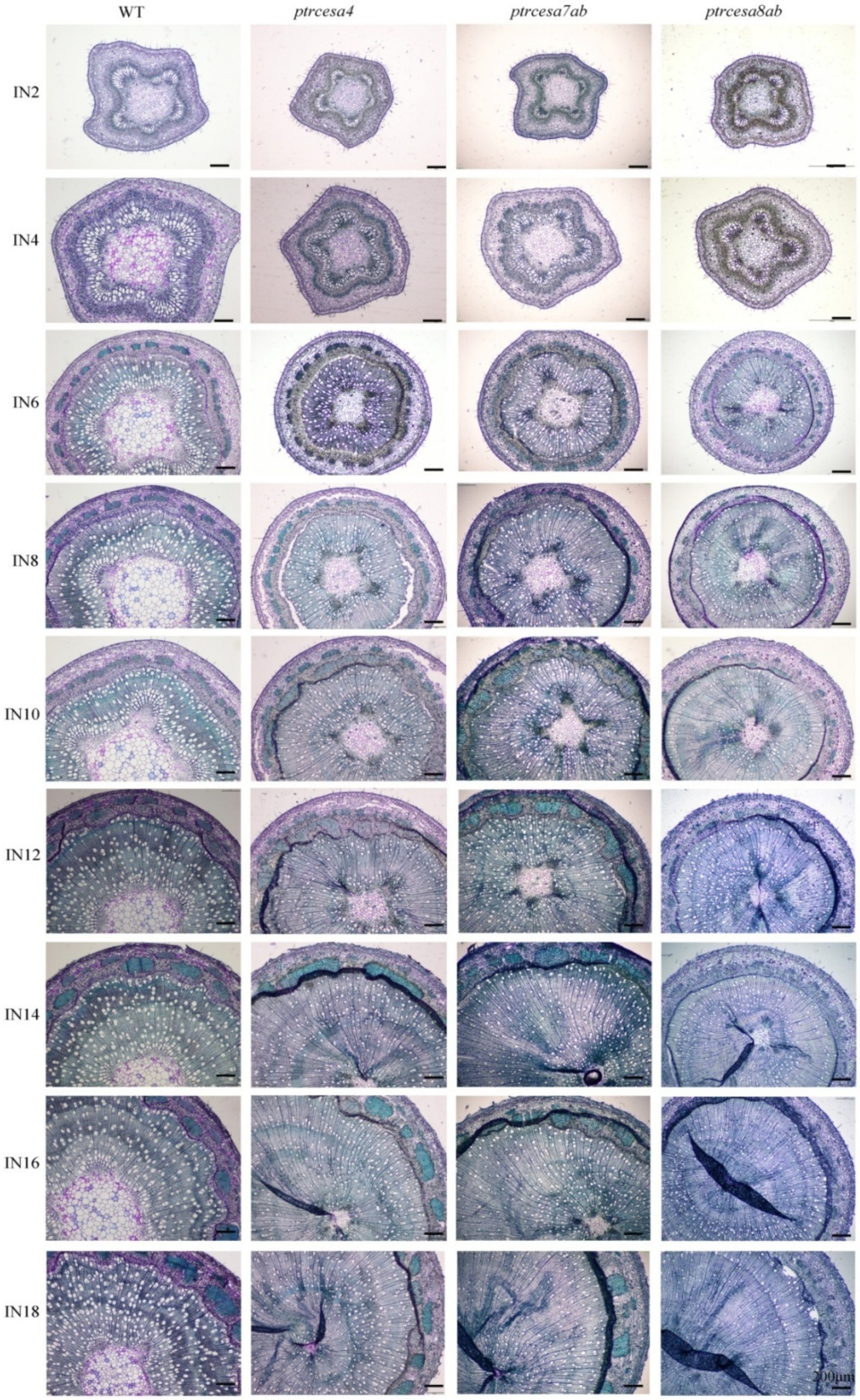

Fig. S9 Anatomic analysis of different stem internodes from WT, *ptrcesa7a*, *7b*, *8a*, and *8b* mutants. Cross-sections from the 10th, 14th 16th, and 18th stem internodes (IN) were stained with toluidine blue. Scale bars: 100 μm.

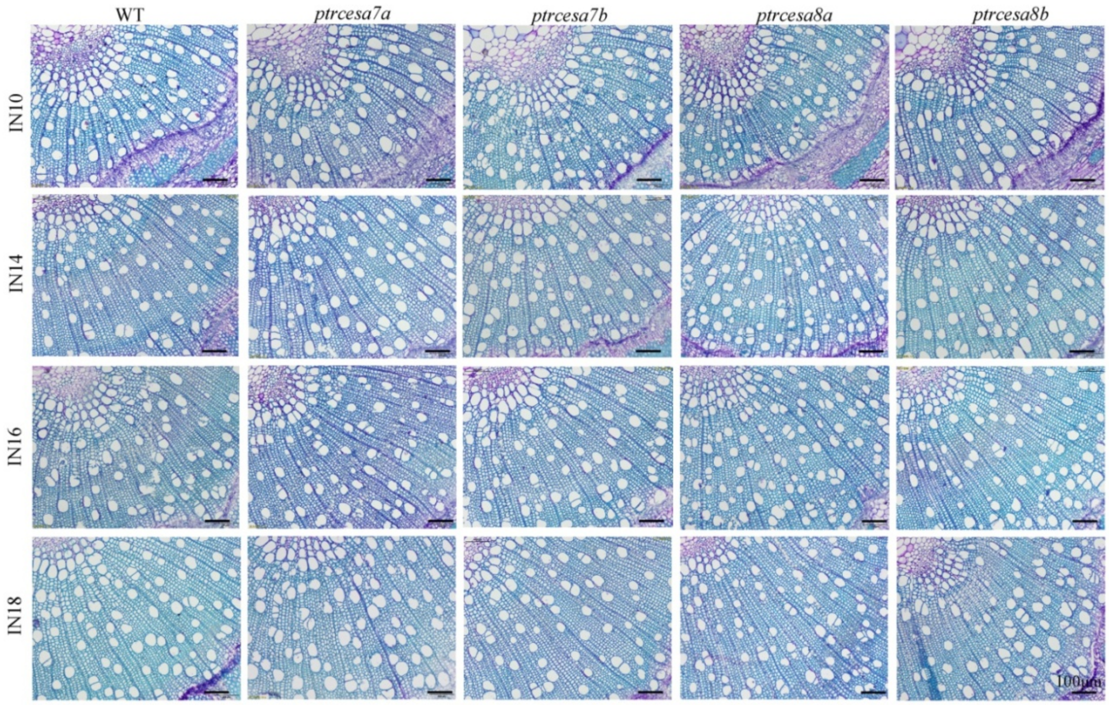

Fig. S10 Observation of pith parenchyma in WT, *ptrcesa4*, *7a/b*, and *8a/b* mutants. Scanning electron microscopy images of pith parenchyma in basal stem cross-sections of *ptrcesa* mutant and wild-type (WT) young trees. Upper and lower panels were from 2-month-old and 3-month-old *ptrcesa* mutant and WT trees, respectively. Scale bars: 20 μm.

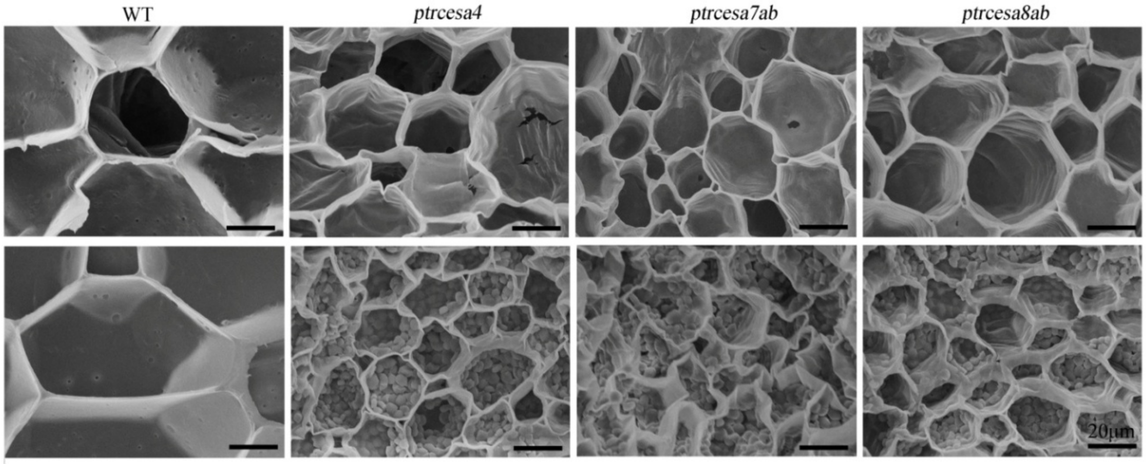

Fig. S11 Wall thickness of xylem and phloem fibres in the basal stems of 6-month-old WT and *ptrcesa* mutants. Xylem-I and -II indicated the developing and mature xylem, respectively. Values are means ± SD of three replicates. Each replicate contained ~100 measurements. Asterisks denote a significant difference from WT by Student's t-test: ***$P < 0.001$.

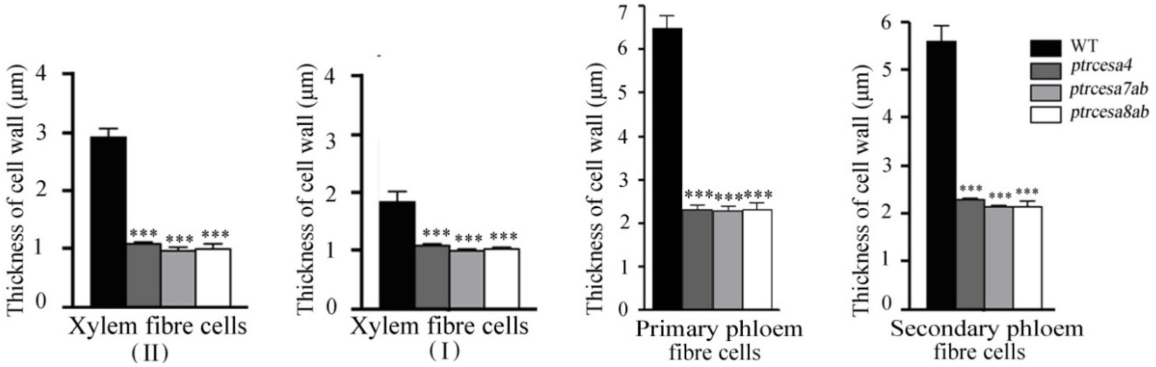

Fig. S12 Microscopic analysis of the disaggregated xylem fibres and vessels in WT, *ptrcesa4*, *7a/b*, and *8a/b* mutants. (a-b) Microscopic images of xylem fibers and vessels in the 20th stem internodes of the WT and *ptrcesa* mutants. Scale bars: 100 µm. (c) The length of xylem fibers and vessels in WT and *ptrcesa* mutants. Values are means ± SD of three replicates. Each replicate contained ~100 measurements. Asterisks denote significant difference from WT by Student's *t*-test: ***$P < 0.001$.

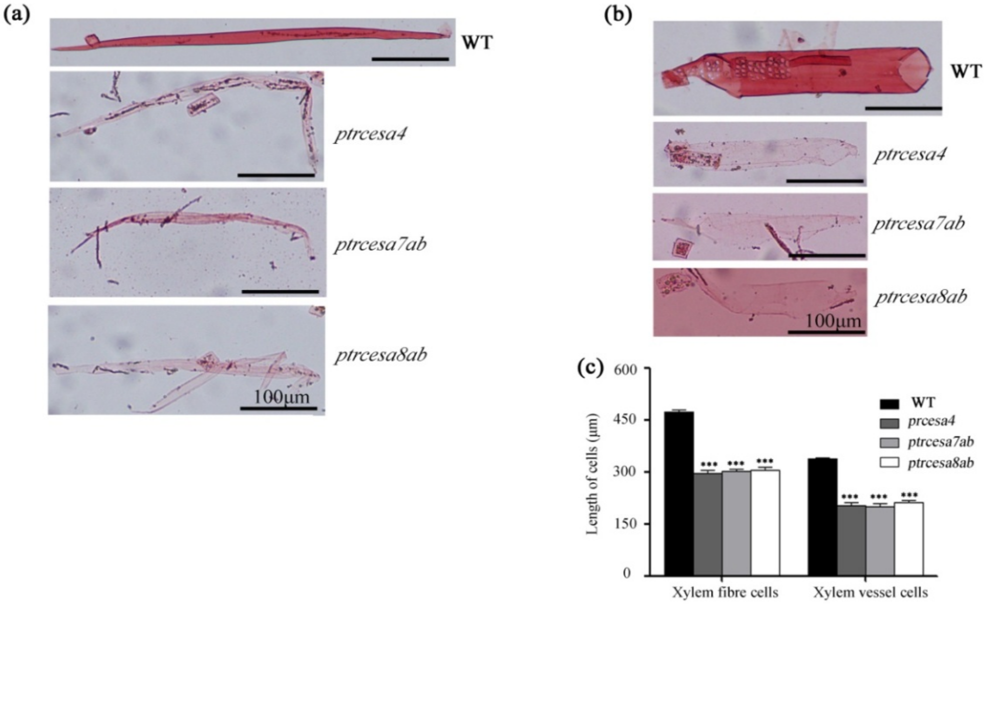

Fig. S13 Induction of tension wood (TW) in WT and *ptrcesa* mutants under gravi-stimulation. (a-f) WT, *ptrcesa8a*, *ptrcesa8b*, *ptrces8ab*, *ptrces7ab*, and *ptrcesa4* mutants straight grown for 4 months in a greenhouse were inclined by a 45˚ angle from the vertical direction to induce TW for 10 days. Scanning electron microscopy images were taken from the cross-sections of the 16th internode of each sample. Scale bars: 1mm.

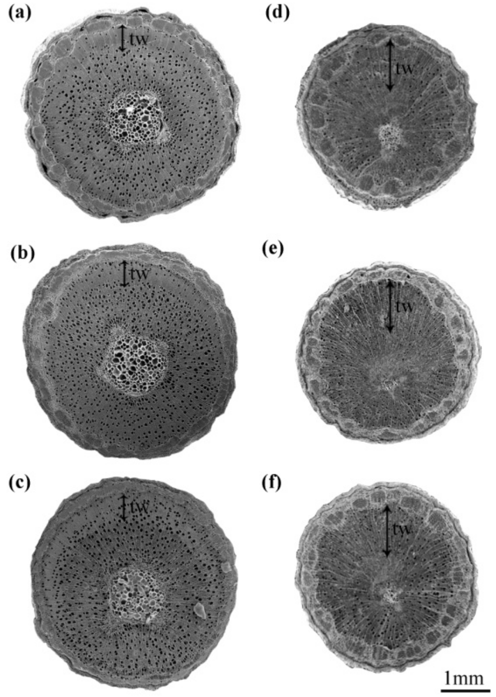

Fig. S14 Immunolocalization of crystalline cellulose in tension and opposite wood (TW, OW) sides in WT, *ptrcesa4, 7ab*, and *8ab* mutants. The 8μm transverse cross-sections of the 16th internode from each sample were incubated with CBM3a-6×His protein and anti-His antibody. CBM3a binds crystalline cellulose in plant cell wall. Signals (red) were detected with Alexa Fluor 633 goat anti-rat IgG. Arrowheads indicate G-layers of TW fibers in the WT. Scale bars: 10 μm.

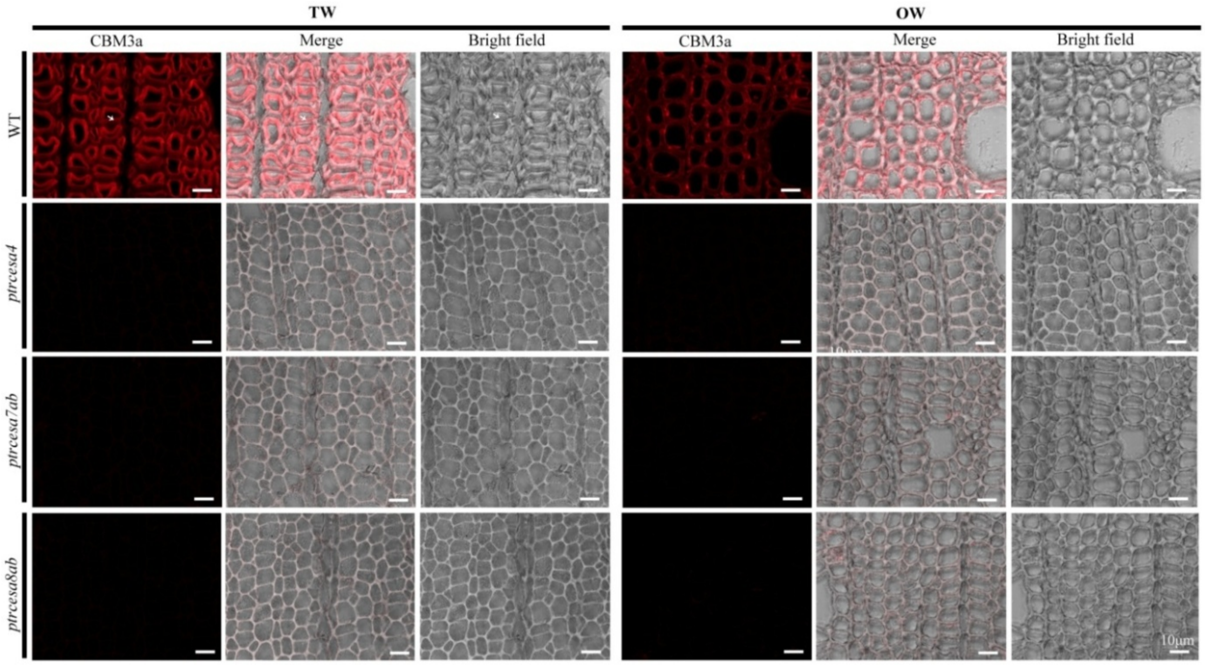

Fig. S15 Immunolocalization of the xylan in tension and opposite wood (TW, OW) sides in WT, *ptrcesa4, 7ab*, and *8ab* mutants. The 8μm transverse cross-sections of the 16th internode from each sample were incubated with the LM10 antibody. LM10 binds the xylan in plant cell wall. Signals (red) were detected with Alexa Fluor 633 goat anti-rat IgG. Scale bars: 10 μm.

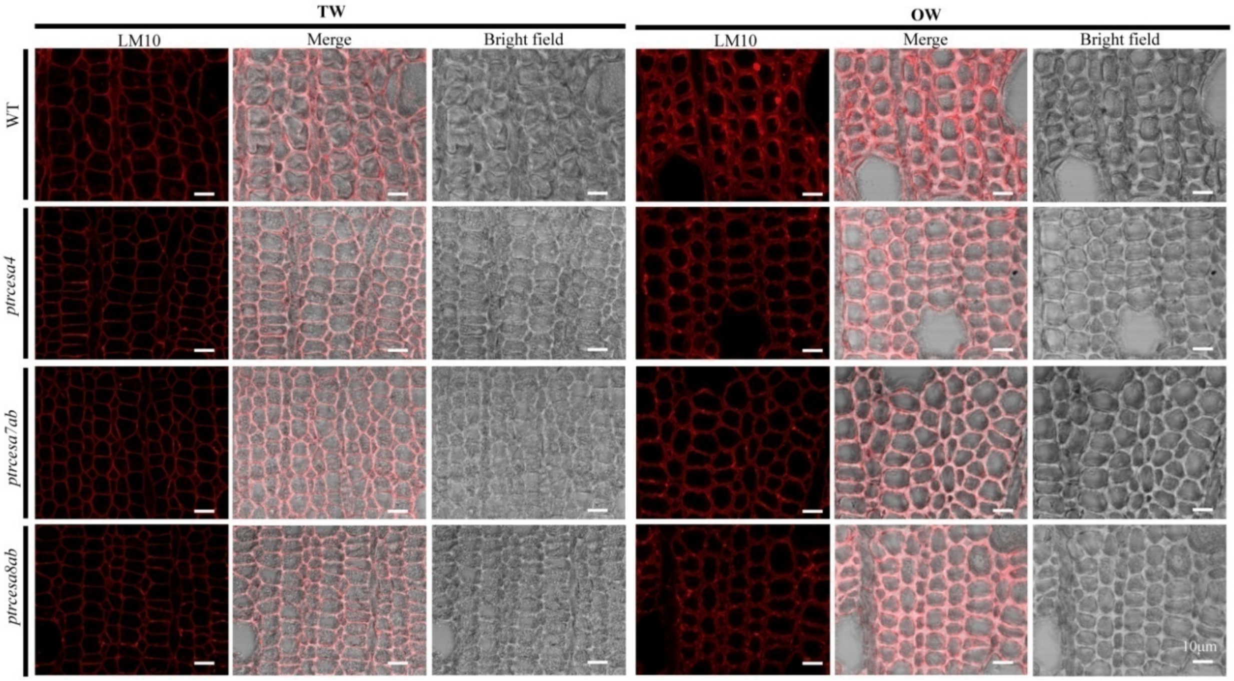

Fig. S16 Immunolocalization of β-(1→4)-galactan in tension and opposite wood (TW, OW) sides in WT, *ptrcesa4, 7ab*, and *8ab* mutants. The 8μm transverse cross-sections of the 16th internode from each sample were incubated with the LM5 antibody. LM5 bind β-(1→4)-galactan in plant cell wall. Signals (red) were detected with Alexa Fluor 633 goat anti-rat IgG. The fibres of OW side showed no fluorescence in WT and mutants. Arrowheads indicate G-layers. Scale bars: 10 μm.

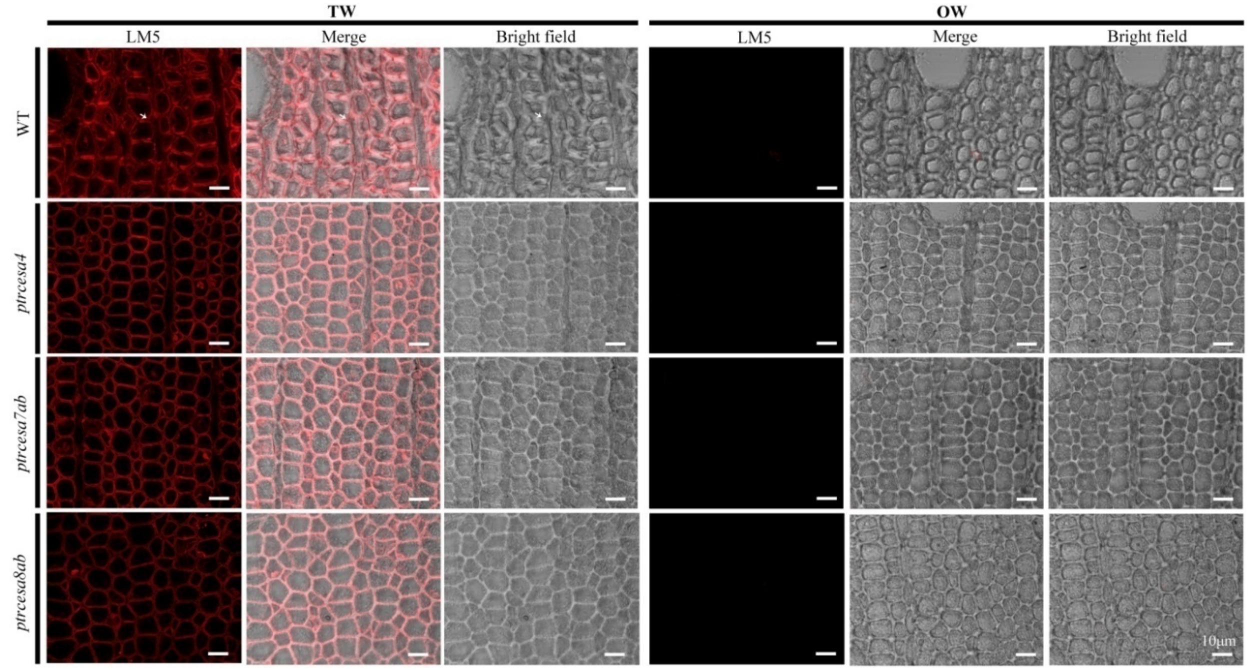

Fig. S17 Immunolocalization of the mannan in tension and opposite wood (TW, OW) sides in WT, *ptrcesa4, 7ab*, and *8ab* mutants. The 8μm transverse cross-sections of the 16th internode from each sample were incubated with the LM21 antibody. LM21 binds the mannan in plant cell wall. Signals (red) were detected with Alexa Fluor 633 goat anti-rat IgG. Scale bars: 10 μm.

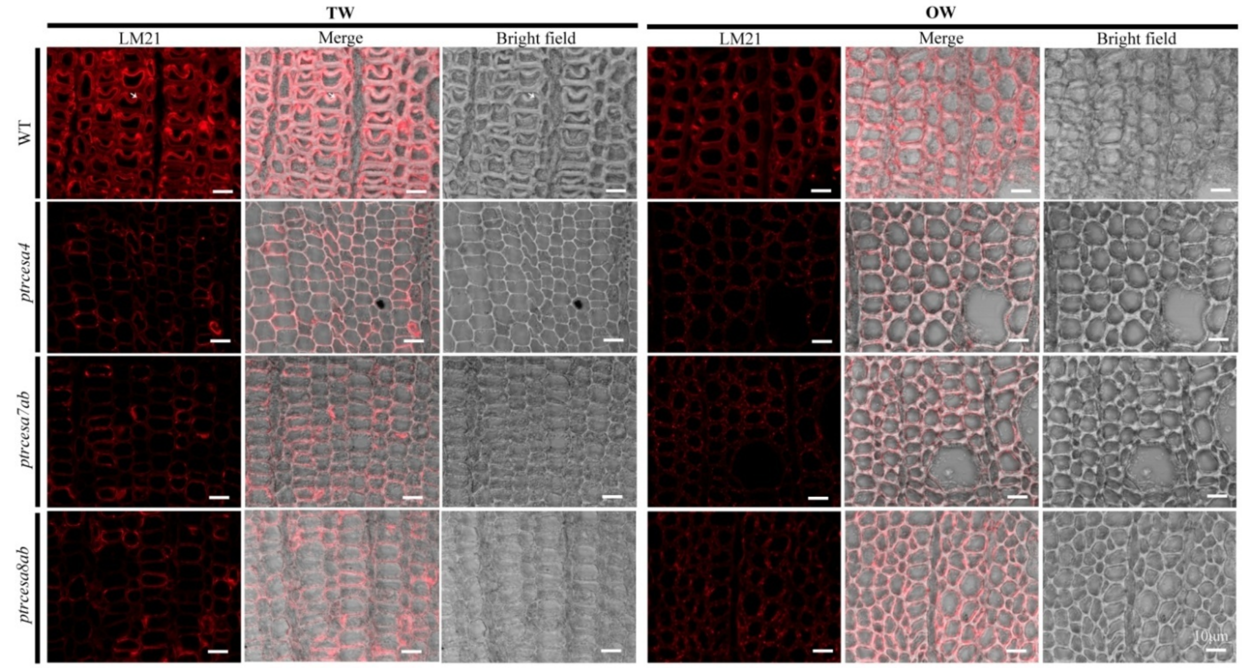

Fig. S18 Immunolocalization of crystalline cellulose in phloem fibres of WT, *ptrcesa4, 7ab*, and *8ab* mutants. Primary and secondary phloem fibers (PPF, SPF) in the 8μm transverse cross-sections of the 20th internode from each sample were incubated with CBM3a-6×His protein and anti-His antibody. Signals (red) were detected with Alexa Fluor 633 goat anti-rat IgG. CBM3a binds crystalline cellulose in plant cell wall. Scale bars: 10 μm.

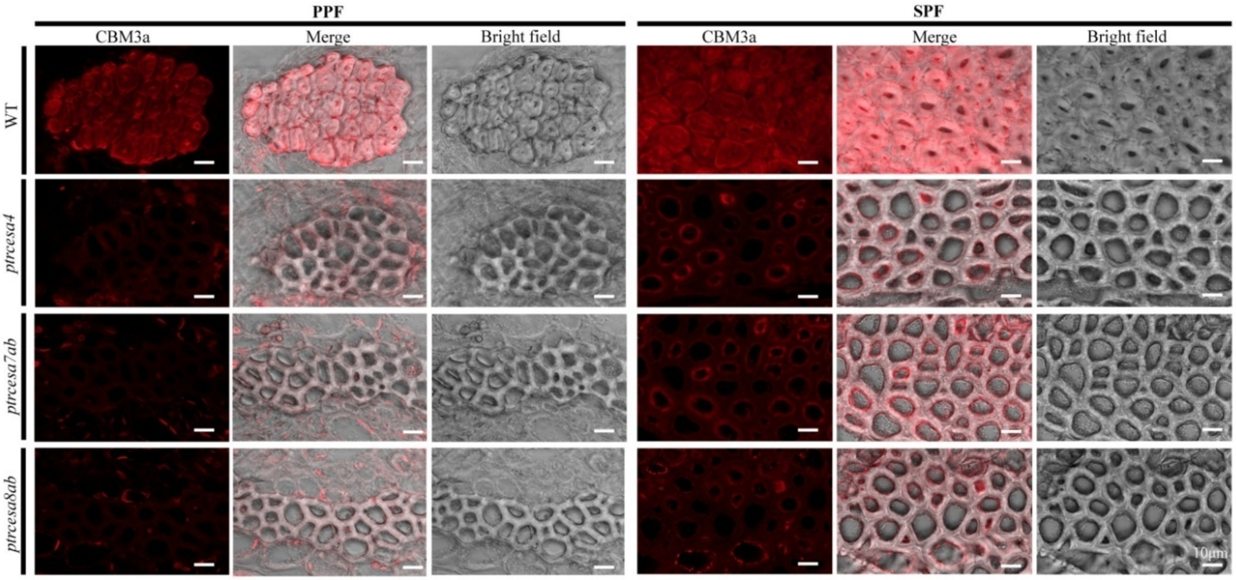

Fig. S19 Immunolocalization of β-(1→4)-galactan in phloem fibres of WT, *ptrcesa4, 7ab*, and *8ab* mutants. Primary and secondary phloem fibers (PPF, SPF) in the 8μm transverse cross-sections of the 20th internode from each sample were incubated with LM5 antibody. Signals (red) were detected with Alexa Fluor 633 goat anti-rat IgG. LM5 binds β-(1→4)-galactan in plant cell wall. Scale bars: 10 μm.

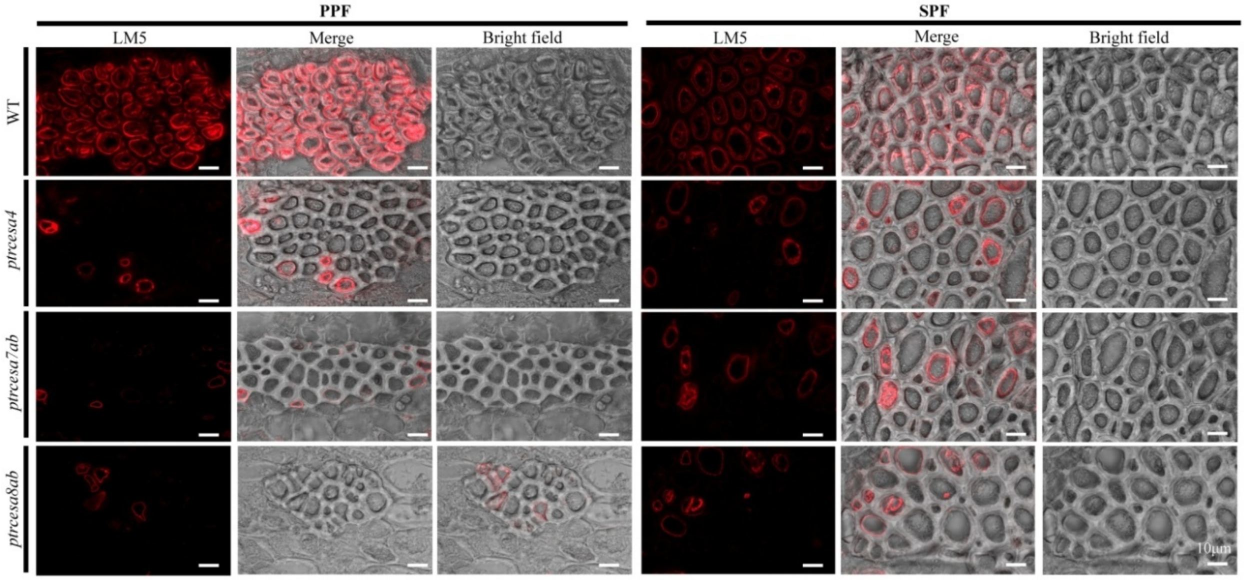

Fig. S20 Immunolocalization of the mannan in phloem fibres of WT, *ptrcesa4, 7ab*, and *8ab* mutants. Primary and secondary phloem fibers (PPF, SPF) in the 8 μm transverse cross-sections of the 20th internode from each sample were incubated with LM5 antibody. Signals (red) were detected with Alexa Fluor 633 goat anti-rat IgG. LM21 binds the mannan in the plant cell wall. Scale bars: 10 μm.

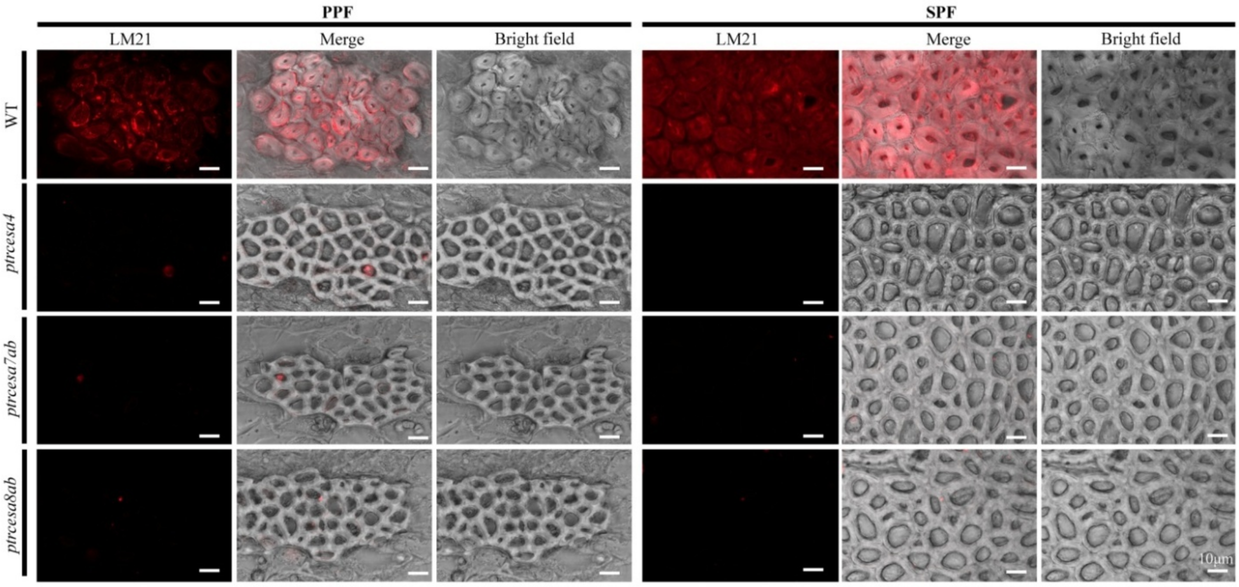

Fig. S21 Immunolocalization of the xylan in phloem fibres of WT, *ptrcesa4, 7ab*, and *8ab* mutants. Primary and secondary phloem fibers (PPF, SPF) in the 8 μm transverse cross-sections of the 20th internode from each sample were incubated with LM5 antibody. Signals (red) were detected with Alexa Fluor 633 goat anti-rat IgG. LM10 binds the xylan in plant cell wall. Scale bars: 10 μm.

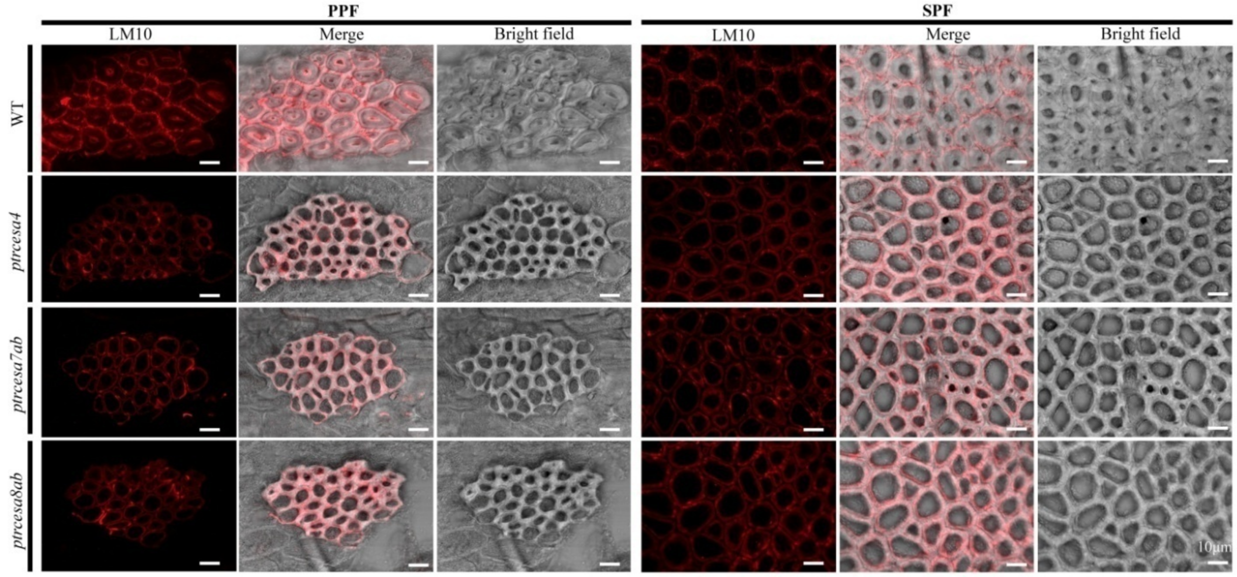

Fig. S22 Lignin phloroglucinol staining in WT, *ptrcesa4, 7ab*, and *8ab* mutants. (a-b) Cross-sections of the 10th stem internodes. (c) Cross-sections of the 8th leaf petioles (from apical bud) from WT and *ptrcesa* mutants. Scale bars: (a) 500 μm; (b) 200 μm; (c) 100 μm.

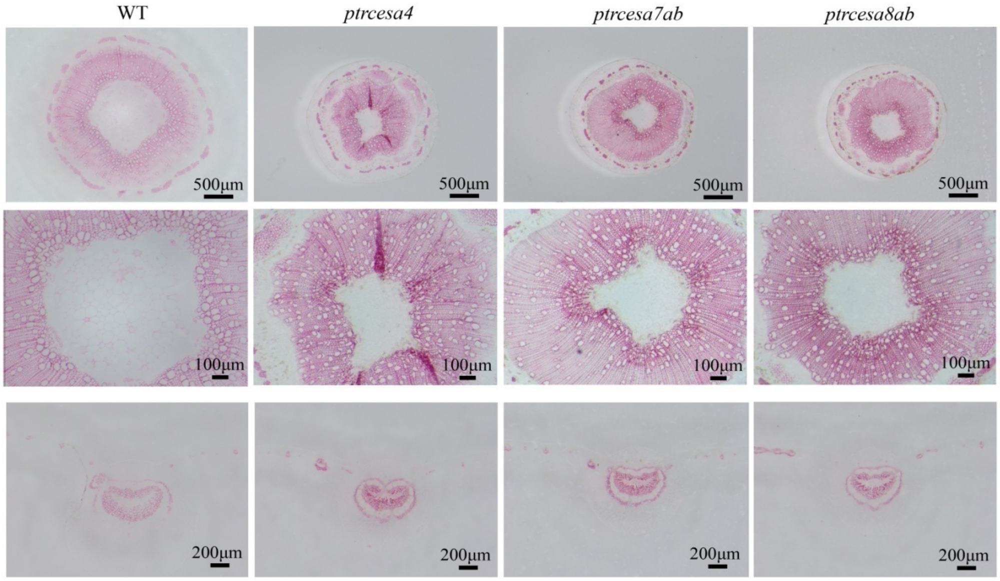

Fig. S23 Crystalline cellulose and lignin content in woods of WT, *ptrcesa7a*, *7b*, *8a*, and *8b* mutants. Values are means ± SD (n = 3). Asterisks denote significantly different from WT by Student's *t*-test: *$P < 0.05$, **$P < 0.01$, ***$P < 0.001$.

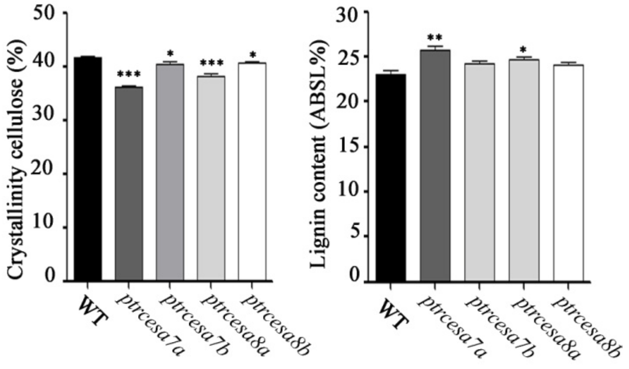

Methods S1 Analysis of putative Cas9/gRNA off-target sites. Putative gRNA off-target sites were identified by a BLASTN search using a target sequence in the *P. trichocarpa* genome. The final off-target sites were selected according to the following criteria: two to three mismatches in the PAM-proximal region, four to five mismatches within the PAM-proximal region, or one to two mismatches in the PAM. Specific primers (Table S1) flanking the off-target sites were used to amplify the corresponding loci, and the PCR amplicons were sequenced as described above. Eventually, 8 potential off-target sites for gCHLI1-a and gCHLI1-b were analyzed in 6 arbitrarily chosen *ptrchli1* mutants, and 25 potential off-target sites were evaluated in *ptrcesa4-1#, 4-2#* and *4-6#*, *ptrcesa7ab-3#, 7ab-7#* and *7ab-13#*, and *ptrcesa8ab-1#, 8ab-4#* and *8ab-8#*. These 9 knockout mutants were used for the subsequent phenotypic analysis.

Methods S2 RNA extraction and RT-PCR analysis. Total RNA was isolated using the pBIOZOL Plant Total RNA Extraction Reagent (Bio-Flux, China). First-strand cDNA synthesis was performed with 2 μg RNA using the PrimeScript RT Reagent Kit with gDNA Eraser (TaKaRa, China) according to the manufacturer's procedure. Reverse transcription PCR (RT-PCR) was employed to analyze the expression of *PtrCesA4*, *7A*, *7B*, *8A* and *8B* genes in the *ptrcesa* mutants. RT-PCR analysis was performed for three biological replicates each sample. The *PtrActin2* gene was used as a reference control. The PCR conditions were set as follows: 3 min at 95°C; 28 cycles of 30 s at 95°C, 30 s at 62°C and 30 s at 72°C; and 7 min at 72°C.

Methods S3 Scanning electron microscopy (SEM) of leaf epidermal cells. Fresh 6th leaves below the terminal buds of 3-month-old mutant and wild-type young trees were immediately frozen in liquid nitrogen and stored until observation. These samples were laid on a copper sheet (1.5 cm × 2 cm), transferred to an SEM chamber and scanned to produce micrographs. These images were used to determine the shapes and sizes of the epidermal cells.

Methods S4 Wood fibre and vessel cell length analysis. The lengths of the wood fibres and vessel cells were determined according to the previously described method (Lautner *et al.*, 2007) with minor modifications. The 20th stem internodes of 6-month-old wild-type and mutant trees were peeled, cut into small pieces, and incubated in a maceration solution (10% HNO_3 and 10% CrO_3 in v/v, 1:1) for 2-4 h at 60 °C. Thereafter, each stem internode was rinsed lightly with water, and the libriform fibres and vessel cells were disaggregated into the water by oscillation. After staining with 0.1% acid magenta, images were taken under a light microscope (Olympus, BX43). The lengths of the wood fibres and vessel cells were measured using ImageJ software.

References

Lautner S, Ehlting B, Windeisen E, Rennenberg H, Matyssek R, Fromm J. 2007. Calcium nutrition has a significant influence on wood formation in poplar. *New Phytologist* **173**: 743-752.

Table S1 Primers used in this study.

Purpose	Primer Name	Sequence (5'-3')
**Primers for CRISPR/Cas9 vector construction**		
**Cas9/gRNA-PtrCHLI1**	DT1-BsF/PtrCHLI1	ATATATGGTCTCGATTGTTACTTCCAGAAATTAAGGGTT
	DT1-F0/PtrCHLI1	TGTTACTTCCAGAAATTAAGGGTTTTAGAGCTAGAAATAGC
	DT2-R0/PtrCHLI1	AACGAAAGTCCACCACTGTTAGCAATCTCTTAGTCGACTCTAC
	DT2-BsR/PtrCHLI1	ATTATTGGTCTCTAAACGAAAGTCCACCACTGTTAGC
**Cas9/gRNA-PtrCHLI1/2-1**	DT1-BsF/PtrCHLI1/2-1	ATATATGGTCTCGATTGTTCTTGCTAAAGCTAATAGGTT
	DT1-F0/PtrCHLI1/2-1	TGTTCTTGCTAAAGCTAATAGGTTTTAGAGCTAGAAATAGC
	DT2-R0/PtrCHLI1/2-1	AACAAGATTGGAGGTGTCATGCAATCTCTTAGTCGACTCTAC
	DT2-BsR/PtrCHLI1/2-1	ATTATTGGTCTCTAAACAAGATTGGAGGTGTCATGC
**Cas9/gRNA-PtrCHLI1/2-2**	DT1-BsF/PtrCHLI1/2-2	ATATATGGTCTCGATTGTCGATGGGTATTGAAGTCAGTT
	DT1-F0/PtrCHLI1/2-2	TGTCGATGGGTATTGAAGTCAGTTTTAGAGCTAGAAATAGC
	DT2-R0/PtrCHLI1/2-2	AACTTTGCTGCTATAGTAGGGCCAATCTCTTAGTCGACTCTAC
	DT2-BsR/PtrCHLI1/2-2	ATTATTGGTCTCTAAACTTTGCTGCTATAGTAGGGCC
**Cas9/gRNA-PtrCesA4-1**	DT1-BsF/PtrCesA4-1	ATATATGGTCTCGATTGATACGATGCTTATGCATTGGTT
	DT1-F0/PtrCesA4-1	TGATACGATGCTTATGCATTGGTTTTAGAGCTAGAAATAGC
	DT2-R0/PtrCesA4-1	AACATTCGTCCGGCCTTTTCGTCAATCTCTTAGTCGACTCTAC
	DT2-BsR/PtrCesA4-1	ATTATTGGTCTCTAAACATTCGTCCGGCCTTTTCGTC
**Cas9/gRNA-PtrCesA4-2**	DT1-BsF/PtrCesA4-2	ATATATGGTCTCGATTGATCTGTGTTAGAACTTCGAGTT
	DT1-F0/PtrCesA4-2	TGATCTGTGTTAGAACTTCGAGTTTTAGAGCTAGAAATAGC
	DT2-R0/PtrCesA4-2	AACCCTCAAGGTCCTTGGAGGACAATCTCTTAGTCGACTCTAC
	DT2-BsR/PtrCesA4-2	ATTATTGGTCTCTAAACCCTCAAGGTCCTTGGAGGAC
**Cas9/gRNA-PtrCesA7A-1**	DT1-BsF/PtrCesA7A-1	ATATATGGTCTCGATTGTATTATTGCAGGTTAGACGGTT
	DT1-F0/PtrCesA7A-1	TGTATTATTGCAGGTTAGACGGTTTTAGAGCTAGAAATAGC
	DT2-R0/PtrCesA7A-1	AACTATTGGATCTCATGGAGAGCAATCTCTTAGTCGACTCTAC
	DT2-BsR/PtrCesA7A-1	ATTATTGGTCTCTAAACTATTGGATCTCATGGAGAGC
**Cas9/gRNA-PtrCesA7A-2**	DT1-BsF/PtrCesA7A-2	ATATATGGTCTCGATTGACAGGAATGGATAACGAGAGTT
	DT1-F0/PtrCesA7A-2	TGACAGGAATGGATAACGAGAGTTTTAGAGCTAGAAATAGC
	DT2-R0/PtrCesA7A-2	AACTGACACTGAAGGAAATGAGCAATCTCTTAGTCGACTCTAC
	DT2-BsR/PtrCesA7A-2	ATTATTGGTCTCTAAACTGACACTGAAGGAAATGAGC
**Cas9/gRNA-PtrCesA7B-1**	DT1-BsF/PtrCesA7B-1	ATATATGGTCTCGATTGCAAGATGACGATGCAGAAGGTT
	DT1-F0/PtrCesA7B-1	TGCAAGATGACGATGCAGAAGGTTTTAGAGCTAGAAATAGC
	DT2-R0/PtrCesA7B-1	AACTTGGATCTCATGGAGAACACAATCTCTTAGTCGACTCTAC
	DT2-BsR/PtrCesA7B-1	ATTATTGGTCTCTAAACTTGGATCTCATGGAGAACAC
**Cas9/gRNA-PtrCesA7B-2**	DT1-BsF/PtrCesA7B-2	ATATATGGTCTCGATTGCCCAATATGCTTGCTCCAGGTT
	DT1-F0/PtrCesA7B-2	TGCCCAATATGCTTGCTCCAGGTTTTAGAGCTAGAAATAGC
	DT2-R0/PtrCesA7B-2	AACGCAAGATCAATCCGTATAGCAATCTCTTAGTCGACTCTAC
	DT2-BsR/PtrCesA7B-2	ATTATTGGTCTCTAAACGCAAGATCAATCCGTATAGC
**Cas9/gRNA-PtrCesA8A-1**	DT1-BsF/PtrCesA8A-1	ATATATGGTCTCGATTGTGTTTCGAGTATGAGATCAGTT
	DT1-F0/PtrCesA8A-1	TGTGTTTCGAGTATGAGATCAGTTTTAGAGCTAGAAATAGC
	DT2-R0/PtrCesA8A-1	AACGGTGGGACATGATGCAAATCAATCTCTTAGTCGACTCTAC
	DT2-BsR/PtrCesA8A-1	ATTATTGGTCTCTAAACGGTGGGACATGATGCAAATC
**Cas9/gRNA-PtrCesA8A-2**	DT1-BsF/PtrCesA8A-2	ATATATGGTCTCGATTGGGAAATGAACTACCTCGACGTT
	DT1-F0/PtrCesA8A-2	TGGGAAATGAACTACCTCGACGTTTTAGAGCTAGAAATAGC
	DT2-R0/PtrCesA8A-2	AACGAGCTCGTGACATAGAGGGCAATCTCTTAGTCGACTCTAC

	DT2-BsR/PtrCesA8A-2	ATTATTGGTCTCTAAACGAGCTCGTGACATAGAGGGC
**Cas9/gRNA-PtrCesA8B-1**	DT1-BsF/PtrCesA8B-1	ATATATGGTCTCGATTGGGGCCGGAAAGTTTGCTTGGTT
	DT1-F0/PtrCesA8B-1	TGGGGCCGGAAAGTTTGCTTGGTTTTAGAGCTAGAAATAGC
	DT2-R0/PtrCesA8B-1	AACGAAAATCCCTTCCATTTGCCAATCTCTTAGTCGACTCTAC
	DT2-BsR/PtrCesA8B-1	ATTATTGGTCTCTAAACGAAAATCCCTTCCATTTGCC
**Cas9/gRNA-PtrCesA8B-2**	DT1-BsF/PtrCesA8B-2	ATATATGGTCTCGATTGGTGAGCCTTCTCAGCTTGCGTT
	DT1-F0/PtrCesA8B-2	TGGTGAGCCTTCTCAGCTTGCGTTTTAGAGCTAGAAATAGC
	DT2-R0/PtrCesA8B-2	AACGCTTTCTCCAGAGAACTTGCAATCTCTTAGTCGACTCTAC
	DT2-BsR/PtrCesA8B-2	ATTATTGGTCTCTAAACGCTTTCTCCAGAGAACTTGC
**Cas9/gRNA-PtrCesA7A/B-1**	DT1-BsF/PtrCesA7A/B-1	ATATATGGTCTCGATTGCGCAAAGAAAGAGGGAGGGGTT
	DT1-F0/PtrCesA7A/B-1	TGCGCAAAGAAAGAGGGAGGGGTTTTAGAGCTAGAAATAGC
	DT2-R0/PtrCesA7A/B-1	AACTATCCAGTTTCTGAACCTGCAATCTCTTAGTCGACTCTAC
	DT2-BsR/PtrCesA7A/B-1	ATTATTGGTCTCTAAACTATCCAGTTTCTGAACCTGC
**Cas9/gRNA-PtrCesA7A/B-2**	DT1-BsF/PtrCesA7A/B-2	ATATATGGTCTCGATTGTTTGTGACTTCAACTTTAAGTT
	DT1-F0/PtrCesA7A/B-2	TGTTTGTGACTTCAACTTTAAGTTTTAGAGCTAGAAATAGC
	DT2-R0/PtrCesA7A/B-2	AACCCTTCCTCGAGTCCAGCGGCAATCTCTTAGTCGACTCTAC
	DT2-BsR/PtrCesA7A/B-2	ATTATTGGTCTCTAAACCCTTCCTCGAGTCCAGCGGC
**Cas9/gRNA-PtrCesA8A/B-1**	DT1-BsF/PtrCesA8A/B-1	ATATATGGTCTCGATTGGTTCCGCCGGAACAGCAGAGTT
	DT1-F0/PtrCesA8A/B-1	TGGTTCCGCCGGAACAGCAGAGTTTTAGAGCTAGAAATAGC
	DT2-R0/PtrCesA8A/B-1	AACCTACAGAATAACAAATCCTCAATCTCTTAGTCGACTCTAC
	DT2-BsR/PtrCesA8A/B-1	ATTATTGGTCTCTAAACCTACAGAATAACAAATCCTC
**Cas9/gRNA-PtrCesA8A/B-2**	DT1-BsF/PtrCesA8A/B-2	ATATATGGTCTCGATTGAACTGGTTGTGTTTTCAATGTT
	DT1-F0/PtrCesA8A/B-2	TGAACTGGTTGTGTTTTCAATGTTTTAGAGCTAGAAATAGC
	DT2-R0/PtrCesA8A/B-2	AACCAAGTAGGTCGAGATGTATCAATCTCTTAGTCGACTCTAC
	DT2-BsR/PtrCesA8A/B-2	ATTATTGGTCTCTAAACCAAGTAGGTCGAGATGTATC
**Primers used to identify Cas9-gRNAs targeted sites**		
**gCHLI1-a/b-identify**	gCHLI1-a/b-identify-F	GCCACTAAACAATGCATCATTTC
	gCHLI1-a/b-identify-R	TCCTAGCTGAGGAAATTTGTTGC
**gCHLI1/2-a/b/c/d-identify**	gCHLI1/2-a/b/c/d-identify-F	TCGACCCTTCTCCAAACCTTCCA
	gCHLI1/2-a/b/c/d-identify-R	CCCTAGCTGAGGCAATTTGTTGC
**gCesA4-1/2-identify**	gCesA4-1/2-identify-F	ATGGCTGGCCTTGTCACGGGCAG
	gCesA4-1/2-identify-R	TGTCGACAGGATAATCAACGGAT
**gCesA4-3/4-identify**	gCesA4-3/4-identify-F	GGTGAGTTGAAAGATTACTGAAT
	gCesA4-3/4-identify-R	CTAGCACTCCACCCCACATTGTT
**gCesA7A-a/b-identify**	gCesA7A-a/b-identify-F	TCATTCTCTTCGATCTTATCTTT
	gCesA7A-a/b-identify-R	GGTTCTACACTAACTACAAACAA
**gCesA7A-c/d-identify**	gCesA7A-c/d-identify-F	TTGTTTGTAGTTAGTGTAGAACC
	gCesA7A-c/d-identify-R	ACTTCTAAGCAACAAGCAAACTT
**gCesA7B-a/b/c/d-identify**	gCesA7B-a/b/c/d-identify-F	GCATGGGATTCTCAAGCTGATTT
	gCesA7B-a/b/c/d-identify-R	TATAGATGTCGATGCGAATTACG
**gCesA8A-a/b-identify**	gCesA8A-a/b-identify-F	ATGATGGAATCTGGGGCTCCT
	gCesA8A-a/b-identify-R	TGCATCATTAAACCATTAACATT
**gCesA8A-c/d-identify**	gCesA8A-c/d-identify-F	TCAAGAACATTAAGGTCTTGGGT
	gCesA8A-c/d-identify-R	TATGATGATGCCAAGCCTTACTG
**gCesA8B-a/b-identify**	gCesA8B-a/b-identify-F	ATGATGGAATCTGGGGCTCCT
	gCesA8B-a/b-identify-R	AAACTTCATGGATTAAACTAGAA
**gCesA8B-c/d-identify**	gCesA8B-c/d-identify-F	CTATAGGAAGCAAAAAATCTCAT

	gCesA8B-c/d-identify-R	GGACTGCTAATAAATATTGAAGT
**gCesA7A-1/2-identify**	gCesA7A-1/2-identify-F	TCATTCTCTTCGATCTTATCTTT
	gCesA7A-1/2-identify-R	GGTTCTACACTAACTACAAACAA
**gCesA7A-3/4-identify**	gCesA7A-3/4-identify-F	CTGGACTAAAACAAACCCATGCT
	gCesA7A-3/4-identify-R	ACTTCTAAGCAACAAGCAAACTT
**gCesA7B-1/2-identify**	gCesA7B-1/2-identify-F	GCATGGGATTCTCAAGCTGATTT
	gCesA7B-1/2-identify-R	TATAGATGTCGATGCGAATTACG
**gCesA7B-3/4-identify**	gCesA7B-3/4-identify-F	CTGGCTTACCCCGTGTGTATGCT
	gCesA7B-3/4-identify-R	AAATACATTTCAGAGCTACTGGA
**gCesA8A-1/2-identify**	gCesA8A-1/2-identify-F	AATGTTAATGGTTTAATGATGCA
	gCesA8A-1/2-identify-R	TTATAATGCAGCATAGTGAATAT
**gCesA8A-3/4-identify**	gCesA8A-3/4-identify-F	TCAAGAACATTAAGGTCTTGGGT
	gCesA8A-3/4-identify-R	AAATGTCATGGAAGCCCACATTG
**gCesA8B-1/2-identify**	gCesA8B-1/2-identify-F	TTCTAGTTTAATCCATGAAGTTT
	gCesA8B-1/2-identify-R	ATGAGATTTTTTGCTTCCTATAG
**gCesA8B-3/4-identify**	gCesA8B-3/4-identify-F	ACTTCAATATTTATTAGCAGTCC
	gCesA8B-3/4-identify-R	AATGGGTAAAATGGGTAGCCTTG
**Primers used to identify off-targets**		
**gCHLI1a-off-1**	gCHLI1-a-off-1-F	TCGACCCTTCTCCAAACCTTCCA
	gCHLI1-a-off-1-R	CCCTAGCTGAGGCAATTTGTTGC
**gCHLI1-a-off-2**	gCHLI1-a-off-2-F	CCACATTCTTAAGCATATTAAA
	gCHLI1-a-off-2-R	TCACTTATATTCCTATCTACTC
**gCHLI1-a-off-3**	gCHLI1-a-off-3-F	TTGTTCTAAGCGCGACCGTAC
	gCHLI1-a-off-3-R	CAGAACTGAAGTGAAAAGATGAG
**gCHLI1-a-off-4**	gCHLI1-a-off-4-F	GCTTAGCTAGATGAATCGTTGT
	gCHLI1-a-off-4-R	CATTGCAAATCTTGGAGACATG
**gCHLI1-a-off-5**	gCHLI1-a-off-5-F	GCTGTACTCTTCATCGGAAGT
	gCHLI1-a-off-5-R	ATACTGTGTTTTCAACTTGATCC
**gCHLI1-b-off-1**	gCHLI1-b-off-1-F	TCGACCCTTCTCCAAACCTTCCA
	gCHLI1-b-off-1-R	CCCTAGCTGAGGCAATTTGTTGC
**gCHLI1-b-off-2**	gCHLI1-b-off-2-F	CCGTTCCTACGATATTTATCGG
	gCHLI1-b-off-2-R	CTCACGAACATGATACACTGAT
**gCHLI1b-off-3**	gCHLI1-b-off-3-F	GGCTAACGTCTCCTTCTGCA
	gCHLI1-b-off-3-R	GTTCCTCTCTATATCCACACG
**gCesA4-1-off-1**	gCesA4-1-off-1-F	GAACCTGAGCTCTGTGCACTA
	gCesA4-1-off-1-R	GAGCACAATGTCAACAAGTCTC
**gCesA4-1-off-2**	gCesA4-1-off-2-F	CTTGTATTGATCCCATGGTTGT
	gCesA4-1-off-2-R	AAGAAGTTACATCTTCGCAAGG
**gCesA4-3-off-1**	gCesA4-3-off-1-F	GCAGGCAAGGCTTGATGATATTG
	gCesA4-3-off-1-R	CCGGATTCTACAATCTTCATGTG
**gCesA4-3-off-2**	gCesA4-3-off-2-F	GTGTTTAGCATTGTGATAGCGG
	gCesA4-3-off-2-R	CAAGGTCTCTCGTTGAGAATTTC
**gCesA4-4-off-1**	gCesA4-4-off-1-F	GTAATCATGTAGAGTGCAATGG
	gCesA4-4-off-1-R	GCTTGACACACGTAGAACATC
**gCesA4-4-off-2**	gCesA4-4-off-2-F	GAACTTCTCGGCAAACCAAG
	gCesA4-4-off-2-R	GCAAGTGGAAGTGACAGGTT
**gCesA7A/B-1-off-1**	gCesA7A/B-1-off-1-F	GGATTGAGCAATGAGATTTC

	gCesA7A/B-1-off-1-R	TGTAAGTGTCATCCACTAGC
**gCesA7A/B-1-off-2**	gCesA7A/B-1-off-2-F	CCTTCTTCTCAACAGCGATT
	gCesA7A/B-1-off-2-R	AGACGATGGAGGAGATGCTT
**gCesA7A/B-1-off-3**	gCesA7A/B-1-off-3-F	GGTCGTGTCAGTTAGGCTGG
	gCesA7A/B-1-off-3-R	CAAGGCCAAATGTATTGCAATC
**gCesA7A/B-1-off-4**	gCesA7A/B-1-off-4-F	GCTTGCATCCTCTGGCTATA
	gCesA7A/B-1-off-4-R	CAATTAGGCGTTCATCTCCT
**gCesA7A/B-1-off-5**	gCesA7A/B-1-off-5-F	GTACCTTGATTTGAATCTGCA
	gCesA7A/B-1-off-5-R	TCCAAGAAATTCCTTGAGTGC
**gCesA7A/B-1-off-6**	gCesA7A/B-1-off-6-F	GCTGTCGATCTCTCAATCAAC
	gCesA7A/B-1-off-6-R	GTCATAGCTGCTGCTGATTCT
**gCesA7A/B-2-off-1**	gCesA7A/B-2-off-1-F	GATATGCCTCATTTCAATCCATG
	gCesA7A/B-2-off-1-R	CTAATAGGCCTTGTTGACATCAC
**gCesA7A/B-2-off-2**	gCesA7A/B-2-off-2-F	GCAAACATTATGGTGCACGAC
	gCesA7A/B-2-off-2-R	GCTGCCTTTGGTCATAACAAT
**gCesA7A/B-3-off-1**	gCesA7A/B-3-off-1-F	GAAAGTACAACTCCTTGCTA
	gCesA7A/B-3-off-1-R	CTTGTTGCTGCTTCATTCCT
**gCesA7A/B-3-off-2**	gCesA7A/B-3-off-2-F	GTTCATCATGCCAAATGAGGTA
	gCesA7A/B-3-off-2-R	TGCACAGTGAATTTGAAATTACC
**gCesA7A/B-3-off-3**	gCesA7A/B-3-off-3-F	GTTCATCATGCCAATTGAGGTA
	gCesA7A/B-3-off-3-R	GCATAATCTAGAGACCAAGAAC
**gCesA7A/B-3-off-4**	gCesA7A/B-3-off-4-F	ATAGCTATCCGTTTGCCTGTGA
	gCesA7A/B-3-off-4-R	GAGAATTCTAAGGTATTTAGAC
**gCesA7A/B-4-off-1**	gCesA7A/B-4-off-1-F	CATGCTAACACAAGAAGAAGG
	gCesA7A/B-4-off-1-R	CATCAACACGAAGGCTGTTG
**gCesA8A/B-2-off-1**	gCesA8A/B-2-off-1-F	CTAGGTGCTAACTACACTCA
	gCesA8A/B-2-off-1-R	GACGGTTCTCCTTCATTGTC
**gCesA8A/B-2-off-2**	gCesA8A/B-2-off-2-F	GAAGCCATTGGCTGTGATTG
	gCesA8A/B-2-off-2-R	CCGGTTTAGTCGCAACATAC
**gCesA8A/B-3-off-1**	gCesA8A/B-3-off-1-F	GCACGAAGATTCCAATTGGA
	gCesA8A/B-3-off-1-R	GTACAAGTAGTGGCAATGTGC
**gCesA8A/B-3-off-2**	gCesA8A/B-3-off-2-F	GAAGTTAGGACACATGTCAA
	gCesA8A/B-3-off-2-R	CTACCATGCTCTCGTTACGA
**gCesA8A/B-3-off-3**	gCesA8A/B-3-off-3-F	GATGTTCCTATGGACGTCTA
	gCesA8A/B-3-off-3-R	GATATCATTCCAATCAGAGCAA
**gCesA8A/B-3-off-4**	gCesA8A/B-3-off-4-F	GACTCTGGTTGATGTGAATACT
	gCesA8A/B-3-off-4-R	GCTACTAAGCTTCAACTATACA
**Primers used to identify transgenic plants**		
zCas	zCas-F	TGAGAACATCGTCATTGAGATGG
	zCas-R	TCAGCTTGTCATTCTCATCGTAC
Hyg	Hyg-F	GAGCTTGTCGATCGACAGAT
	Hyg-R	CATATGCGCGATTGCTGATC
PtrActin2	Actin2-F	AACATGGGATTGTTAGCAACTGG
	Actin2-R	TCCATCACCAGAATCCAGCACA
**Primers used for RT-PCR**		
**PtrCesA4**	PtrCesA4-F	GACGATGCCGAGTTTGGAGAGC
	PtrCesA4-R	CAACTGTGCAAAAGTGGGATAACCT

**PtrCesA7A**	PtrCesA7A-F	GGAATTCAGGGTCCGGTGTACG
	PtrCesA7A-R	AATAGACCTCCATCCACGGCAA
**PtrCesA7B**	PtrCesA7B -F	AAGGCAAGCTTTGTATGGCTATGAC
	PtrCesA7B -R	AATAGACCTCCAGCCACGACAA
**PtrCesA8A**	PtrCesA8A -F	TTGTTGGTGTTGTTGCGGGATT
	PtrCesA8A -R	CATCTCGCAGTTCATGTAACTCAACTACT
**PtrCesA8B**	PtrCesA8B-F	CTGGCATCGATACGAACTTCAC
	PtrCesA8B-R	TCTTGAGAGAAACTACAACGAGGA

Table S2 The Cas9/gRNA-induced target site mutations in single *PtrCHLI1* gene of *Populus trichocarpa*.

Gene: Potri.004G053400 (*PtrCHLI1*)

Vector: Cas9/gRNA-CHLI1
gRNA: gCHLI1-a (TTTACTTCCAGAAATTAAGGTGG); gCHLI1-b (CCGGAAAGTCCACCACTGTTAGG)
Target sequences: CHLI1-a,TTTACTTCCAGAAATTAAGGTGG; CHLI1-b, CCGGAAAGTCCACCACTGTTAGG

**Line (#)**	**CHLI1-a**<br>TTTACTTCCAGAAATTAAGGTGG	**Mutation types**<br>(Number of plasmids containing the cloned PCR products)		**CHLI1-b**<br>CCGGAAAGTCCACCACTGTTAGG	**Mutation types**<br>(Number of plasmids containing the cloned PCR products)	
ptrchli1-1#	... - - - - - - - - - - - - - - - - - AAGGTGGTTGCT	-44 (×25)	homozygous	AGGGACCGGAA - …	-44 (×25)	homozygous
ptrchli1-2#	GTTGATTTACTTCCAGAAATT - AGGTGGTTGCT<br>GTTGATTTACTTCCAGAAATTAAGGTGGTTGCT	-1 (×16)<br>0 (×9)	heterozygous	AGGGACC - - - - - - - - CACCACTGTTAGGTCCAT<br>AGGGACCGGAAAGTCCACCACTGTTAGGTCCAT	-8 (×16)<br>0 (×9)	heterozygous
ptrchli1-3#	GTTGATTTACTTCCAGAAATTAAGGTGGTTGCT<br>… - AGGTGGTTGCT<br>… - AAGGTGGTTGCT<br>… -AGGTGGTTGCT	0 (×13)<br>-55 (×7)<br>-44 (×2)<br>-42 (×3)	chimeric	AGGGACCGGAA a AGTCCACCACTGTTAGGTCCAT<br>A - …<br>AGGGACCGGAA - …<br>AGGGACCGGAAAT - - - - - - - - - - - - - - - - - - - …	+1 (×13)<br>-55 (×7)<br>-44 (×2)<br>-42 (×3)	chimeric
ptrchli1-4#	... - - - - - - - - - - - - - - - -TGACCCCTATAACTCAG<br>- AGGTGGTTGCT	-66 (×11)<br>-22 (×14)	biallelic	AGG- …<br>AGGGACCGGAA - GTCCACCACTGTTAGGTCCAT	-66 (×11)<br>-1 (×14)	biallelic
ptrchli1-5#	… - GGTGACCCCTATA<br>… - GGTGACCCCTATA	-57 (×17)<br>-31 (×8)	biallelic	AGGGACCGGA - …<br>AGGGACCGGAA a AGTCCACCACTGTTAGGTCCAT	-57 (×17)<br>+1 (×8)	biallelic
ptrchli1-6#	… - AAGGTGGTTGCT<br>GTTGATTTACTTCCAGAAATTAAGGTGGTTGCT	-44 (×10)<br>0 (×15)	heterozygous	AGGGACCGGAA - …<br>AGGGACCGGAAAGTCCACCACTGTTAGGTCCAT	-44 (×10)<br>0 (×15)	heterozygous
ptrchli1-7#	GTTGATTTACTTCCAGAAATT - AGGTGGTTGCT<br>… - AAGGTGGTTGCT	-1 (×14)<br>-44 (×11)	biallelic	AGGGACCGGAA - GTCCACCACTGTTAGGTCCAT<br>AGGGACCGGAA - …	-1 (×14)<br>-44 (×11)	biallelic
ptrchli1-8#	… - AAGGTGGTTGCT<br>GTTGATTTACTTCCAGAAATTAAGGTGGTTGCT	-44 (×16)<br>0 (×9)	heterozygous	AGGGACCGGAA - …<br>AGGGACCGGAAAGTCCACCACTGTTAGGTCCAT	-44 (×16)<br>0 (×9)	heterozygous
ptrchli1-9#	… - AAGGTGGTTGCT<br>GTTGATTTACTTCCAGAAATTAAGGTGGTTGCT	-44 (×10)<br>0 (×15)	heterozygous	AGGGACCGGAA - …<br>AGGGACCGGAAAGTCCACCACTGTTAGGTCCAT	-44 (×10)<br>0 (×15)	heterozygous
ptrchli1-10#	GTTGATTTACTTCCAGAAATT - AGGTGGTTGCT	-1 (×25)	homozygous	AGGGACCGGAA - GTCCACCACTGTTAGGTCCAT<br>AGGGACCGG - - - - - - - - - - - - - - - - AGGTCCAT	-1 (×13)<br>-16 (×12)	biallelic
ptrchli1-11#	… - AGGTGGTTGCT<br>… - - - - - - - - - - - - - - - - - - - GATCCAGAGTCGA	-45 (×8)<br>-83 (×17)	biallelic	AGGGACCGGAA - …<br>AGGGACCGGAA - …	-45 (×8)<br>-83 (×17)	biallelic
ptrchli1-12#	… - AGGTGGTTGCT<br>GTTGATTTAT - - - - - - - - - - - - AGGTGGTTGCT	-45 (×15)<br>-12 (×10)	biallelic	AGGGACCGGAA - …<br>AGGGACCGGAA - GTCCACCACTGTTAGGTCCAT	-45 (×15)<br>-1 (×10)	biallelic
ptrchli1-13#	… - AGGTGGTTGCT<br>… - AAGGTGGTTGCT	-45 (×3)<br>-44 (×12)	chimeric	AGGGACCGGAA - …<br>AGGGACCGGAA - …	-45 (×3)<br>-44 (×12)	chimeric

	… -AGGTGGTTGCT<br>GTTGATTTACTTCCAGAAATTAAGGTGGTTGCT	-42 (×6)<br>0 (×4)		AGGGACCGGAAATT - - - - - - - - - - - - - - - - - - - …<br>AGGGACCGGAAAGTCCACCACTGTTAGGTCCAT	-42 (×6)<br>0 (×4)	
ptrchli1-14#	… - - - - - - - - - - - - - - - - - - - GATCCAGAGTCGA	-83 (×25)	homozygous	AGGGACCGGAA - …	-83 (×25)	homozygous
ptrchli1-15#	… - AGGTGGTTGCT	-45 (×25)	homozygous	AGGGACCGGAA - …	-45 (×25)	homozygous
ptrchli1-16#	GTTGATTTACTTCCAGAAATTA a<br>AGGTGGTTGCT<br>GTTGATTTACTTCCAGAAATT - AGGTGGTTGCT	+1 (×13)<br>-1 (×12)	biallelic	AGGGACCGGAA - GTCCACCACTGTTAGGTCCAT<br>AGGGACCGGAA a AGTCCACCACTGTTAGGTCCAT	-1 (×13)<br>+1 (×12)	biallelic
ptrchli1-17#	… - - - - - - - - - - - - - - - - - -<br>CCTATAACTCAGATGA<br>GTTGATTTACTTCCAGAAATTAAGGTGGTTGCT	-67 (×15)<br>0 (×10)	heterozygous	AGGGACC - …<br>AGGGACCGGAAAGTCCACCACTGTTAGGTCCAT	-67 (×15)<br>0 (×10)	heterozygous
ptrchli1-18#	... - - - - - - - - - - - - - - - -TGACCCCTATAACTCAG<br>- AGGTGGTTGCT	-66 (×12)<br>-22 (×13)	biallelic	AGG- …<br>AGGGACCGGAA - GTCCACCACTGTTAGGTCCAT	-66 (×12)<br>-1 (×13)	biallelic

Table S3 The Cas9/gRNA-targeted mutations in both *PtrCHLI1* and *2* genes of *Populus trichocarpa*.

Gene: Potri.004G053400 (*PtrCHLI1*) / Potri.011G063400 (*PtrCHLI2*)

Vector: Cas9/gRNA-CHLI1/2-1
gRNA: gCHLI1/2-a (CTTCTTGCTAAAGCTAATAGAGG); gCHLI1/2-b (CCCAAGATTGGAGGTGTCATGAT)
Target sequences: CHLI1-a, CTTCTTGCTAAAGCTAATAGAGG; CHLI1-b, CCCAAGATTGGAGGTGTCATGAT
CHLI2-a, CTTCTTGCTAAAGCTAATAGAGG; CHLI2-b, CCCAAGATTGGAGGTGTCATGAT

**Line (#)**	**CHLI1/2-a**<br>CHLI1, CTTCTTGCTAAAGCTAATAGAGG<br>CHLI2, CTTCTTGCTAAAGCTAATAGAGG	**Mutation types** (Number of plasmids containing the cloned PCR products)		**CHLI1/2-b**<br>CHLI1, CCCAAGATTGGAGGTGTCATGAT<br>CHLI2, CCCAAGATTGGAGGTGTCATGAT	**Mutation types** (Number of plasmids containing the cloned PCR products)	
***ptrchli1/2-1#*-CHLI1**	... - TAGAGGCATTC	-319 (×25)	homozygous	TTGATCCCAAGA - - - - - - - - - - - - - - - - - - - …	-319 (×25)	homozygous
**-CHLI2**	CGGGTCTTCTTGCTAAAGC - - - TAGAGGGATTC<br>CGGGTCTTCTTGCTAAAGCTA - TAGAGGGATTC	-3 (×16)<br>-1 (×9)	biallelic	TTGATCCC - - - - - - - GAGGTGTCATGATCATGG<br>TTGATCCCAAG - TTGGAGGTGTCATGATCATGG	-7 (×16)<br>-1 (×9)	biallelic
***ptrchli1/2-2#*-CHLI1**	CGGGTCTTCTTGCTAAAGC - - - TAGAGGCATTC	-3 (×25)	homozygous	TTGATCCC - - - - - - - GAGGTGTCATGATCATGG<br>TTGAT - - - - - - - - - - - - - - - GTCATGATCATGG	-7 (×13)<br>-15 (×12)	biallelic
**-CHLI2**	TTGTGGGACAATT - - - - - - ... TCGTCATTCTTAC<br>CGGGTCTTCTTGC - - - - - - - - - TAGAGGGATTC	-356 (×19)<br>-9 (×6)	biallelic	TTGATCCCAAG a ATTGGAGGTGTCATGATCATGG<br>TTGATCCCAAG - - - - GAGGTGTCATGATCATGG	+1 (×19)<br>-4 (×6)	biallelic
***ptrchli1/2-3#*-CHLI1**	... - TAGAGGCATTC	-319 (×25)	homozygous	TTGATCCCAAGA - - - - - - - - - - - - - - - - - - - …	-319 (×25)	homozygous
**-CHLI2**	... - TAGAGGCATTC	-321 (×25)	homozygous	TTGATCCCAA - …	-321 (×25)	homozygous
***ptrchli1/2-4#*-CHLI1**	... - TAGAGGCATTC	-319 (×25)	homozygous	TTGATCCCAAGA - - - - - - - - - - - - - - - - - - - …	-319 (×25)	homozygous
**-CHLI2**	CGGGTCTTCTTGCTAAAGC - - - TAGAGGGATTC<br>CGGGTCTTCTTGCTAAAGCTA - TAGAGGGATTC<br>CGGGTCTTCTTGCTAAAGCTAATAGAGGGATTC	-3 (×14)<br>-1 (×7)<br>0 (×4)	chimeric	TTGATCCC - - - - - - - GAGGTGTCATGATCATGG<br>TTGATCCCAAG - TTGGAGGTGTCATGATCATGG<br>TTGATCCCAAGATTGGAGGTGTCATGATCATGG	-7 (×14)<br>-1 (×7)<br>0 (×4)	chimeric
***ptrchli1/2-5#*-CHLI1**<br>**-CHLI2**	... - TAGAGGCATTC<br>GCTCTCACCGAGG - - - - - - ... TTAATCTTTTGGA<br>CGGGTCTTCTTGCTAAAGCTAATAGAGGGATTC	-319 (×7)<br>-66 (×5)<br>0 (×13)	chimeric	TTGATCCCAAGA - - - - - - - - - - - - - - - - - - - …<br>TTGATCCCAAGATTGGAGGTGTCATGATCATGG	-319 (×7)<br>0 (×18)	heterozygous
	... - TAGAGGCATTC<br>CGGGTCTTCTTGCTAAAGCTAA a TAGAGGGATTC	-319 (×18)<br>+1 (×7)	biallelic	TTGATCCCAAGA - - - - - - - - - - - - - - - - - - - …<br>TTGATCCCAAGA - - - GAGGTGTCATGATCATGG	-319 (×18)<br>-3 (×7)	biallelic
***ptrchli1/2-6#*-CHLI1**<br>**-CHLI2**	... - TAGAGGCATTC<br>CGGGTCT - - - - - - - - - - - - - - - TAGAGGCATTC	-319 (×17)<br>-15 (×8)	biallelic	TTGATCCCAAGA - - - - - - - - - - - - - - - - - - - …<br>TTGATCCCAAG - TTGGAGGTGTCATGATCATGG	-319 (×17)<br>-1 (×8)	biallelic
	CGGGTCTTCTTGCTAAAGCTAA a TAGAGGGATTC<br>TGGTGACCCCTATAA ... - - - - - - - - - - - ^43 ATTC<br>(^43:aagatcaacataaagattaacttcatcaacatctcaccgaggg)	+1 (×15)<br>-217+43<br>(×10)	biallelic	TTGATCCCAAG - TTGGAGGTGTCATGATCATGG	-1 (×25)	homozygous
***ptrchli1/2-7#*-CHLI1**<br>**-CHLI2**	... - TAGAGGCATTC<br>CGGGTCT - - - - - - - - - - - - - - - TAGAGGCATTC	-319 (×16)<br>-15 (×9)	biallelic	TTGATCCCAAGA - - - - - - - - - - - - - - - - - - - …<br>TTGATCCCAAG - TTGGAGGTGTCATGATCATGG	-319 (×16)<br>-1 (×9)	biallelic
	CGGGTCTTCTTGCTAAAGCTAA a TAGAGGGATTC	+1 (×16)	heterozygous	TTGATCCCAAG - TTGGAGGTGTCATGATCATGG	-1 (×19)	biallelic

	CGGGTCTTCTTGCTAAAGCTAATAGAGGGATTC	0 (×9)		TTGATCC - - - - - TTGGAGGTGTCATGATCATGG	-5 (×6)	
***ptrchli1/2-8#*-CHLI1**	... - TAGAGGCATTC	-319 (×25)	homozygous	TTGATCCCAAGA - …	-319 (×25)	homozygous
**-CHLI2**	CGGGTCTTCTTGCTAAAGCTAA a TAGAGGGATTC<br>CGGGTCTTCTTGCTAAAGCTAATAGAGGGATTC	+1 (×5)<br>0 (×20)	heterozygous	TTGATCC - - - - - TTGGAGGTGTCATGATCATGG	-5 (×25)	homozygous
***ptrchli1/2-9#*-CHLI1**	... - TAGAGGCATTC	-319 (×25)	homozygous	TTGATCCCAAGA - …	-319 (×25)	homozygous
**-CHLI2**	CGGGTCTTCTTGCTAAAGCTAA a TAGAGGGATTC<br>CGGGTCTTCTTGCTAAAGC - - ATAGAGGGATTC	+1 (×13)<br>-2 (×12)	biallelic	TTGATCCCAAG - TTGGAGGTGTCATGATCATGG	-1 (×25)	homozygous
***ptrchli1/2-10#*-<br>CHLI1**	... - TAGAGGCATTC	-319 (×25)	homozygous	TTGATCCCAAGA - …	-319 (×25)	homozygous
**-CHLI2**	CGGGTCTTCTTGCTAAAGCTAA a TAGAGGGATTC<br>CGGGTCTTCTTGCTAAAGCTA - TAGAGGGATTC<br>CGGGTCTTCTTGCTAAAGCTAATAGAGGGATTC	+1 (×14)<br>-1 (×5)<br>0 (×6)	chimeric	TTGATCCCAAG - TTGGAGGTGTCATGATCATGG	-1 (×25)	homozygous
***ptrchli1/2-11#*-<br>CHLI1**	... - TAGAGGCATTC<br>CGGGTCT - - - - - - - - - - - - - - - TAGAGGCATTC	-319 (×9)<br>-15 (×16)	biallelic	TTGATCCCAAGA - …<br>TTGATCCCAAG - TTGGAGGTGTCATGATCATGG	-319 (×9)<br>-1 (×16)	biallelic
**-CHLI2**	CGGGTCTTCTTGCTAAAGCTAA a TAGAGGGATTC	+1 (×25)	homozygous	TTGATCCCAAG - TTGGAGGTGTCATGATCATGG	-1 (×25)	homozygous
***ptrchli1/2-12#*-<br>CHLI1**	... - TAGAGGCATTC	-319 (×25)	homozygous	TTGATCCCAAGA - …	-319 (×25)	homozygous
**-CHLI2**	CGGGTCTTCTTGCTAAAGCTAA a TAGAGGGATTC<br>CGGGTCTTCTTGCTAAAGCTA - TAGAGGGATTC	+1 (×14)<br>-1 (×11)	biallelic	TTGATCCCAAG - TTGGAGGTGTCATGATCATGG<br>TTGATCCCAAG a ATTGGAGGTGTCATGATCATGG	-1 (×14)<br>+1 (×11)	biallelic

Vector: Cas9/gRNA-CHLI1/2-2
gRNA: gCHLI1/2-c (GTCGATGGGTATTGAAGTCAGGG); gCHLI1/2-d (CCATTTGCTGCTATAGTAGGGCA)
Target sequences: CHLI1-c, GTCGATGGGTATTGAAGTCAGGG; CHLI1-d, CCATTTGCTGCTATAGTAGGGCA
CHLI2-c, GTCCATGGGTATTGCAGTCAGGG; CHLI2-d, CCATTTGCTGCAATAGTAGGGCA

**Line (#)**	**CHLI1/2-c**<br>CHLI1, GTCGATGGGTATTGAAGTCAGGG<br>CHLI2, GTCCATGGGTATTGAAGTCAGGG	**Mutation types**<br>(Number of plasmids containing the cloned PCR products)		**gCHLI1/2-d**<br>CHLI1, CCATTTGCTGCTATAGTAGGGCA<br>CHLI2, CCATTTGCTGCAATAGTAGGGCA	**Mutation types**<br>(Number of plasmids containing the cloned PCR products)	
***ptrchli1/2-13#*-<br>CHLI1**	CCAGAGTCGATGGGTATTGAA - TCAGGGAGAGT	-1 (×25)	homozygous	TATATCCATTTGCTGCTATAGTAGGGCAAGATG<br>TATATCCATT - GCTGCTATAGTAGGGCAAGATG	0 (×22)<br>-1 (×3)	heterozygous
**-CHLI2**	CCAGAGTCCATGGGTATTGA - - - CAGGGAGAGT<br>CCAGAGTCCATGGGTATTGA - - - - - - - - AGAGT	-3 (×15)<br>-8 (×10)	biallelic	TGTATCCATTTGCTGCAATAGTAGGGCAAGATG	0 (×25)	WT
***ptrchli1/2-14#*-<br>CHLI1**	CCAGAGTCGATGGGTATTGAA - TCAGGGAGAGT<br>... - - - - - - - - - - - - - - - - - - AAGTCAGGGAGAGT	-1 (×13)<br>-159 (×12)	biallelic	TATATCCATTTGCTGCTATAGTAGGGCAAGATG	0 (×25)	WT
**-CHLI2**	CCAGAGTCCATGGGTATTGAAGTCAGGGAGAGT	0 (×25)	WT	TGTATCCATTTGCTGCAATAGTAGGGCAAGATG	0 (×25)	WT
***ptrchli1/2-15#*-<br>CHLI1**	CCAGAGTCGATGGGTATTGAA - TCAGGGAGAGT<br>... - - - - - - - - - - - - - - - - - - AAGTCAGGGAGAGT	-1 (×9)<br>-159 (×16)	biallelic	TATATCCATTTGCTGCTATAGTAGGGCAAGATG	0 (×25)	WT
**-CHLI2**	- - - - - - - CCATGGGTATTGAAGTCAGGGAGAGT<br>CCAGAGTCCATGGGTATTGAAGTCAGGGAGAGT	-7 (×14)<br>0 (×11)	heterozygous	TGTATCCATTTGCTGCAATAGTAGGGCAAGATG	0 (×25)	WT
***ptrchli1/2-16#*-<br>CHLI1**	CCAGAGTCGATGGGTATTGAA - TCAGGGAGAGT<br>CCAGAGTCGATGGGTATTGAAGTCAGGGAGAGT	-1 (×21)<br>0 (×4)	heterozygous	TATATCCATTT - - TGCTATAGTAGGGCAAGATG<br>TATATCCATTTGCTGCTATAGTAGGGCAAGATG	-2 (×13)<br>0 (×12)	heterozygous

**-CHLI2**	CCAGAGTCC - - - - - - - - - TCTGTCAGGGAGAGT<br>CCAGAGTCCATGGGTATTGAAGTCAGGGAGAGT	-9 (×14)<br>0 (×11)	heterozygous	TGTATCCATTTGCTGCAATAGTAGGGCAAGATG	0 (×25)	WT
***ptrchli1/2-17#*-CHLI1 -CHLI2**	CCAGAGTCGATGGGTATTGA - - TCAGGGAGAGT<br>... - TCAGGGAGAGT	-2 (×9)<br>-210 (×16)	biallelic	TATATCCATT - GCTGCTATAGTAGGGCAAGATG<br>TATATCCATTTG - …	-1 (×9)<br>-210 (×16)	biallelic
	CCAGAGTCCATGGGTATT - - - - TCAGGGAGAGT<br>CCAGAGTCCATGGGTATTGAA - TCAGGGAGAGT<br>CCAGAGTCCATGGGTATTGA - - - - - - - - - - - GT	-4 (×18)<br>-1 (×5)<br>-11 (×2)	chimeric	TGTATCCATTTGCTGCAATAGTAGGGCAAGATG	0 (×25)	WT
***ptrchli1/2-18#*-CHLI1 -CHLI2**	... - TCAGGGAGAGT	-211 (×25)	homozygous	TATATCCATTT - …	-211 (×25)	homozygous
	CCAGAGTCCATGGGTATTGA - - TCAGGGAGAGT<br>- TCAGGGAGAGT	-2 (×13)<br>-24 (×12)	biallelic	TGTATCCATTTGCTGCAATAGTAGGGCAAGATG	0 (×25)	WT
***ptrchli1/2-19#*-CHLI1 -CHLI2**	... - TCAGGGAGAGT<br>CCAGAGTCGATGGGTATTGA - - TCAGGGAGAGT	-210 (×10)<br>-2 (×15)	biallelic	TATATCCATTTG - …<br>TATATCCATT - GCTGCTATAGTAGGGCAAGATG	-210 (×10)<br>-1 (×15)	biallelic
	CCAGAGTCCATGGGTATT - - - - TCAGGGAGAGT<br>CCAGAGTCCATGGGTATTGA - - - - - - - - - - - GT	-4 (×9)<br>-11 (×16)	biallelic	TGTATCCATTTGCTGCAATAGTAGGGCAAGATG	0 (×25)	WT
***ptrchli1/2-20#*-CHLI1 -CHLI2**	... - TCAGGGAGAGT<br>CCAGAGTCGATGGGTATTGA - - TCAGGGAGAGT	-210 (×11)<br>-2 (×14)	biallelic	TATATCCATTTG - …<br>TATATCCATTT t GCTGCTATAGTAGGGCAAGATG	-210 (×11)<br>+1 (×14)	biallelic
	CCAGAGTCCATGGGTATTGA - - TCAGGGAGAGT<br>CCAGAGTCCATGGGTATTGAA - TCAGGGAGAGT	-2 (×9)<br>-1 (×16)	biallelic	TGTATCCATTTGCTGCAATAGTAGGGCAAGATG	0 (×25)	WT

Table S4 Analysis of potential off-target sites among the Cas9/gRNA-induced *ptrchli1* mutants.

On/Off targets	Sequence name	Bases position of gRNAs																				PAM			Target gene or genomic position	Off-target Editing (Y/N)
		1	2	3	4	5	6	7	8	9	10	11	12	13	14	15	16	17	18	19	20	21	22	23		
**On**	**gCHLI1-a**	T	T	T	A	C	T	T	C	C	A	G	A	A	A	T	T	A	A	G	G	T	G	G	**CHLI1**	-
**Off**	gCHLI1a-off-1	T	T	T	A	C	T	T	C	C	T	G	A	A	A	T	T	A	A	G	G	T	A	G	CHLI2	No
	gCHLI1a-off-2	T	T	T	A	C	T	T	G	C	A	T	A	A	A	T	T	A	A	G	G	T	G	G	Chr09 (+4335300)	No
	gCHLI1a-off-3	C	T	T	A	C	C	A	C	C	A	G	A	A	A	T	T	A	A	A	G	T	G	G	Chr13 (-9790031)	No
	gCHLI1a-off-4	G	T	C	A	C	T	T	T	C	A	G	A	A	A	T	T	A	A	G	G	A	G	G	Potri.006G228600	No
	gCHLI1a-off-5	A	C	T	C	C	C	T	C	C	A	G	A	A	A	T	T	A	T	G	G	T	G	G	Chr12 (-2022439)	No
**On**	**gCHLI1-b**	C	C	T	A	A	C	A	G	T	G	G	T	G	G	A	C	T	T	T	C	C	G	G	**CHLI1**	-
**Off**	gCHLI1b-off-1	C	C	T	A	A	C	A	G	T	C	G	T	G	G	A	C	T	T	C	C	C	C	G	CHLI2	No
	gCHLI1b-off-2	A	T	T	A	G	A	A	T	T	G	G	T	G	G	A	C	T	T	T	C	C	G	G	Potri.018G015300	No
	gCHLI1b-off-3	G	T	A	A	A	C	C	G	T	G	G	T	G	G	A	C	T	T	T	C	T	G	A	Potri.013G004900	No

Nucleotides in green shading are the same with the designed gRNAs target sites and mismatched nucleotides are without shading. The PAM regions are highlighted in red shading. Potential off-target sites in protein coding gene regions are annotated with gene ID and the others located in noncoding regions are detailed with position information on chromosomes.

**gCHLI1a-off-1**		**Number of plasmids containing the cloned PCR products**
WT	TTTACTTCCTGAAATTAAGGTAG	(×10)
ptrchli1-1#	TTTACTTCCTGAAATTAAGGTAG	(×15)
ptrchli1-4#	TTTACTTCCTGAAATTAAGGTAG	(×15)
ptrchli1-5#	TTTACTTCCTGAAATTAAGGTAG	(×15)
ptrchli1-11#	TTTACTTCCTGAAATTAAGGTAG	(×15)
ptrchli1-14#	TTTACTTCCTGAAATTAAGGTAG	(×15)
ptrchli1-15#	TTTACTTCCTGAAATTAAGGTAG	(×15)

**gCHLI1a-off-2**		
WT	TTTACTTGCATAAATTAAGGTGG	(×10)
ptrchli1-1#	TTTACTTGCATAAATTAAGGTGG	(×15)
ptrchli1-8#	TTTACTTGCATAAATTAAGGTGG	(×15)
ptrchli1-11#	TTTACTTGCATAAATTAAGGTGG	(×15)
ptrchli1-12#	TTTACTTGCATAAATTAAGGTGG	(×15)
ptrchli1-15#	TTTACTTGCATAAATTAAGGTGG	(×15)
ptrchli1-16#	TTTACTTGCATAAATTAAGGTGG	(×15)

gCHLI1a-off-3

WT	CTTACCACCAGAAATTAAAGTGG	(×10)
ptrchli1-1#	CTTACCACCAGAAATTAAAGTGG	(×15)
ptrchli1-2#	CTTACCACCAGAAATTAAAGTGG	(×15)
ptrchli1-7#	CTTACCACCAGAAATTAAAGTGG	(×15)
ptrchli1-10#	CTTACCACCAGAAATTAAAGTGG	(×15)
ptrchli1-16#	CTTACCACCAGAAATTAAAGTGG	(×15)
ptrchli1-18#	CTTACCACCAGAAATTAAAGTGG	(×15)

gCHLI1a-off-4

WT	GTCACTTTCAGAAATTAAGGAGG	(×10)
ptrchli1-3#	GTCACTTTCAGAAATTAAGGAGG	(×15)
ptrchli1-4#	GTCACTTTCAGAAATTAAGGAGG	(×15)
ptrchli1-5#	GTCACTTTCAGAAATTAAGGAGG	(×15)
ptrchli1-7#	GTCACTTTCAGAAATTAAGGAGG	(×15)
ptrchli1-9#	GTCACTTTCAGAAATTAAGGAGG	(×15)
ptrchli1-10#	GTCACTTTCAGAAATTAAGGAGG	(×15)

gCHLI1a-off-5

WT	ACTCCCTCCAGAAATTATGGTGG	(×10)
ptrchli1-3#	ACTCCCTCCAGAAATTATGGTGG	(×15)
ptrchli1-4#	ACTCCCTCCAGAAATTATGGTGG	(×15)
ptrchli1-5#	ACTCCCTCCAGAAATTATGGTGG	(×15)
ptrchli1-11#	ACTCCCTCCAGAAATTATGGTGG	(×15)
ptrchli1-12#	ACTCCCTCCAGAAATTATGGTGG	(×15)
ptrchli1-14#	ACTCCCTCCAGAAATTATGGTGG	(×15)

gCHLI1b-off-1

WT	CGGGGAAGTCCACGACTGTTAGG	(×10)
ptrchli1-1#	CGGGGAAGTCCACGACTGTTAGG	(×15)
ptrchli1-4#	CGGGGAAGTCCACGACTGTTAGG	(×15)
ptrchli1-5#	CGGGGAAGTCCACGACTGTTAGG	(×15)
ptrchli1-11#	CGGGGAAGTCCACGACTGTTAGG	(×15)
ptrchli1-14#	CGGGGAAGTCCACGACTGTTAGG	(×15)
ptrchli1-15#	CGGGGAAGTCCACGACTGTTAGG	(×15)

gCHLI1b-off-2

WT	CCGGAAAGTCCACCAATTCTAAT	(×10)
ptrchli1-1#	CCGGAAAGTCCACCAATTCTAAT	(×15)
ptrchli1-11#	CCGGAAAGTCCACCAATTCTAAT	(×15)
ptrchli1-12#	CCGGAAAGTCCACCAATTCTAAT	(×15)
ptrchli1-15#	CCGGAAAGTCCACCAATTCTAAT	(×15)
ptrchli1-16#	CCGGAAAGTCCACCAATTCTAAT	(×15)
ptrchli1-17#	CCGGAAAGTCCACCAATTCTAAT	(×15)

gCHLI1b-off-3

WT	TCAGAAAGTCCACCACGGTTTAC	(×10)
ptrchli1-4#	TCAGAAAGTCCACCACGGTTTAC	(×15)
ptrchli1-5#	TCAGAAAGTCCACCACGGTTTAC	(×15)
ptrchli1-7#	TCAGAAAGTCCACCACGGTTTAC	(×15)
ptrchli1-11#	TCAGAAAGTCCACCACGGTTTAC	(×15)
ptrchli1-12#	TCAGAAAGTCCACCACGGTTTAC	(×15)
ptrchli1-14#	TCAGAAAGTCCACCACGGTTTAC	(×15)

Table S5 The Cas9/gRNA-targeted mutations in *PtrCesA4, 7A, 7B, 7A/B, 8A, 8B* and *8A/B* genes of *Populus trichocarpa*.

Gene: Potri.002G257900 (*PtrCesA4*)

Vector: Cas9/gRNA-PtrCesA4-1
gRNA: gCesA4-1 (CATACGATGCTTATGCATTGTGG); gCesA4-2 (CCCATTCGTCCGGCCTTTTCGTC)
Target sequences: CesA4-1, CATACGATGCTTATGCATTGTGG; CesA4-2, CCCATTCGTCCGGCCTTTTCGTC

**Line (#)**	**gCesA4-1**<br>CATACGATGCTTATGCATTGTGG	**Mutation types**<br>(Number of plasmids containing the cloned PCR products)		**gCesA4-2**<br>CCCATTCGTCCGGCCTTTTCGTC	**Mutation types**<br>(Number of plasmids containing the cloned PCR products)	
ptrcesa4-1#	TCCAGCATACGATGCTTATGC - TTGTGGCTTAT<br>TCCAGCATACGATGCTTATGCATTGTGGCTTAT	-1 (×14)<br>0 (×11)	heterozygous	TGCACCCCATTCGT - - - GCCTTTTCGTCAGCAG<br>TGCACCCCATT a CGTCCGGCCTTTTCGTCAGCAG	-3 (×14)<br>+1 (×11)	biallelic
ptrcesa4-2#	TCCAGCATACGATGCTTATGC - TTGTGGCTTAT<br>TCCAGCATACGATGCTTATGCATTGTGGCTTAT	-1 (×17)<br>0 (×8)	heterozygous	TGCACCCCATT c CGTCCGGCCTTTTCGTCAGCAG<br>TGCACCCCATT t CGTCCGGCCTTTTCGTCAGCAG	+1 (×15)<br>+1 (×10)	biallelic
ptrcesa4-3#	- -- - - GTGGCTTAT<br>TCCAGCATACGATGCTTATGCA a TTGTGGCTTAT	-127 (×16)<br>+1 (×9)	biallelic	TGCACCCCATT - - - - - - - - - - - - TCGTCAGCAG<br>TGCACCCCATT a CGTCCGGCCTTTTCGTCAGCAG	-12 (×16)<br>+1 (×9)	biallelic
ptrcesa4-4#	TCCAGCATACGATGCTTATGCA a TTGTGGCTTAT<br>TCCAGCATACGATGCTTATGCATTGTGGCTTAT	+1 (×11)<br>0 (×14)	heterozygous	TGCACCCCATT - - - - - GGCCTTTTCGTCAGCAG	-5 (×25)	homozygous

Vector: Cas9/gRNA-PtrCesA4-2
gRNA: gCesA4-3 (CATCTGTGTTAGAACTTCGATGG); gCesA4-4 (CCTCCTCAAGGTCCTTGGAGGAG)
Target sequences: CesA4-3, CATCTGTGTTAGAACTTCGATGG; CesA4-4, CCTCCTCAAGGTCCTTGGAGGAG

**Line (#)**	**gCesA4-3**<br>CATCTGTGTTAGAACTTCGATGG	**Mutation types**<br>(Number of plasmids containing the cloned PCR products)		**gCesA4-4**<br>CCCATTCGTCCGGCCTTTTCGTC	**Mutation types**<br>(Number of plasmids containing the cloned PCR products)	
ptrcesa4-5#	AGCAACATCTGTGTTAGAA - - - CGATGGAGTGG<br>AGCAACATCTGTGTTAGAAC - - CGATGGAGTGG	-3 (×13)<br>-2 (×12)	biallelic	CAAGGCCTCCTCAAGGTCCTTGGAGGAGTAGAC	0 (×25)	WT
ptrcesa4-6#	AGCAACATCTGTGTTAGAACT - CGATGGAGTGG<br>AGCAACATCTGTGTTAGAACT - - - - - - - - - - - - -- -	-1 (×12)<br>-127 (×13)	biallelic	CAAGGCCTCCT - AAGGTCCTTGGAGGAGTAGAC<br>- AACTTCACTGTTA	-1 (×12)<br>-127 (×13)	biallelic

Gene: Potri.006G181900 (*PtrCesA7A*)

Vector: Cas9/gRNA-PtrCesA7A-1
gRNA: gCesA7A-a (CTATTATTGCAGGTTAGACGAGG); gCesA7A-b (CCCTATTGGATCTCATGGAGAGC)
Target sequences: CesA7A-a, CTATTATTGCAGGTTAGACGAGG; CesA7A-b, CCCTATTGGATCTCATGGAGAGC

Line (#)	gCesA7A-a<br>CTATTATTGCAGGTTAGACGAGG	Mutation types (Number of plasmids containing the cloned PCR products)		gCesA7A-b<br>CCCTATTGGATCTCATGGAGAGC	Mutation types (Number of plasmids containing the cloned PCR products)	
ptrcesa7A-1#	GTGAACTATTATTGCAGGTTA - ACGAGGCAAGA	-1 (×25)	homozygous	GAGTTCCCTAT - GGATCTCATGGAGAGCAGATG	-1 (×25)	homozygous
ptrcesa7A-2#	GTGAACTATTATTGCAGGTT - - ACGAGGCAAGA<br>GTGAACTATTATTGCAGGTTAG g ACGAGGCAAGA	+1 (×15)<br>-2 (×10)	biallelic	GAGTTCCCTATcTGGATCTCATGGAGAGCAGAT<br>GAGTTCCCTAT - - - - - CTCATGGAGAGCAGATG	+1 (×10)<br>-5 (×15)	biallelic

Vector: Cas9/gRNA-PtrCesA7A-2
gRNA: gCesA7A-c (GACAGGAATGGATAACGAGAAGG); gCesA7A-d (CCATGACACTGAAGGAAATGAGC)
Target sequences: CesA7A-c, GACAGGAATGGATAACGAGAAGG; CesA7A-d, CCATGACACTGAAGGAAATGAGC

Line (#)	gCesA7A-c<br>GACAGGAATGGATAACGAGAAGG	Mutation types (Number of plasmids containing the cloned PCR products)		gCesA7A-d<br>CCATGACACTGAAGGAAATGAGC	Mutation types (Number of plasmids containing the cloned PCR products)	
ptrcesa7A-3#	(inverted 834) AGAAGGAGCTA	inversion (×25)	homozygous	GGAGGCCATGA (inverted 834)<br>GGAGGCCAT - - (-2+inverted 834)	inversion (×17)<br>inversion (×8)	biallelic
ptrcesa7A-4#	(inverted 834) AGAAGGAGCTA	inversion (×25)	homozygous	GGAGGCCATGA (inverted 834)	inversion (×25)	homozygous
ptrcesa7A-5#	TGCTGGACAGGAATGGATAAC - AGAAGGAGCTA<br>TGCTGGACAGGAATGGATAACGtAGAAGGAGCTA	-1 (×19)<br>+1 (×6)	biallelic	GGAGGCCATGACACTGAAGGAAATGAGCTCCCT<br>GGAGGCCATGAaCACTGAAGGAAATGAGCTCCCT	+1 (×19)<br>0 (×6)	heterozygous
ptrcesa7A-6#	TGCTGGACAGGAATGGATAAC - - GAAGGAGCTA	-2 (×25)	homozygous	GGAGGCCATGAgCACTGAAGGAAATGAGCTCCCT<br>GGAGGCCATGACACTGAAGGAAATGAGCTCCCT	+1 (×8)<br>0 (×17)	heterozygous
ptrcesa7A-7#	TGCTGGACAGGAATGGATAAC - AGAAGGAGCTA	-1 (×25)	homozygous	GGAGGCCATGA - ACTGAAGGAAATGAGCTCCCT	-1 (×25)	homozygous

Gene: Potri.018G103900 (*PtrCesA7B*)

Vector: Cas9/gRNA-PtrCesA7B-1
gRNA: gCesA7B-a (ACAAGATGACGATGCAGAAGCGG); gCesA7B-b (CCATTGGATCTCATGGAGAACAG)
Target sequences: CesA7B-a, ACAAGATGACGATGCAGAAGCGG; CesA7B-b, CCATTGGATCTCATGGAGAACAG

Line (#)	gCesA7B-a<br>ACAAGATGACGATGCAGAAGCGG	Mutation types (Number of plasmids containing the cloned PCR products)		gCesA7B-b<br>CCATTGGATCTCATGGAGAACAG	Mutation types (Number of plasmids containing the cloned PCR products)	
ptrcesa7B-1#	CCTGAACAAGATGACGATGCAG - AGCGGCCATG<br>CCTGAACAAGATGACGATGCAGAAGCGGCCATG	+1 (×16)<br>0 (×9)	heterozygous	GTTCTCCATTG t GATCTCATGGAGAACAGATGCT<br>GTTCTCCATTG - ATCTCATGGAGAACAGATGCT	+1 (×19)<br>-1 (×6)	biallelic
ptrcesa7B-2#	CCTGAACAAG - - - - - - - - - - - - AAGCGGCCATG<br>CCTGAACAAGATGACGAT - - - - AAGCGGCCATG	-12 (×10)<br>-4(×25)	biallelic	GTTCTCCATTG - ATCTCATGGAGAACAGATGCT<br>GTTCTCCATTG t GATCTCATGGAGAACAGATGCT	-1 (×11)	biallelic

					+1 (×14)	
**Vector:** Cas9/gRNA-PtrCesA7B-2<br>**gRNA:** gCesA7B-c (GCCCAATATGCTTGCTCCAGTGG); gCesA7B-d (CCAGCAAGATCAATCCGTATAGA)<br>**Target sequences:** CesA7B-c, GCCCAATATGCTTGCTCCAGTGG; CesA7B-d, CCAGCAAGATCAATCCGTATAGA						
**Line (#)**	**gCesA7B-c**<br>GCCCAATATGCTTGCTCCAGTGG	**Mutation types**<br>(Number of plasmids containing the cloned PCR products)		**gCesA7B-d**<br>CCAGCAAGATCAATCCGTATAGA	**Mutation types**<br>(Number of plasmids containing the cloned PCR products)	
ptrcesa7B-3#	(inverted 407) CAGTGGATATC<br>GGCGAGCCCAATATGCTTGCTC t CAGTGGATATC	inversion (×15)<br>+1 (×10)	biallelic	TGCATCCAGCA (inverted 407)<br>TGCATCCAGCA - GATCAATCCGTATAGAATGGT	inversion (×15)<br>-1 (×10)	biallelic
ptrcesa7B-4#	(inverted 407) CAGTGGATATC	inversion (×25)	homozygous	TGCATCCAGCA (inverted 407)	inversion (×25)	homozygous
ptrcesa7B-5#	(inverted 407) CAGTGGATATC<br>GGCGAGCCCAATATGCTTGC - - CAGTGGATATC	inversion (×11)<br>-2 (×14)	biallelic	TGCATCCAGCA (inverted 407)<br>TGCATCCAGCA - GATCAATCCGTATAGAATGGT	inversion (×11)<br>-1 (×14)	biallelic
ptrcesa7B-6#	(inverted 407) CAGTGGATATC<br>GGCGAGCCCAATATGCTTGC - - CAGTGGATATC	inversion (×16)<br>-2 (×9)	biallelic	TGCATCCAGCA (inverted 407)<br>TGCATCCAGCA - GATCAATCCGTATAGAATGGT	inversion (×16)<br>-1 (×9)	biallelic
ptrcesa7B-7#	GGCGAGCCCAATATGC - - - - - - CAGTGGATATC<br>GGCGAGCCCAATATGCTTGCTC CAGTGGATATC	-6 (×18)<br>0 (×7)	heterozygous	TGCATCCAGCAA - GATCAATCCGTATAGAATGGT<br>TGCATCCAGCAAGATCAATCCGTATAGAATGGT	-1 (×18)<br>0 (×7)	heterozygous
ptrcesa7B-8#	(inverted 407) CAGTGGATATC<br>GGCGAGCCCAATATGCTTGC - - CAGTGGATATC	inversion (×13)<br>-2 (×12)	biallelic	TGCATCCAGCA (inverted 407)<br>TGCATCCAGCA - GATCAATCCGTATAGAATGGT	inversion (×13)<br>-1 (×12)	biallelic
**Gene:** Potri.011G069600 (*PtrCesA8A*)						
**Vector:** Cas9/gRNA-PtrCesA8A-1<br>**gRNA:** gCesA8A-a (TTGTTTCGAGTATGAGATCAAGG); gCesA8A-b (CCAGGTGGGACATGATGCAAATG)<br>**Target sequences:** CesA8A-a, TTGTTTCGAGTATGAGATCAAGG; CesA8A-b, CCAGGTGGGACATGATGCAAATG						
**Line (#)**	**gCesA8A-a**<br>TTGTTTCGAGTATGAGATCAAGG	**Mutation types**<br>(Number of plasmids containing the cloned PCR products)		**gCesA8A-b**<br>CCAGGTGGGACATGATGCAAATG	**Mutation types**<br>(Number of plasmids containing the cloned PCR products)	
ptrcesa8A-1#	... - ATCAAGGAGGGG	-82 (×25)	homozygous	GGTGACCAGGT - ...	-82 (×25)	homozygous
ptrcesa8A-2#	... - ATCAAGGAGGGG<br>AAGTCTTGTTTCGAGTATGAGAcTCAAGGAGGGG	-82 (×19)<br>+1 (×6)	biallelic	GGTGACCAGGT - ...<br>GGTGACCAGGT - - GACATGATGCAAATGGAGAC	-82 (×19)<br>-2 (×6)	biallelic

Vector: Cas9/gRNA-PtrCesA8A-2
gRNA: gCesA8A-c (GGGAAATGAACTACCTCGACTGG); gCesA8A-d (CCGGAGCTCGTGACATAGAGGGA)
Target sequences: CesA8A-c, GGGAAATGAACTACCTCGACTGG; CesA8A-d, CCGGAGCTCGTGACATAGAGGGA

Line (#)	**gCesA8A-c**<br>GGGAAATGAACTACCTCGACTGG	**Mutation types**<br>(Number of plasmids containing the cloned PCR products)		**gCesA8A-d**<br>CCGGAGCTCGTGACATAGAGGGA	**Mutation types**<br>(Number of plasmids containing the cloned PCR products)	
ptrcesa8A-3#	ATAGAGGGAAATGAACTACCT - - ACTGGTATAT	-2 (×25)	homozygous	AAACACCGGAGa TCGTGACATAGAGGGAAATGA	+1 (×25)	homozygous
ptrcesa8A-4#	ATAGAGGGAAATGAACTACCTC - ACTGGTATAT<br>ATAGAGGGAAATGAACTACCT - - ACTGGTATAT	-1 (×9)<br>-2 (×16)	biallelic	AAACACCGGAGg TCGTGACATAGAGGGAAATGA<br>AAACACCGGAGaCTCGTGACATAGAGGGAAATGA	+1 (×9)<br>+1 (×16)	biallelic
ptrcesa8A-5#	ATAGAGGGAAATGAACTACCTCGACTGGTATAT	0 (×25)	WT	AAACACCGGAG - - - - - - - - - - - - AGGGAAATGA	-12 (×25)	homozygous
ptrcesa8A-6#	ATAGAGGGAAATGAACTA - - - - - ACTGGTATAT	-5 (×25)	homozygous	... - - - - - - - - - - - - -CGTGACATAGAGGGAAATGA	-16 (×25)	homozygous
ptrcesa8A-7#	... - GACTGGTATAT	-30 (×25)	homozygous	AAACACCGGAG - - - - - - - - - - - - - - - - - - - ...	-30 (×25)	homozygous

Gene: Potri.004G059600 (*PtrCesA8B*)

Vector: Cas9/gRNA-PtrCesA8B-1
gRNA: gCesA8B-a (AGGGCCGGAAAGTTTGCTTGCGG); gCesA8B-b (CCTGAAAATCCCTTCCATTTGCT)
Target sequences: CesA8B-a, AGGGCCGGAAAGTTTGCTTGCGG; CesA8B-b, CCTGAAAATCCCTTCCATTTGCT

Line (#)	**gCesA8B-a**<br>AGGGCCGGAAAGTTTGCTTGCGG	**Mutation types**<br>(Number of plasmids containing the cloned PCR products)		**gCesA8B-b**<br>CCTGAAAATCCCTTCCATTTGCT	**Mutation types**<br>(Number of plasmids containing the cloned PCR products)	
ptrcesa8B-1#	CAAAGAGGGCCGGAAAGTTTGC - - - - - - - - - - - ...	-172 (×25)	homozygous	... - - - - - - - - - - -AATCCCTTCCATTTGCTTGTTA	-172 (×25)	homozygous
ptrcesa8B-2#	CAAAGAGGGCCGGAAAGTTTGC t TTGCGGTGCGG	+1 (×25)	homozygous	ATATTCCTGAA - ATCCCTTCCATTTGCTTGTTA	-1 (×25)	homozygous
ptrcesa8B-3#	CAAAGAGGGCCGGAAAGTTTGC a TTGCGGTGCGG<br>CAAAGAGGGCCGGAAAGTTTGCTTGCGGTGCGG	+1 (×22)<br>0 (×3)	heterozygous	ATATTCCTGAA t AATCCCTTCCATTTGCTTGTTA<br>ATATTCCTGAA - - TCCCTTCCATTTGCTTGTTA	+1 (×13)<br>-2 (×12)	heterozygous

Vector: Cas9/gRNA-PtrCesA8B-2
gRNA: gCesA8B-c (GGTGAGCCTTCTCAGCTTGCTGG); gCesA8B-d (CCTGCTTTCTCCAGAGAACTTGC)
Target sequences: CesA8B-c, GGTGAGCCTTCTCAGCTTGCTGG; CesA8B-d, CCTGCTTTCTCCAGAGAACTTGC

Line (#)	**gCesA8B-c**<br>GGTGAGCCTTCTCAGCTTGCTGG	**Mutation types**<br>(Number of plasmids containing the cloned PCR products)	**gCesA8B-d**<br>CCTGCTTTCTCCAGAGAACTTGC	**Mutation types**<br>(Number of plasmids containing the cloned PCR products)

ptrcesa8B-4#	GAGAGGGTGAGCCTTCTC - - - - TGCTGGTGTGG<br>GAGAGGGTGAGCCTTCTCAGCTTGCTGGTGTGG	-4 (×19)<br>0 (×6)	heterozygous	TTATGCCTGCTTTCTCCAGAGAACTTGCTGGAT	0 (×25)	WT
ptrcesa8B-5#	GAGAGGGTGAGCCTTCTCAGC - TGCTGGTGTGG	-1 (×25)	homozygous	TTATGCCTGCT - - - - CCAGAGAACTTGCTGGAT	-4 (×25)	homozygous

Gene: Potri.006G181900 (*PtrCesA7A*)/Potri.018G103900 (*PtrCesA7B*)

Vector: Cas9/gRNA-PtrCesA7A/B-1
gRNA: gCesA7A/B-1 (ACGCAAAGAAAGAGGGAGGGTGG); gCesA7A/B-2 (CCATATCCAGTTTCTGAACCTGG)
Target sequences: CesA7A-1, ACGCAAAGAAAGAGGGAGGGTGG; CesA7A-2, CCATATCCAGTTTCTGAACCTGG
CesA7B-1, ACGAAAAGAAAGAGGGAGGGTGG; CesA7B-2, CCATATCCAGTTTCTGAACCTGG

**Line (#)**	**gCesA7A/B-1**<br>CesA7A, ACGCAAAGAAAGAGGGAGGGTGG<br>CesA7B, ACGAAAAGAAAGAGGGAGGGTGG	**Mutation types**<br>(Number of plasmids containing the cloned PCR products)		**gCesA7A/B-2**<br>CesA7A, CCATATCCAGTTTCTGAACCTGG<br>CesA7B, CCATATCCAGTTTCTGAACCTGG	**Mutation types**<br>(Number of plasmids containing the cloned PCR products)	
***ptrcesa7ab-1#*-7A**	... - AGGGTGGAAAGA	-138 (×25)	homozygous	TGCACCCATAT - ...	-138 (×25)	homozygous
**-7B**	ATGGGACGAAAAGAAAGAGG - - GGGTGGAAAGA	-2 (×25)	homozygous	TGCACCA - - - - CCAGTTTCTGAACCTGGTATTT	-4 (×25)	homozygous
***ptrcesa7ab-2#*-7A**	... - AAAGA<br>... - GGGTGGAAAGA	-39 (×17)<br>-139 (×8)	biallelic	TGCACCCATA - CCAGTTTCTGAACCTGGTATTT<br>TGCACCCATAT - - - - - - - - - - - - - - - - - - - ...<br>TGCACCCATATCCAGTTTCTGAACCTGGTATTT	-1 (×10)<br>-139 (×8)<br>0 (×7)	chimera
**-7B**	... - - - - - - - - -- - - - - - - - - - - - - - - GGTGGAAAGA	-133 (×25)	homozygous	TGCACCCAT- ...	-133 (×25)	homozygous
***ptrcesa7ab-3#*-7A**	... - TCGCCTTTCTCTC<br>... - - - - - - - - - - - - - - - - - -- - AGGGTGGAAAGA	-645 (×11)<br>-315 (×14)	biallelic	TGGATCTCATGGA - - - - - - - - - - - - - - - - - - …<br>GTCAGATCAAGAC - - - - - - - - - - - - - - - - - - …	-645 (×11)<br>-315 (×14)	biallelic
**-7B**	... - - - - - - - - - - - - - - - - - - -- - GGGTGGAAAGA<br>ATGGGACGAAAAGAAAGAGGGAaGGGTGGAAAGA	-129 (×13)<br>+1 (×12)	biallelic	TGCACCCATATC - …<br>TGCACCCATAT c CCAGTTTCTGAACCTGGTATTT	-129 (×13)<br>+1 (×12)	biallelic
***ptrcesa7ab-4#*-7A**	TGGGACGCAAAGAAAGAGGGAaGGGTGGAAAG	+1 (×25)	homozygous	TGCACCCATATC^276CAGTTTCTGAACCTGGTATT	+276 (×25)	homozygous
**-7B**	TGGGACGAAAAGAAAGAGGG - GGGTGGAAAG<br>TGGGACGAAAAGAAAGAG - - - - - - - - - - - - - - -	-1 (×11)<br>-15 (×14)	biallelic	TGCACCCAT - - - - - - - - TCTGAACCTGGTATTT<br>TGCACCC - - - - - CAGTTTCTGAACCTGGTATTT	-8 (×11)<br>-5 (×14)	biallelic

Vector: Cas9/gRNA-PtrCesA7A/B-2
gRNA: gCesA7A/B-3 (TTTTGTGACTTCAACTTTAATGG); gCesA7A/B-4 (CCTCCTTCCTCGAGTCCAGCGGC)
Target sequences: CesA7A-3, TTTTGTGACTTCAACTTTAATGG; CesA7A-4, CCTCCTTCCTCGAGTCCAGCGGC
CesA7B-3, TTTTGTGACTTCAACTTTAATGG; CesA7B-4, CCTCCTTCCTCGAGTCCGGCAGC

**Line (#)**	**gCesA7A/B-3**<br>CesA7A, TTTTGTGACTTCAACTTTAATGG<br>CesA7B, TTTTGTGACTTCAACTTTAATGG	**Mutation types**<br>(Number of plasmids containing the cloned PCR products)		**gCesA7A/B-4**<br>CesA7A, CCTCCTTCCTCGAGTCCAGCGGC<br>CesA7B, CCTCCTTCCTCGAGTCCGGCAGC	**Mutation types**<br>(Number of plasmids containing the cloned PCR products)	
***ptrcesa7ab-5#*-7A**	GCAATTTTTGTGACTTCAACTTTAATGGAAGAA	0 (×25)	WT	GCGTACCTCCT - - - - CGAGTCCAGCGGCTCTGC	-4 (×16)	biallelic

**-7B**				GCGTACC - - - TTCCTCGAGTCCAGCGGCTCTGC	-3 (×9)	
	GCAATTTTTGTGACTTCAACTTTAATGGAAGAA	0 (×25)	WT	GTGTACCTCCT t TCCTCGAGTCCGGCAGCTCTGC<br>GTGTACCTCCT - CCTCGAGTCCGGCAGCTCTGC	+1 (×15)<br>-1 (×10)	biallelic
***ptrcesa7ab-6#*-7A**	GCAATTTTTGTGACTTCAACTTTAATGGAAGAA	0 (×25)	WT	GCGTACCTCCT - CCTCGAGTCCAGCGGCTCTGC<br>GCGTACCTC - - - - - - - GAGTCCAGCGGCTCTGC<br>GCGTACCTCCTTCCTCGAGTCCAGCGGCTCTGC	-1 (×13)<br>-7 (×9)<br>0 (×3)	chimeric
**-7B**	GCAATTTTTGTGACTTCAACTTTAATGGAAGAA	0 (×25)	WT	GTGTACCTCCT a TCCTCGAGTCCGGCAGCTCTGC<br>GT - - - - - - - - - - CCTCGAGTCCGGCAGCTCTGC<br>GTGTACCTCCTTCCTCGAGTCCGGCAGCTCTGC	+1 (×9)<br>-10 (×12)<br>0 (×4)	chimeric
***ptrcesa7ab-7#*-7A**	GCAATTTTTGTGACTTCAACTTTAATGGAAGAA	0 (×25)	WT	GCGTACCTCCTT a CCTCGAGTCCAGCGGCTCTGC	+1 (×25)	homozygous
**-7B**	GCAATTTTTGTGACTTCAACTTTAATGGAAGAA	0 (×25)	WT	GTGTACCTCCT - - - - CGAGTCCGGCAGCTCTGC	-4 (×25)	homozygous
***ptrcesa7ab-8#*-7A**	GCAATTTTTGTGACTTCAACTTTAATGGAAGAA	0 (×25)	WT	GCGTACCTCCTT a CCTCGAGTCCAGCGGCTCTGC	+1 (×25)	homozygous
**-7B**	GCAATTTTTGTGACTTCAACTTTAATGGAAGAA	0 (×25)	WT	GTGTACCTCCT - - - - CGAGTCCGGCAGCTCTGC<br>GTGTACC^14TCC^4TTCCTCGAGTCCGGCAGCTCTGC（^14: aagcttattagctc ^4: acca）	-4 (×12)<br>+18 (×13)	biallelic
***ptrcesa7ab-9#*-7A**	GCAATTTTTGTGACTTCAACTTTAATGGAAGAA	0 (×25)	WT	GCGTACCTCCTT a CCTCGAGTCCAGCGGCTCTGC<br>GCGTACCTC - - - - - - - GAGTCCAGCGGCTCTGC	+1 (×11)<br>-7 (×6)	biallelic
**-7B**	GCAATTTTTGTGACTTCAACTTTAATGGAAGAA	0 (×25)	WT	GTGTACCTCCT t TCCTCGAGTCCGGCAGCTCTGC<br>GTGTACCTCCTTCCTCGAGTCCGGCAGCTCTGC	+1 (×18)<br>0 (×7)	heterozygous
***ptrcesa7ab-10#*-7A**	GCAATTTTTGTGACTTCAACTTTAATGGAAGAA	0 (×25)	WT	GCGTACCTCCT - CCTCGAGTCCAGCGGCTCTGC<br>GCGTACCTCC - - - CTCGAGTCCAGCGGCTCTGC	-1 (×13)<br>-3 (×12)	biallelic
**-7B**	GCAATTTTTGTGACTTCAACTTTAATGGAAGAA	0 (×25)	WT	GTGTACCTCCT a TCCTCGAGTCCGGCAGCTCTGC<br>GTGTACCTCCT - CCTCGAGTCCGGCAGCTCTGC	+1 (×14)<br>-1 (×11)	biallelic
***ptrcesa7ab-11#*-7A**	GCAATTTTTGTGACTTCAACTTTAATGGAAGAA	0 (×25)	WT	GCGTACCTCC - - CCTCGAGTCCAGCGGCTCTGC<br>GCGTACCTCCTT t CCTCGAGTCCAGCGGCTCTGC	-2 (×22)<br>+1 (×3)	biallelic
**-7B**	GCAATTTTTGTGACTTC - - - - - - - - - - - - - - - - …<br>GCAATTTTTGTGACTTCAACTTTAATGGAAGAA	-33 (×14)<br>0 (×11)	heterozygous	… - - - - - - - - - - - - - CTCGAGTCCGGCAGCTCTGC<br>GTGTACCTC - TTC	-33 (×14)<br>-21 (×11)	biallelic
***ptrcesa7ab-12#*-7A**	GCAATTTTTGTGACTTCAACTTTAATGGAAGAA	0 (×25)	WT	GCGTACCTCCT - - - - CGAGTCCAGCGGCTCTGC<br>GCGT - - - - - - - - CCTCGAGTCCAGCGGCTCTGC	-4 (×16)<br>-8 (×9)	biallelic
**-7B**	GCAATTTTTGTGACTTCAACTTTAATGGAAGAA	0 (×25)	WT	GTGTAC - - - CTTCCTCGAGTCCGGCAGCTCTGC<br>GTGTACCTCCT - CCTCGAGTCCGGCAGCTCTGC	-3 (×18)<br>-1 (×7)	biallelic
***prcesa7ab-13#*-7A**	GCAATTTTTGTGACTTCAACTTTAATGGAAGAA	0 (×25)	WT	GCGTACCTCCT - CCTCGAGTCCAGCGGCTCTGC<br>GCGTACCTC - - - - - - - GAGTCCAGCGGCTCTGC	-1 (×12)<br>-7 (×13)	biallelic
**-7B**	GCAATTTTTGTGACTTCAACTTTAATGGAAGAA	0 (×25)	WT	GT - - - - - - - - - - CCTCGAGTCCGGCAGCTCTGC	-10 (×25)	homozygous
**Gene:** Potri.011G069600 (*PtrCesA8A*)/ Potri.004G059600 (*PtrCesA8B*)						

Vector: Cas9/gRNA-PtrCesA8A/B-1
gRNA: gCesA8A/B-1 (AGTTCCGCCGGAACAGCAGATGG); gCesA8A/B-2 (CCACTACAGAATAACAAATCCTG)
Target sequences: CesA8A-1, AGTTCCGCCGGAACAGCAGATGG; CesA8A-2, CCACTACAGAATAACAAATCCTG
CesA8B-1, AGTTCCGCCGGAACAGCAGATGG; CesA8B-2, CCACTACAGAATAACAAATCCTG

**Line (#)**	**gCesA8A/B-1**<br>CesA8A-1, AGTTCCGCCGGAACAGCAGATGG<br>CesA8B-1, AGTTCCGCCGGAACAGCAGATGG	**Mutation types**<br>(Number of plasmids containing the cloned PCR products)		**gCesA8A/B-2**<br>CesA8A-2, CCACTACAGAATAACAAATCCTG<br>CesA8B-2, CCACTACAGAATAACAAATCCTG	**Mutation types**<br>(Number of plasmids containing the cloned PCR products)	
***ptrcesa8ab-1*#-8A**	GCTCAAGTTCCGCCGGAACA - - - GATGGAAGAG<br>GCTCAAGTTCCGCCGGAA - - - - - GATGGAAGAG	-3 (×23)<br>-5 (×2)	biallelic	TTCTTCCACTA - - - - - TAACAAATCCTGTTGAT<br>TTCTTCCACTA a CAGAATAACAAATCCTGTTGAT	-5 (×23)<br>+1 (×2)	biallelic
**-8B**	GCTCAAGTTCCGCCGGA - - - - GAAGACTAAGAT<br>GCTCAAGTTCCGCCG - - - - - - - - - ATGGAAGAT	-4 (×13)<br>-9 (×12)	biallelic	TTCTTCCACTA t CAGAATAACAAATCCTGTCGAT<br>TTCTTCCACTA a CAGAATAACAAATCCTGTCGAT	+1 (×11)<br>+1 (×14)	biallelic
***ptrcesa8ab-2*#-8A**	GCTCAAGTTCCGCCGGAACAGC a AGATGGAAG<br>GCTCAAGTTCCGCCGGAACAGCAGATGGAAGA	+1 (×15)<br>0 (×10)	heterozygous	TTCTTCCACTA - AGAATAACAAATCCTGTTGAT<br>TTCTTCCACTACAGAATAACAAATCCTGTTGAT	-1 (×10)<br>0 (×15)	heterozygous
**-8B**	... - AGATGGAAGAT	-279 (×25)	homozygous	TTCTTCCACTA t CAGAATAACAAATCCTGTCGAT	+1 (×25)	homozygous
***ptrcesa8ab-3*#-8A**	GCTCAAGTTCCGCCGGAACA - - - GATGGAAGAG<br>GCTCAAGTTCCGCCGGAA - - - - - GATGGAAGAG	-3 (×18)<br>-5 (×7)	biallelic	TTCTTCCACTACAGAATAACAAATCCTGTTGAT	0 (×25)	WT
**-8B**	GCTCAAGTTCCGCCGGA - - - - GAAGACTAAGAT<br>GCTCAAGTTCCGCCG - - - - - - - - - ATGGAAGAT	-4 (×13)<br>-9 (×12)	biallelic	TTCTTCCACTAC a AGAATAACAAATCCTGTCGAT<br>TTCTTCCACTA a CAGAATAACAAATCCTGTCGAT<br>TTCTTCCACTA - AGAATAACAAATCCTGTCGAT<br>TTCTTCCACTACAGAATAACAAATCCTGTCGAT	+1 (×10)<br>+1 (×2)<br>-1 (×10)<br>0 (×3)	chimeric

Vector: Cas9/gRNA-PtrCesA8A/B-2
gRNA: gCesA8A/B-3 (GAACTGGTTGTGTTTTCAATAGG); gCesA8A/B-4 (CCACAAGTAGGTCGAGATGTATG)
Target sequences: CesA8A-3, GAACTGGTTGTGTTTTCAATAGG; CesA8A-4, CCACAAGTAGGTCGAGATGTATG
CesA8B-3, GAACTGGTTGTGTTTTCAATAGG; CesA8B-4, CCACAAGTAGGTCGAGATGTATG

**Line#**	**gCesA8A/B-3**<br>CesA8A-3, GAACTGGTTGTGTTTTCAATAGG<br>CesA8B-3, GAACTGGTTGTGTTTTCAATAGG	**Mutation types**<br>(Number of plasmids containing the cloned PCR products)		**gCesA8A/B-4**<br>CesA8A-4, CCACAAGTAGGTCGAGATGTATG<br>CesA8B-4, CCACAAGTAGGTCGAGATGTATG	**Mutation types**<br>(Number of plasmids containing the cloned PCR products)	
***ptrcesa8ab-4*#-8A**	TGTGGGAACTGGTTGTGTTTTC t AATAGGCAAG	+1 (×25)	homozygous	TGGATCCACAA g GTAGGTCGAGATGTATGCTAT<br>TGGATCCACAAGTAGGTCGAGATGTATGCTATG	+1 (×3)<br>0 (×22)	heterozygous
**-8B**	TGTGGGAACTGGTTGTGTTTTC a AATAGGCAAG<br>TGTGGGAACTGGTTGTGTTTTC t AATAGGCAAG	+1 (×23)<br>+1 (×2)	biallelic	TGGATCCACAAGTAGGTCGAGATGTATGCTATG	0 (×25)	WT
***ptrcesa8ab-5*#-8A**	(inverted 687) CAATAGGCAAGC<br>… - GGCAAGC	inversion (×13)<br>-28 (×12)	biallelic	TGGATCCACAAG (inverted 687)<br>TGGATCCACAAGTAGGTCGAGATGTATGCTATG	inversion (×13)<br>0 (×12)	heterozygous
**-8B**	TGTGGGAACTGGTGA - - - - - - - AAAGGCAAGC<br>TGTGGGAACTGGTTGTG - - - - - - - - - GGCAAGC	-7 (×21)<br>-9 (×4)	biallelic	TGGATCCACAA g GTAGGTCGAGATGTATGCTAT<br>TGGATCCACAAGTAGGTCGAGATGTATGCTATG	+1 (×4)<br>0 (×21)	heterozygous

***ptrcesa8ab-6#*-8A**<br>**-8B**	TGTGGGAACTGGTTGTGTTTTC t AATAGGCAAG<br>TGTGGGAACTGGT - - - - - - - - - - - - - - - - - - - …	+1 (×18)<br>-31 (×7)	biallelic	TGGATCCACAA a GTAGGTCGAGATGTATGCTAT<br>TGGATCCACAAGTAGGTCGAGATGTATGCTATG	+1 (×22)<br>0 (×3)	heterozygous
	TGTGGGAACTGGTTGTGTTTTC a AATAGGCAAG<br>TGTGGGAACTGGTTGTGTTTTCaaAATAGGCAAG<br>TGTGGGAACTGGTTGTGTA - - - AATAGGCAAGC	+1 (×15)<br>+2 (×2)<br>-3 (×8)	chimeric	TGGATCCACAAGTAGGTCGAGATGTATGCTATG	0 (×25)	WT
***ptrcesa8ab-7#*-8A**<br>**-8B**	… - AATAGGCAAGC	-688 (×25)	homozygous	TGGATCCACAAG - - - - - - - - - - - - - - - - - …	-688 (×25)	homozygous
	TGTGGGAACTGGTTGTGTTTTCtatAATAGGCAAG<br>TGTGGGAACTGGTT ^19^ TTTTGAATAGGCAAGC<br>(^19^: aacatgaggttaacaacga)	+3 (×14)<br>+19 (×11)	biallelic	TGGATCCACAAGTAGGTCGAGATGTATGCTATG	0 (×25)	WT
***ptrcesa8ab-8#*-8A**<br>**-8B**	TGTGGGAACTGGTTGTG - - - - - AATAGGCAAGC	-5 (×25)	homozygous	TGGATCCACAA - TAGGTCGAGATGTATGCTATG<br>TGGATCCACAAGTAGGTCGAGATGTATGCTATG	-1 (×5)<br>0 (×20)	heterozygous
	TGTGGGAACTGGTTGTGT - - - - AATAGGCAAGC	-4 (×25)	homozygous	TGGATCCACAAGTAGGTCGAGATGTATGCTATG	0 (×25)	WT

Table S6 Analysis of potential off-target sites among the Cas9/gRNA-induced *ptrcesa* mutants.

On/Off targets	gRNA name	Bases position of gRNAs																				PAM			Target gene or genomic position	Off-target Editing (Yes/No)
		1	2	3	4	5	6	7	8	9	10	11	12	13	14	15	16	17	18	19	20	21	22	23		
On	**gCesA4-1**	C	A	T	A	C	G	A	T	G	C	T	T	A	T	G	C	A	T	T	G	T	G	G	**PtrCesA4**	-
Off	gCesA4-1-off-1	G	T	G	A	G	G	A	G	G	C	T	T	A	T	G	C	A	T	T	G	T	G	G	Potri.019G000800	No
	gCesA4-1-off-2	T	G	A	G	G	G	A	T	G	C	T	T	A	T	G	C	T	T	T	G	T	G	G	Potri.006G052600	No
On	**gCesA4-2**	G	A	C	G	A	A	A	A	G	G	C	C	G	G	A	C	G	A	A	T	G	G	G	**PtrCesA4**	-
Off	-	NO off-target homologous sequences																				-			-	-
On	**gCesA4-3**	C	A	T	C	T	G	T	G	T	T	A	G	A	A	C	T	T	C	G	A	T	G	G	**PtrCesA4**	-
Off	gCesA4-3-off-1	A	A	G	A	T	G	T	T	T	G	A	G	A	A	C	T	T	C	G	A	T	G	G	Potri.001G144100	No
	gCesA4-3-off-2	T	T	A	A	G	C	T	G	T	T	A	G	A	A	C	T	T	C	G	A	C	G	G	Chr12 (+9333611)	No
On	**gCesA4-4**	C	T	C	C	T	C	C	A	A	G	G	A	C	C	T	T	G	A	G	G	A	G	G	**PtrCesA4**	-
Off	gCesA4-4-off-1	T	C	T	G	T	C	C	G	A	G	G	A	C	C	T	T	G	A	G	G	T	G	G	Potri.014G014400	No
	gCesA4-4-off-2	T	C	C	C	T	C	A	A	A	G	A	A	C	C	T	T	G	A	G	G	A	G	T	Chr19 (-6105951)	No
On	**gCesA7A/B-1**	A	C	G	C	A	A	A	G	A	A	A	G	A	G	G	G	A	G	G	G	T	G	G	**PtrCesA7A**	-
		A	C	G	A	A	A	A	G	A	A	A	G	A	G	G	G	A	G	G	G	T	G	G	**PtrCesA7B**	-
Off	gCesA7A/B-1-off-1	A	C	A	A	A	A	A	A	A	A	A	G	A	G	G	G	A	G	G	G	A	G	G	Potri.004G210200	No
	gCesA7A/B-1-off-2	A	G	A	G	A	A	A	G	A	A	A	G	A	G	G	G	A	G	G	G	A	G	G	Potri.001G314000	No
	gCesA7A/B-1-off-3	G	A	G	A	A	G	A	G	A	A	A	G	A	G	G	G	A	G	G	G	A	G	G	Potri.011G084300	No
	gCesA7A/B-1-off-4	A	A	A	A	A	G	A	G	A	A	A	G	A	G	G	G	A	G	G	G	A	G	G	Chr01(-21435525)	No
	gCesA7A/B-1-off-5	A	T	G	A	G	A	A	G	A	A	A	G	A	G	G	G	A	G	G	G	A	G	G	Potri.003G046500	No
	gCesA7A/B-1-off-6	A	C	G	C	A	A	G	G	A	G	A	G	A	G	G	G	A	G	G	G	T	G	A	Chr18 (-6513265)	No
On	**gCesA7A/B-2**	C	C	A	G	G	T	T	C	A	G	A	A	A	C	T	G	G	A	T	A	T	G	G	**PtrCesA7A**	-
		C	C	A	G	G	T	T	C	A	G	A	A	A	C	T	G	G	A	T	A	T	G	G	**PtrCesA7B**	-
Off	gCesA7A/B-2-off-1	T	G	A	T	G	T	T	C	T	T	A	A	A	C	T	G	G	A	T	A	T	G	G	Chr17 (+506091)	No
	gCesA7A/B-2-off-2	T	G	C	A	G	A	T	C	A	G	A	A	A	C	T	G	G	A	T	A	G	G	G	Potri.008G190100	No
On	**gCesA7A/B-3**	T	T	T	T	G	T	G	A	C	T	T	C	A	A	C	T	T	T	A	A	T	G	G	**PtrCesA7A**	-
		T	T	T	T	G	T	G	A	C	T	T	C	A	A	C	T	T	T	A	A	T	G	G	**PtrCesA7B**	-

Off	gCesA7A/B-3-off-1	T	G	T	T	C	T	G	A	C	T	T	C	A	A	C	T	T	T	A	A	A	G	G	Chr05(-12012362)	No
	gCesA7A/B-3-off-2	A	C	G	T	G	T	C	A	C	T	T	A	A	A	C	T	T	T	A	A	T	G	G	Scaffold_696	No
	gCesA7A/B-3-off-3	A	C	A	C	G	T	C	A	C	T	T	A	A	A	C	T	T	T	A	A	T	G	G	Chr12 (-5722266)	No
	gCesA7A/B-3-off-4	A	T	C	A	A	T	G	A	G	T	T	C	A	A	C	T	T	T	A	A	T	G	G	Chr12 (-5867397)	No
On	**gCesA7A/B-4**	G	C	C	G	C	T	G	G	A	C	T	C	G	A	G	G	A	A	G	G	A	G	G	**PtrCesA7A**	-
		G	C	T	G	C	C	G	G	A	C	T	C	G	A	G	G	A	A	G	G	A	G	G	**PtrCesA7B**	-
Off	gCesA7A/B-4-off-1	A	G	A	C	A	T	G	G	A	C	T	C	G	A	G	G	A	A	G	G	A	G	G	Potri.014G036100	No
On	**gCesA8A/B-1**	A	G	T	T	C	C	G	C	C	G	G	A	A	C	A	G	C	A	G	A	T	G	G	**PtrCesA8A**	-
		A	G	T	T	C	C	G	C	C	G	G	A	A	C	A	G	C	A	G	A	T	G	G	**PtrCesA8B**	-
Off	-	NO off-target homologous sequences																				-			-	-
On	**gCesA8A/B-2**	C	A	G	G	A	T	T	T	G	T	T	A	T	T	C	T	G	T	A	G	T	G	G	**PtrCesA8A**	-
		C	A	G	G	A	T	T	T	G	T	T	A	T	T	C	T	G	T	A	G	T	G	G	**PtrCesA8B**	-
Off	gCesA8A/B-2-off-1	C	A	G	G	A	T	T	T	G	T	T	A	T	T	C	G	G	T	A	G	T	G	C	Potri.001G266400	No
	gCesA8A/B-2-off-2	G	G	T	C	A	T	T	T	G	G	A	A	T	T	C	T	G	T	A	G	T	G	G	Potri.001G456800	No
On	**gCesA8A/B-3**	G	A	A	C	T	G	G	T	T	G	T	G	T	T	T	T	C	A	A	T	A	G	G	**PtrCesA8A**	-
		G	A	A	C	T	G	G	T	T	G	T	G	T	T	T	T	C	A	A	T	A	G	G	**PtrCesA8B**	-
Off	gCesA8A/B-3-off-1	G	A	A	C	T	G	G	T	T	G	T	C	T	A	T	T	C	A	T	T	A	G	G	Chr06 (+1623375)	No
	gCesA8A/B-3-off-2	A	A	C	C	T	G	G	T	T	G	T	G	T	T	T	G	C	A	T	T	A	G	G	Chr17(-10225639)	No
	gCesA8A/B-3-off-3	T	A	T	G	T	G	G	T	T	G	T	G	T	T	T	T	C	C	A	A	A	G	G	Potri.011G116200	No
	gCesA8A/B-3-off-4	G	A	G	C	T	G	G	T	T	G	T	G	T	T	T	T	A	A	A	A	A	G	A	Potri.004G147800	No
On	**gCesA8A/B-4**	C	A	T	A	C	A	T	C	T	C	G	A	C	C	T	A	C	T	T	G	T	G	G	**PtrCesA8A**	-
		C	A	T	A	C	A	T	C	T	C	G	A	C	C	T	A	C	T	T	G	T	G	G	**PtrCesA8B**	-
Off	-	NO off-target homologous sequences																				-			-	-

Nucleotides in green shading are the same with the designed gRNAs target sites and mismatched nucleotides are without shading. The PAM regions are highlighted in red shading. Potential off-target sites in protein coding gene regions are annotated with gene ID and the others located in noncoding regions are detailed with position information on chromosomes.

**gCesA4-1-off-1**		**Number of plasmids containing the cloned PCR products**
WT	GTGAGGAGGCTTATGCATTGTGG	(×10)
ptrcesa4-1#	GTGAGGAGGCTTATGCATTGTGG	(×15)
ptrcesa4-4#	GTGAGGAGGCTTATGCATTGTGG	(×15)

gCesA4-1-off-2
WT TGAGGGATGCTTATGCTTTGTGG (×10)
ptrcesa4-1# TGAGGGATGCTTATGCTTTGTGG (×15)
ptrcesa4-4# TGAGGGATGCTTATGCTTTGTGG (×15)

gCesA4-3-off-1
WT AAGATGTTTGAGAACTTCGATGG (×10)
ptrcesa4-6# AAGATGTTTGAGAACTTCGATGG (×15)

gCesA4-3-off-2
WT TTAAGCTGTTAGAACTTCGACGG (×10)
ptrcesa4-6# TTAAGCTGTTAGAACTTCGACGG (×15)

gCesA4-4-off-1
WT CCACCTCAAGGTCCTCGGACAGA (×10)
ptrcesa4-6# CCACCTCAAGGTCCTCGGACAGA (×15)

gCesA4-4-off-2
WT ACTCCTCAAGGTTCTTTGAGGGA (×10)
ptrcesa4-6# ACTCCTCAAGGTTCTTTGAGGGA (×15)

gCesA7A/B-1-off-1
WT ACAAAAAAAAAGAGGGAGGGAGG (×10)
ptrcesa7ab-3# ACAAAAAAAAAGAGGGAGGGAGG (×15)

gCesA7A/B-1-off-2
WT AGAGAAAGAAAGAGGGAGGGAGG (×10)
ptrcesa7ab-3# AGAGAAAGAAAGAGGGAGGGAGG (×15)

gCesA7A/B-1-off-3
WT GAGAAGAGAAAGAGGGAGGGAGG (×10)
ptrcesa7ab-3# GAGAAGAGAAAGAGGGAGGGAGG (×15)

gCesA7A/B-1-off-4
WT AAAAAGAGAAAGAGGGAGGGAGG (×10)
ptrcesa7ab-3# AAAAAGAGAAAGAGGGAGGGAGG (×15)

gCesA7A/B-1-off-5
WT ATGAGAAGAAAGAGGGAGGGAGG (×10)
ptrcesa7ab-3# ATGAGAAGAAAGAGGGAGGGAGG (×15)

gCesA7A/B-1-off-6
WT ACGCAAGGAGAGAGGGAGGGTGA (×6)

	ATGCAAGGAGAGAGGGAGGGTGA	(×4)	SNP
ptrcesa7ab-3#	ACGCAAGGAGAGAGGGAGGGTGA	(×7)	
	ATGCAAGGAGAGAGGGAGGGTGA	(×8)	

gCesA7A/B-2-off-1

WT	CCATATCCAGTTTAAGAACATCA	(×10)
ptrcesa7ab-3#	CCATATCCAGTTTAAGAACATCA	(×15)

gCesA7A/B-2-off-2

WT	CCCTATCCAGTTTCTGATCTGCA	(×10)
ptrcesa7ab-3#	CCCTATCCAGTTTCTGATCTGCA	(×15)

gCesA7A/B-3-off-1

WT	TGTTCTGACTTCAACTTTAAAGG	(×10)
ptrcesa7ab-7#	TGTTCTGACTTCAACTTTAAAGG	(×15)
ptrcesa7ab-13#	TGTTCTGACTTCAACTTTAAAGG	(×15)

gCesA7A/B-3-off-2

WT	ACGTGTCACTTAAACTTTAATGG	(×10)
ptrcesa7ab-7#	ACGTGTCACTTAAACTTTAATGG	(×15)
ptrcesa7ab-13#	ACGTGTCACTTAAACTTTAATGG	(×15)

gCesA7A/B-3-off-3

WT	ACACGTCACTTAAACTTTAATGG	(×10)
ptrcesa7ab-7#	ACACGTCACTTAAACTTTAATGG	(×15)
ptrcesa7ab-13#	ACACGTCACTTAAACTTTAATGG	(×15)

gCesA7A/B-3-off-4

WT	ATCAATGAGTTCAACTTTAATGG	(×10)
ptrcesa7ab-7#	ATCAATGAGTTCAACTTTAATGG	(×15)
ptrcesa7ab-13#	ATCAATGAGTTCAACTTTAATGG	(×15)

gCesA7A/B-4-off-1

WT	CCTCCTTCCTCGAGTCCATGTCT	(×10)
ptrcesa7ab-7#	CCTCCTTCCTCGAGTCCATGTCT	(×15)
ptrcesa7ab-13#	CCTCCTTCCTCGAGTCCATGTCT	(×15)

gCesA8A/B-2-off-1

WT	GCACTACCGAATAACAAATCCTG	(×10)
ptrcesa8ab-1#	GCACTACCGAATAACAAATCCTG	(×15)

gCesA8A/B-2-off-2

WT	CCACTACAGAATTCCAAATGACC	(×10)
ptrcesa8ab-1#	CCACTACAGAATTCCAAATGACC	(×15)

gCesA8A/B-3-off-1

WT	GAACTGGTTGTCTATTCATTAGG	(×5)	
	GAACTGGTTGTCTATTCATCAGG	(×5)	SNP
ptrcesa8ab-4#	GAACTGGTTGTCTATTCATTAGG	(×7)	
	GAACTGGTTGTCTATTCATCAGG	(×8)	
ptrcesa8ab-8#	GAACTGGTTGTCTATTCATTAGG	(×8)	
	GAACTGGTTGTCTATTCATCAGG	(×7)	

gCesA8A/B-3-off-2

WT	AACCTGGTTGTGTTTGCATTAGG	(×10)
ptrcesa8ab-4#	AACCTGGTTGTGTTTGCATTAGG	(×15)
ptrcesa8ab-8#	AACCTGGTTGTGTTTGCATTAGG	(×15)

gCesA8A/B-3-off-3

WT	TATGTGGTTGTGTTTTCCAAAGG	(×5)	
	TATGTGATTGTGTTTTCCAAACG	(×5)	SNP
ptrcesa8ab-4#	TATGTGGTTGTGTTTTCCAAAGG	(×7)	
	TATGTGATTGTGTTTTCCAAACG	(×8)	
ptrcesa8ab-8#	TATGTGGTTGTGTTTTCCAAAGG	(×6)	
	TATGTGATTGTGTTTTCCAAACG	(×9)	

gCesA8A/B-3-off-4

WT	GAGCTGGTTGTGTTTTAAAAAGA	(×10)
ptrcesa8ab-4#	GAGCTGGTTGTGTTTTAAAAAGA	(×15)
ptrcesa8ab-8#	GAGCTGGTTGTGTTTTAAAAAGA	(×15)

Table S7 Inheritance of the Cas9/gRNA-induced mutations in progenies of *ptrcesa4*, *7a/b* and *8a/b* mutants through asexual propagation methods. The progenies did change in the edited sites of *PtrCesA4*, *7A/B* or *8A/B*, in which was labeled with empty box and brilliant yellow color.

Line (#)		gCesA4-1<br>Target seq: CATACGATGCTTATGCATTGTGG	Mutation types<br>(Number of plasmids containing the cloned PCR products)		gCesA4-2<br>Target seq: CCCATTCGTCCGGCCTTTTCGTC	Mutation types<br>(Number of plasmids containing the cloned PCR products)	
ptrcesa4-2#		TCCAGCATACGATGCTTATG - TTGTGGCTTAT<br>TCCAGCATACGATGCTTATGCATTGTGGCTTAT	-1 (×17)<br>0 (×8)	heterozygous	TGCACCCCATT c CGTCCGGCCTTTTCGTCAGCAG<br>TGCACCCCATT t CGTCCGGCCTTTTCGTCAGCAG	+1 (×15)<br>+1 (×10)	biallelic
**Apical bud**	T0	TCCAGCATACGATGCTTATGC - TTGTGGCTTAT<br>TCCAGCATACGATGCTTATGCATTGTGGCTTAT	-1 (×7)<br>0 (×18)	heterozygous	TGCACCCCATT c CGTCCGGCCTTTTCGTCAGCAG<br>TGCACCCCATT t CGTCCGGCCTTTTCGTCAGCAG	+1 (×11)<br>+1 (×14)	biallelic
**Axillary bud**	T1-1	TCCAGCATACGATGCTTATGC - TTGTGGCTTAT<br>TCCAGCATACGATGCTTATGCATTGTGGCTTAT	-1 (×11)<br>0 (×14)	heterozygous	TGCACCCCATT c CGTCCGGCCTTTTCGTCAGCAG<br>TGCACCCCATT t CGTCCGGCCTTTTCGTCAGCAG	+1 (×11)<br>+1 (×14)	biallelic
	T1-2	TCCAGCATACGATGCTTATGC - TTGTGGCTTAT<br>TCCAGCATACGATGCTTATGCATTGTGGCTTAT	-1 (×13)<br>0 (×12)	heterozygous	TGCACCCCATT c CGTCCGGCCTTTTCGTCAGCAG<br>TGCACCCCATT t CGTCCGGCCTTTTCGTCAGCAG	+1 (×15)<br>+1 (×10)	biallelic
	T2-1	TCCAGCATACGATGCTTATGC - TTGTGGCTTAT<br>TCCAGCATACGATGCTTATGCATTGTGGCTTAT	-1 (×16)<br>0 (×9)	heterozygous	TGCACCCCATT c CGTCCGGCCTTTTCGTCAGCAG<br>TGCACCCCATT t CGTCCGGCCTTTTCGTCAGCAG	+1 (×12)<br>+1 (×13)	biallelic
	T2-2	TCCAGCATACGATGCTTATGC - TTGTGGCTTAT<br>TCCAGCATACGATGCTTATGCATTGTGGCTTAT	-1 (×17)<br>0 (×8)	heterozygous	TGCACCCCATT c CGTCCGGCCTTTTCGTCAGCAG<br>TGCACCCCATT t CGTCCGGCCTTTTCGTCAGCAG	+1 (×10)<br>+1 (×15)	biallelic
**Shoot regeneration**	T1-1	TCCAGCATACGATGCTTATGC - TTGTGGCTTAT<br>TCCAGCATACGATGCTTATGCATTGTGGCTTAT<br>TCCAGCATACGATGCTTATGC - TTGTGGCTTAT	-1 (×14)<br>0 (×9)<br>-1 (×2)	chimeric	TGCACCCCATT c CGTCCGGCCTTTTCGTCAGCAG<br>TGCACCCCATT t CGTCCGGCCTTTTCGTCAGCAG	+1 (×12)<br>+1 (×13)	biallelic
	T1-2	TCCAGCATACGATGCTTATGC - TTGTGGCTTAT<br>TCCAGCATACGATGCTTATGCATTGTGGCTTAT	-1 (×10)<br>0 (×15)	heterozygous	TGCACCCCATT c CGTCCGGCCTTTTCGTCAGCAG<br>TGCACCCCATT t - - - - - GCCTTTTCGTC AGCAG	+1 (×10)<br>+1-5 (×15)	biallelic
	T1-3	TCCAGCATACGATGCTTATGC - TTGTGGCTTAT<br>TCCAGCATACGATGCTTATGCATTGTGGCTTAT	-1 (×18)<br>0 (×7)	heterozygous	TGCACCCCATT c CGTCCGGCCTTTTCGTCAGCAG<br>TGCACCCCATT t CGTCCGGCCTTTTCGTCAGCAG	+1 (×13)<br>+1 (×12)	biallelic
**Line (#)**		**gCesA4-3**<br>Target seq: CATCTGTGTTAGAACTTCGATGG	**Mutation types**<br>(Number of plasmids containing the cloned PCR products)		**gCesA4-4**<br>Target seq: CCTCAAGGTCCTTGGAGGAG	**Mutation types**<br>(Number of plasmids containing the cloned PCR products)	
ptrcesa4-5#		AGCAACATCTGTGTTAGAA - - - CGATGGAGTG<br>AGCAACATCTGTGTTAGAAC - - CGATGGAGTG	-3 (×13)<br>-2 (×12)	biallelic	CAAGGCCTCCTCAAGGTCCTTGGAGGAGTAGAC	0 (×25)	WT
**Apical bud**	T0	AGCAACATCTGTGTTAGAA - - - CGATGGAGTG<br>AGCAACATCTGTGTTAGAAC - - CGATGGAGTG	-3 (×15)<br>-2 (×10)	biallelic	CAAGGCCTCCTCAAGGTCCTTGGAGGAGTAGAC	0 (×25)	WT
**Axillary bud**	T1-1	AGCAACATCTGTGTTAGAA - - - CGATGGAGTG<br>AGCAACATCTGTGTTAGAAC - - CGATGGAGTG	-3 (×11)<br>-2 (×14)	biallelic	CAAGGCCTCCTCAAGGTCCTTGGAGGAGTAGAC	0 (×25)	WT

	T1-2	AGCAACATCTGTGTTAGAA - - - CGATGGAGTG<br>AGCAACATCTGTGTTAGAAC - - CGATGGAGTG	-3 (×9)<br>-2 (×16)	biallelic	CAAGGCCTCCTCAAGGTCCTTGGAGGAGTAGAC	0 (×25)	WT
	T2-1	AGCAACATCTGTGTTAGAA - - - CGATGGAGTG<br>AGCAACATCTGTGTTAGAAC - - CGATGGAGTG	-3 (×10)<br>-2 (×15)	biallelic	CAAGGCCTCCTCAAGGTCCTTGGAGGAGTAGAC	0 (×25)	WT
	T2-2	AGCAACATCTGTGTTAGAA - - - CGATGGAGTG<br>AGCAACATCTGTGTTAGAAC - - CGATGGAGTG	-3 (×12)<br>-2 (×13)	biallelic	CAAGGCCTCCTCAAGGTCCTTGGAGGAGTAGAC	0 (×25)	WT
**Shoot regeneration**	T1-1	AGCAACATCTGTGTTAGAA - - - CGATGGAGTG<br>AGCAACATCTGTGTTAGAAC - - CGATGGAGTG	-3 (×12)<br>-2 (×13)	biallelic	CAAGGCCTCCTCAAGGTCCTTGGAGGAGTAGAC	0 (×25)	WT
	T1-2	AGCAACATCTGTGTTAGAA - - - CGATGGAGTG<br>AGCAACATCTGTGTTAGAAC - - CGATGGAGTG	-3 (×16)<br>-2 (×9)	biallelic	CAAGGCCTCCTCAAGGTCCTTGGAGGAGTAGAC	0 (×25)	WT
	T1-3	AGCAACATCTGTGTTAGAA - - - CGATGGAGTG<br>AGCAACATCTGTGTTAGAAC - - CGATGGAGTG	-3 (×17)<br>-2 (×8)	biallelic	CAAGGCCTCCTCAAGGTCCTTGGAGGAGTAGAC	0 (×25)	WT
**Line (#)**		**gCesA7A/B-3**<br>CesA7A, TTTTGTGACTTCAACTTTAATGG<br>CesA7B, TTTTGTGACTTCAACTTTAATGG	**Mutation types**<br>(Number of plasmids containing the cloned PCR products)		**gCesA7A/B-4**<br>CesA7A, CCTCCTTCCTCGAGTCCAGCGGC<br>CesA7B, CCTCCTTCCTCGAGTCCGGCAGC	**Mutation types**<br>(Number of plasmids containing the cloned PCR products)	
***ptrcesa7ab-5#*-7A**		GCAATTTTTGTGACTTCAACTTTAATGGAAGAA	0 (×25)	WT	GCGTACCTCCT - - - - CGAGTCCAGCGGCTCTGC<br>GCGTACC - - - TTCCTCGAGTCCAGCGGCTCTGC	-4 (×16)<br>-3 (×9)	biallelic
**-7B**		GCAATTTTTGTGACTTCAACTTTAATGGAAGAA	0 (×25)	WT	GTGTACCTCCT t TCCTCGAGTCCGGCAGCTCTGC<br>GTGTACCTCCT - CCTCGAGTCCGGCAGCTCTGC	+1 (×15)<br>-1 (×10)	biallelic
**Apical bud**	T0-7A	GCAATTTTTGTGACTTCAACTTTAATGGAAGAA	0 (×25)	WT	GCGTACCTCCT - - - - CGAGTCCAGCGGCTCTGC<br>GCGTACC - - - TTCCTCGAGTCCAGCGGCTCTGC	-4 (×9)<br>-3 (×16)	biallelic
	-7B	GCAATTTTTGTGACTTCAACTTTAATGGAAGAA	0 (×25)	WT	GTGTACCTCCT t TCCTCGAGTCCGGCAGCTCTGC<br>GTGTACCTCCT - CCTCGAGTCCGGCAGCTCTGC	+1 (×11)<br>-1 (×14)	biallelic
**Axillary bud**	T1-1-7A	GCAATTTTTGTGACTTCAACTTTAATGGAAGAA	0 (×25)	WT	GCGTACCTCCT - - - - CGAGTCCAGCGGCTCTGC<br>GCGTACC - - - TTCCTCGAGTCCAGCGGCTCTGC	-4 (×14)<br>-3 (×11)	biallelic
	-7B	GCAATTTTTGTGACTTCAACTTTAATGGAAGAA	0 (×25)	WT	GTGTACCTCCT t TCCTCGAGTCCGGCAGCTCTGC<br>GTGTACCTCCT - CCTCGAGTCCGGCAGCTCTGC	+1 (×13)<br>-1 (×12)	biallelic
	T1-2-7A	GCAATTTTTGTGACTTCAACTTTAATGGAAGAA	0 (×25)	WT	GCGTACCTCCT - - - - CGAGTCCAGCGGCTCTGC<br>GCGTACC - - - TTCCTCGAGTCCAGCGGCTCTGC	-4 (×13)<br>-3 (×12)	biallelic
	-7B	GCAATTTTTGTGACTTCAACTTTAATGGAAGAA	0 (×25)	WT	GTGTACCTCCT t TCCTCGAGTCCGGCAGCTCTGC<br>GTGTACCTCCT - CCTCGAGTCCGGCAGCTCTGC	+1 (×12)<br>-1 (×13)	biallelic
	T2-1-7A	GCAATTTTTGTGACTTCAACTTTAATGGAAGAA	0 (×25)	WT	GCGTACCTCCT - - - - CGAGTCCAGCGGCTCTGC<br>GCGTACC - - - TTCCTCGAGTCCAGCGGCTCTGC	-4 (×10)<br>-3 (×15)	biallelic
	-7B	GCAATTTTTGTGACTTCAACTTTAATGGAAGAA	0 (×25)	WT	GTGTACCTCCT t TCCTCGAGTCCGGCAGCTCTGC<br>GTGTACCTCCT - CCTCGAGTCCGGCAGCTCTGC	+1 (×14)<br>-1 (×11)	biallelic
	T2-2-7A	GCAATTTTTGTGACTTCAACTTTAATGGAAGAA	0 (×25)	WT	GCGTACCTCCT - - - - CGAGTCCAGCGGCTCTGC<br>GCGTACC - - - TTCCTCGAGTCCAGCGGCTCTGC	-4 (×16)<br>-3 (×9)	biallelic

	-7B	GCAATTTTTGTGACTTCAACTTTAATGGAAGAA	0 (×25)	WT	GTGTACCTCCT t TCCTCGAGTCCGGCAGCTCTGC<br>GTGTACCTCCT - CCTCGAGTCCGGCAGCTCTGC	+1 (×12)<br>-1 (×13)	biallelic
**Shoot regeneration**	T1-1-7A-7B	GCAATTTTTGTGACTTCAACTTTAATGGAAGAA	0 (×25)	WT	GCGTACCTCCT - - - - CGAGTCCAGCGGCTCTGC<br>GCGTACC - - - TTCCTCGAGTCCAGCGGCTCTGC	-4 (×10)<br>-3 (×15)	biallelic
		GCAATTTTTGTGACTTCAACTTTAATGGAAGAA	0 (×25)	WT	GTGTACCTCCT t TCCTCGAGTCCGGCAGCTCTGC<br>GTGTACCTCCT - CCTCGAGTCCGGCAGCTCTGC	+1 (×13)<br>-1 (×12)	biallelic
	T1-2-7A -7B	GCAATTTTTGTGACTTCAACTTTAATGGAAGAA	0 (×25)	WT	GCGTACCTCCT - - - - CGAGTCCAGCGGCTCTGC<br>GCGTACC - - - TTCCTCGAGTCCAGCGGCTCTGC	-4 (×14)<br>-3 (×11)	biallelic
		GCAATTTTTGTGACTTCAACTTTAATGGAAGAA	0 (×25)	WT	GTGTACCTCCT t TCCTCGAGTCCGGCAGCTCTGC<br>GTGTACCTCCT - CCTCGAGTCCGGCAGCTCTGC	+1 (×12)<br>-1 (×13)	biallelic
	T1-3-7A -7B	GCAATTTTTGTGACTTCAACTTTAATGGAAGAA	0 (×25)	WT	GCGTACCTCCT - - - - CGAGTCCAGCGGCTCTGC<br>GCGTACC - - - TTCCTCGAGTCCAGCGGCTCTGC	-4 (×14)<br>-3 (×11)	biallelic
		GCAATTTTTGTGACTTCAACTTTAATGGAAGAA	0 (×25)	WT	GTGTACCTCCT t TCCTCGAGTCCGGCAGCTCTGC<br>GTGTACCTCCT - CCTCGAGTCCGGCAGCTCTGC	+1 (×12)<br>-1 (×13)	biallelic
**Line#**		**gCesA7A/B-3**<br>CesA7A, TTTTGTGACTTCAACTTTAATGG<br>CesA7B, TTTTGTGACTTCAACTTTAATGG	**Mutation types**<br>(Number of plasmids containing the cloned PCR products)		**gCesA7A/B-4**<br>CesA7A, CCTCCTTCCTCGAGTCCAGCGGC<br>CesA7B, CCTCCTTCCTCGAGTCCGGCAGC	**Mutation types**<br>(Number of plasmids containing the cloned PCR products)	
***ptrcesa7ab-6#*-7A -7B**		GCAATTTTTGTGACTTCAACTTTAATGGAAGAA	0 (×25)	WT	GCGTACCTCCT - CCTCGAGTCCAGCGGCTCTG<br>GCGTACCTC - - - - - - - GAGTCCAGCGGCTCTG<br>GCGTACCTCCTTCCTCGAGTCCAGCGGCTCTG	-1 (×13)<br>-7 (×9)<br>0 (×3)	chimeric
		GCAATTTTTGTGACTTCAACTTTAATGGAAGAA	0 (×25)	WT	GTGTACCTCCTa TCCTCGAGTCCGGCAGCTCTG<br>GT - - - - - - - - - - CCTCGAGTCCGGCAGCTCTG<br>GTGTACCTCCTTCCTCGAGTCCGGCAGCTCTG	+1 (×9)<br>-10 (×12)<br>0 (×4)	chimeric
**Apical bud**	T0-7A -7B	GCAATTTTTGTGACTTCAACTTTAATGGAAGAA	0 (×25)	WT	GCGTACCTCCT - CCTCGAGTCCAGCGGCTCTG<br>GCGTACCTC - - - - - - - GAGTCCAGCGGCTCTG<br>GCGTACCTCCTTCCTCGAGTCCAGCGGCTCTG	-1 (×12)<br>-7 (×8)<br>0 (×5)	chimeric
		GCAATTTTTGTGACTTCAACTTTAATGGAAGAA	0 (×25)	WT	GTGTACCTCCTaTCCTCGAGTCCGGCAGCTCTG<br>GT - - - - - - - - - - CCTCGAGTCCGGCAGCTCTGC<br>GTGTACCTCCTTCCTCGAGTCCGGCAGCTCTG	+1 (×9)<br>-10 (×10)<br>0 (×6)	chimeric
**Axillary bud**	T1-1-7A -7B	GCAATTTTTGTGACTTCAACTTTAATGGAAGAA	0 (×25)	WT	GCGTACCTCCT- CCTCGAGTCCAGCGGCTCTGC<br>GCGTACCTC - - - - - - - GAGTCCAGCGGCTCTGC<br>GCGTACCTCCTTCCTCGAGTCCAGCGGCTCTGC	-1 (×13)<br>-7 (×8)<br>0 (×4)	chimeric
		GCAATTTTTGTGACTTCAACTTTAATGGAAGAA	0 (×25)	WT	GTGTACCTCCTa TCCTCGAGTCCGGCAGCTCTG<br>GT - - - - - - - - - - CCTCGAGTCCGGCAGCTCTGC<br>GTGTACCTCCTTCCTCGAGTCCGGCAGCTCTGC	+1 (×11)<br>-10 (×10)<br>0 (×4)	chimeric
	T1-2-	GCAATTTTTGTGACTTCAACTTTAATGGAAGAA	0 (×25)	WT	GCGTACCTCCT - CCTCGAGTCCAGCGGCTCTG	-1 (×10)	chimeric

	7A<br>-7B				GCGTACCTC - - - - - - - GAGTCCAGCGGCTCTGC<br>GCGTACCTCCTTCCTCGAGTCCAGCGGCTCTGC	-7 (×9)<br>0 (×6)	
		GCAATTTTGTGACTTCAACTTTAATGGAAGAA	0 (×25)	WT	GTGTACCTCCTaTCCTCGAGTCCGGCAGCTCTG<br>GT - - - - - - - - - - CCTCGAGTCCGGCAGCTCTGC<br>GTGTACCTCCTTCCTCGAGTCCGGCAGCTCTGC	+1 (×13)<br>-10 (×8)<br>0 (×4)	chimeric
	T2-1-7A<br>-7B	GCAATTTTGTGACTTCAACTTTAATGGAAGAA	0 (×25)	WT	GCGTACCTCCT- CCTCGAGTCCAGCGGCTCTGC<br>GCGTACCTC - - - - - - - GAGTCCAGCGGCTCTGC<br>GCGTACCTCCTTCCTCGAGTCCAGCGGCTCTGC	-1 (×10)<br>-7 (×10)<br>0 (×5)	chimeric
		GCAATTTTGTGACTTCAACTTTAATGGAAGAA	0 (×25)	WT	GTGTACCTCCTa TCCTCGAGTCCGGCAGCTCTG<br>GT - - - - - - - - - - CCTCGAGTCCGGCAGCTCTGC<br>GTGTACCTCCTTCCTCGAGTCCGGCAGCTCTGC	+1 (×11)<br>-10 (×9)<br>0 (×5)	chimeric
	T2-2-7A<br>-7B	GCAATTTTGTGACTTCAACTTTAATGGAAGAA	0 (×25)	WT	GCGTACCTCCT-CCTCGAGTCCAGCGGCTCTGC<br>GCGTACCTC - - - - - - - GAGTCCAGCGGCTCTGC<br>GCGTACCTCCTTCCTCGAGTCCAGCGGCTCTGC	-1 (×12)<br>-7 (×9)<br>0 (×4)	chimeric
		GCAATTTTGTGACTTCAACTTTAATGGAAGAA	0 (×25)	WT	GTGTACCTCCTa TCCTCGAGTCCGGCAGCTCTG<br>GT - - - - - - - - - - CCTCGAGTCCGGCAGCTCTGC<br>GTGTACCTCCTTCCTCGAGTCCGGCAGCTCTGC	+1 (×11)<br>-10 (×10)<br>0 (×4)	chimeric
**Shoot regeneration**	T1-1-7A<br>-7B	GCAATTTTGTGACTTCAACTTTAATGGAAGAA<br>GCAATTTTGTGACTTCAA - - - - - - - - - - - - ...	0 (×22)<br>-244 (×3)	heterozygous	GCGTACCTCCT-CCTCGAGTCCAGCGGCTCTGC<br>GCGTACCTC - - - - - - - GAGTCCAGCGGCTCTGC<br>... - - - - - - - - - - - - - - - - - - TTTACGGTTCAAT	-1 (×12)<br>-7 (×10)<br>-244 (×3)	chimeric
		GCAATTTTGTGACTTCAACTTTAATGGAAGAA	0 (×25)	WT	GTGTACCTCCTaTCCTCGAGTCCGGCAGCTCTGC<br>GT - - - - - - - - - - CCTCGAGTCCGGCAGCTCTGC	+1 (×11)<br>-10 (×14)	biallelic
	T1-2-7A<br>-7B	GCAATTTTGTGACTTCAACTTTAATGGAAGAA	0 (×25)	WT	GCGTACCTCCT-CCTCGAGTCCAGCGGCTCTGC<br>GCGTACCTC - - - - - - - GAGTCCAGCGGCTCTGC	-1 (×14)<br>-7 (×11)	biallelic
		GCAATTTTGTGACTTCAACTTTAATGGAAGAA	0 (×25)	WT	GTGTACCTCCTa TCCTCGAGTCCGGCAGCTCTGC<br>GT - - - - - - - - - - CCTCGAGTCCGGCAGCTCTGC	+1 (×12)<br>-10 (×13)	biallelic
	T1-3-7A<br>-7B	GCAATTTTGTGACTTCAACTTTAATGGAAGAA	0 (×25)	WT	GCGTACCTCCT- CCTCGAGTCCAGCGGCTCTGC<br>GCGTACCTC - - - - - - - GAGTCCAGCGGCTCTGC	-1 (×15)<br>-7 (×10)	biallelic
		GCAATTTTGTGACTTCAACTTTAATGGAAGAA	0 (×25)	WT	GTGTACCTCCTaTCCTCGAGTCCGGCAGCTCTGC<br>GT - - - - - - - - - - CCTCGAGTCCGGCAGCTCTGC	+1 (×11)<br>-10 (×14)	biallelic
**Line (#)**		**gCesA8A/B-1**<br>CesA8A-1, AGTTCCGCCGGAACAGCAGATGG<br>CesA8B-1, AGTTCCGCCGGAACAGCAGATGG	**Mutation types**<br>(Number of plasmids containing the cloned PCR products)		**gCesA8A/B-2**<br>CesA8A-2, CCACTACAGAATAACAAATCCTG<br>CesA8B-2, CCACTACAGAATAACAAATCCTG	**Mutation types**<br>(Number of plasmids containing the cloned PCR products)	
***ptrcesa8ab-3#*-8A<br>-8B**		GCTCAAGTTCCGCCGGAACA - - - GATGGAAGA<br>GCTCAAGTTCCGCCGGAA - - - - - GATGGAAGA	-3 (×18)<br>-5 (×7)	biallelic	TTCTTCCACTACAGAATAACAAATCCTGTTGAT	0 (×25)	WT
		GCTCAAGTTCCGCCGGA - - - - GAAGACTAAGA	-4 (×13)	biallelic	TTCTTCCACTACaAGAATAACAAATCCTGTCGA	+1 (×10)	chimeric

		GCTCAAGTTCCGCCG - - - - - - - - - ATGGAAGA	-9 (×12)		TTCTTCCACTAaCAGAATAACAAATCCTGTCGA<br>TTCTTCCACTA- AGAATAACAAATCCTGTCGAT<br>TTCTTCCACTACAGAATAACAAATCCTGTCGAT	+1 (×2)<br>-1 (×10)<br>0 (×3)	
**Apical bud**	T0-8A<br>-8B	GCTCAAGTTCCGCCGGAACA - - - GATGGAAGA<br>GCTCAAGTTCCGCCGGAA - - - - - GATGGAAGA	-3 (×19)<br>-5 (×6)	biallelic	TTCTTCCACTACAGAATAACAAATCCTGTTGAT	0 (×25)	WT
		GCTCAAGTTCCGCCGGA - - - - GAAGACTAAGA<br>GCTCAAGTTCCGCCG - - - - - - - - - ATGGAAGA	-4 (×14)<br>-9 (×11)	biallelic	TTCTTCCACTACaAGAATAACAAATCCTGTCGAT<br>TTCTTCCACTAaCAGAATAACAAATCCTGTCGAT<br>TTCTTCCACTA-AGAATAACAAATCCTGTCGAT<br>TTCTTCCACTACAGAATAACAAATCCTGTCGAT	+1 (×10)<br>+1 (×2)<br>-1 (×9)<br>0 (×4)	chimeric
**Axillary bud**	T1-1-<br>8A<br>-8B	GCTCAAGTTCCGCCGGAACA - - - GATGGAAGA<br>GCTCAAGTTCCGCCGGAA - - - - - GATGGAAGA	-3 (×18)<br>-5 (×7)	biallelic	TTCTTCCACTACAGAATAACAAATCCTGTTGAT	0 (×25)	WT
		GCTCAAGTTCCGCCGGA - - - - GAAGACTAAGA<br>GCTCAAGTTCCGCCG - - - - - - - - - ATGGAAGAT	-4 (×14)<br>-9 (×11)	biallelic	TTCTTCCACTACaAGAATAACAAATCCTGTCGAT<br>TTCTTCCACTAaCAGAATAACAAATCCTGTCGAT<br>TTCTTCCACTA-AGAATAACAAATCCTGTCGAT<br>TTCTTCCACTACAGAATAACAAATCCTGTCGAT	+1 (×10)<br>+1 (×4)<br>-1 (×9)<br>0 (×2)	chimeric
	T1-2-<br>8A<br>-8B	GCTCAAGTTCCGCCGGAACA - - - GATGGAAGA<br>GCTCAAGTTCCGCCGGAA - - - - - GATGGAAGA	-3 (×15)<br>-5 (×10)	biallelic	TTCTTCCACTACAGAATAACAAATCCTGTTGAT	0 (×24)	WT
		GCTCAAGTTCCGCCGGA - - - - GAAGACTAAGA<br>GCTCAAGTTCCGCCG - - - - - - - - - ATGGAAGA	-4 (×9)<br>-9 (×16)	biallelic	TTCTTCCACTACaAGAATAACAAATCCTGTCGAT<br>TTCTTCCACTAaCAGAATAACAAATCCTGTCGAT<br>TTCTTCCACTA-AGAATAACAAATCCTGTCGAT<br>TTCTTCCACTACAGAATAACAAATCCTGTCGAT	+1 (×10)<br>+1 (×2)<br>-1 (×11)<br>0 (×2)	chimeric
	T2-1-<br>8A<br>-8B	GCTCAAGTTCCGCCGGAACA - - - GATGGAAGA<br>GCTCAAGTTCCGCCGGAA - - - - - GATGGAAGA	-3 (×15)<br>-5 (×10)	biallelic	TTCTTCCACTACAGAATAACAAATCCTGTTGAT<br>TTCTTCCACTA - - - AATAACAAATCCTGTTGAT	0 (×20)<br>-3 (×5)	heterozygous
		GCTCAAGTTCCGCCGGA - - - - GAAGACTAAGA<br>GCTCAAGTTCCGCCG - - - - - - - - - ATGGAAGA	-4 (×15)<br>-9 (×10)	biallelic	TTCTTCCACTACaAGAATAACAAATCCTGTCGAT<br>TTCTTCCACTAaCAGAATAACAAATCCTGTCGAT<br>TTCTTCCACTA-AGAATAACAAATCCTGTCGAT<br>TTCTTCCACTACAGAATAACAAATCCTGTCGAT	+1 (×8)<br>+1 (×3)<br>-1 (×11)<br>0 (×3)	chimeric
	T2-2-<br>8A<br>-8B	GCTCAAGTTCCGCCGGAACA - - - GATGGAAGA<br>GCTCAAGTTCCGCCGGAA - - - - - GATGGAAGA	-3 (×14)<br>-5 (×11)	biallelic	TTCTTCCACTACAGAATAACAAATCCTGTTGAT	0 (×25)	WT
		GCTCAAGTTCCGCCGGA - - - - GAAGACTAAGA<br>GCTCAAGTTCCGCCG - - - - - - - - - ATGGAAGA	-4 (×10)<br>-9 (×15)	biallelic	TTCTTCCACTACaAGAATAACAAATCCTGTCGAT<br>TTCTTCCACTAaCAGAATAACAAATCCTGTCGAT<br>TTCTTCCACTA-AGAATAACAAATCCTGTCGAT<br>TTCTTCCACTACAGAATAACAAATCCTGTCGAT	+1 (×9)<br>+1 (×1)<br>-1 (×12)<br>0 (×3)	chimeric
**Shoot regeneration**	T1-1-<br>8A<br>-8B	GCTCAAGTTCCGCCGGAACA - - - GATGGAAGA	-3 (×25)	homozygous	TTCTTCCACTACAGAATAACAAATCCTGTTGAT<br>TTCTTCCAC - - - - - - - - AACAAATCCTGTTGAT	0 (×23)<br>-8 (×2)	heterozygous
		GCTCAAGTTCCGCCGGA - - - - GAAGACTAAGA<br>GCTCAAGTTCCGCCG - - - - - - - - - ATGGAAGA	-4 (×12)<br>-9 (×13)	biallelic	TTCTTCCACTAC aAGAATAACAAATCCTGTCGAT<br>TTCTTCACTA - AGAATAACAAATCCTGTCGAT	+1 (×12)<br>-1 (×13)	biallelic
	T1-2-	GCTCAAGTTCCGCCGGAACA - - - GATGGAAGA	-3 (×25)	homozygous	TTCTTCCACTACAGAATAACAAATCCTGTTGAT	0 (×25)	WT

	8A<br>-8B	GCTCAAGTTCCGCCGGA - - - - GAAGACTAAGA<br>GCTCAAGTTCCGCCG - - - - - - - - - ATGGAAGA	-4 (×13)<br>-9 (×12)	biallelic	TTCTTCCACTACaAGAATAACAAATCCTGTCGAT<br>TTCTTCCACTA- AGAATAACAAATCCTGTCGAT	+1 (×9)<br>-1 (×16)	biallelic
	T1-3-8A<br>-8B	GCTCAAGTTCCGCCGGAACA - - - GATGGAAGA<br>GCTCAAGTTCCGCCGGAA - - - - - GATGGAAGA	-3 (×20)<br>-5 (×5)	biallelic	TTCTTCCACTACAGAATAACAAATCCTGTTGAT<br>TTCTTCCACTA t CAGAATAACAAATCCTGTTGAT	0 (×22)<br>+1 (×2)	heterozygous
		GCTCAAGTTCCGCCGGA - - - - GAAGACTAAGA<br>GCTCAAGTTCCGCCG - - - - - - - - - ATGGAAGA	-4 (×12)<br>-9 (×13)	biallelic	TTCTTCCACTACaAGAATAACAAATCCTGTCGAT<br>TTCTTCCACTA- AGAATAACAAATCCTGTCGAT	+1 (×9)<br>-1 (×16)	biallelic

**Line (#)**		**gCesA8A/B-3**<br>CesA8A-3, GAACTGGTTGTGTTTTCAATAGG<br>CesA8B-3, GAACTGGTTGTGTTTTCAATAGG	**Mutation types**<br>(Number of plasmids containing the cloned PCR products)		**gCesA8A/B-4**<br>CesA8A-4, CCACAAGTAGGTCGAGATGTATG<br>CesA8B-4, CCACAAGTAGGTCGAGATGTATG	**Mutation types**<br>(Number of plasmids containing the cloned PCR products)	
***ptrcesa8ab-6#*-8A<br>-8B**		TGTGGGAACTGGTTGTGTTTTC t AATAGGCAAG<br>TGTGGGAACTGGT - …	+1 (×18)<br>-31 (×7)	biallelic	TGGATCCACAAaGTAGGTCGAGATGTATGCTATG<br>TGGATCCACAAGTAGGTCGAGATGTATGCTATG	+1 (×22)<br>0 (×3)	heterozygous
		TGTGGGAACTGGTTGTGTTTTCaAATAGGCAAG<br>TGTGGGAACTGGTTGTGTTTTCaa ATAGGCAAG<br>TGTGGGAACTGGTTGTGTA - - - AATAGGCAAG	+1 (×15)<br>+2 (×2)<br>-3 (×8)	chimeric	TGGATCCACAAGTAGGTCGAGATGTATGCTATG	0 (×25)	WT
**Apical bud**	T0-8A<br>-8B	TGTGGGAACTGGTTGTGTTTTC t AATAGGCAAG<br>TGTGGGAACTGGT - …	+1 (×14)<br>-31 (×11)	biallelic	TGGATCCACAAaGTAGGTCGAGATGTATGCTATG<br>TGGATCCACAAGTAGGTCGAGATGTATGCTATG	+1 (×17)<br>0 (×8)	heterozygous
		TGTGGGAACTGGTTGTGTTTTCaAATAGGCAAG<br>TGTGGGAACTGGTTGTGTTTTCaa ATAGGCAAG<br>TGTGGGAACTGGTTGTGTA - - - AATAGGCAAG	+1 (×13)<br>+2 (×2)<br>-3 (×10)	chimeric	TGGATCCACAAGTAGGTCGAGATGTATGCTATG	0 (×25)	WT
**Axillary bud**	T1-1-8A<br>-8B	TGTGGGAACTGGTTGTGTTTTCtAATAGGCAAG<br>TGTGGGAACTGGT - …	+1 (×12)<br>-31 (×13)	biallelic	TGGATCCACAAaGTAGGTCGAGATGTATGCTATG<br>TGGATCCACAAGTAGGTCGAGATGTATGCTATG	+1 (×13)<br>0 (×12)	heterozygous
		TGTGGGAACTGGTTGTGTTTTCaAATAGGCAAG<br>TGTGGGAACTGGTTGTGTTTTCaaATAGGCAAG<br>TGTGGGAACTGGTTGTGTA - - - AATAGGCAAG	+1 (×17)<br>+2 (×1)<br>-3 (×7)	chimeric	TGGATCCACAAGTAGGTCGAGATGTATGCTATG	0 (×25)	WT
	T1-2-8A<br>-8B	TGTGGGAACTGGTTGTGTTTTC t AATAGGCAAG<br>TGTGGGAACTGGT - …	+1 (×12)<br>-31 (×13)	biallelic	TGGATCCACAAaGTAGGTCGAGATGTATGCTATG<br>TGGATCCACAAGTAGGTCGAGATGTATGCTATG	+1 (×15)<br>0 (×10)	heterozygous
		TGTGGGAACTGGTTGTGTTTTCaAATAGGCAAG<br>TGTGGGAACTGGTTGTGTTTTCaa ATAGGCAAG<br>TGTGGGAACTGGTTGTGTA - - - AATAGGCAAG	+1 (×18)<br>+2 (×1)<br>-3 (×6)	chimeric	TGGATCCACAAGTAGGTCGAGATGTATGCTATG	0 (×25)	WT
	T2-1-8A<br>-8B	TGTGGGAACTGGTTGTGTTTTC t AATAGGCAAG<br>TGTGGGAACTGGT - …	+1 (×10)<br>-31 (×15)	biallelic	TGGATCCACAAaGTAGGTCGAGATGTATGCTATG<br>TGGATCCACAAGTAGGTCGAGATGTATGCTATG	+1 (×16)<br>0 (×9)	heterozygous
		TGTGGGAACTGGTTGTGTTTTCaAATAGGCAAG<br>TGTGGGAACTGGTTGTGTTTTCaa ATAGGCAAG<br>TGTGGGAACTGGTTGTGTA - - - AATAGGCAAG	+1 (×20)<br>+2 (×1)<br>-3 (×5)	chimeric	TGGATCCACAAGTAGGTCGAGATGTATGCTATG	0 (×25)	WT
	T2-1-8A<br>-8B	TGTGGGAACTGGTTGTGTTTTC t AATAGGCAAG<br>TGTGGGAACTGGT - …	+1 (×15)<br>-31 (×10)	biallelic	TGGATCCACAA a<br>GTAGGTCGAGATGTATGCTATG<br>TGGATCCACAAGTAGGTCGAGATGTATGCTATG	+1 (×16)<br>0 (×9)	heterozygous

		TGTGGGAACTGGTTGTGTTTTCa AATAGGCAAG<br>TGTGGGAACTGGTTGTGTTTTCaaAATAGGCAA<br>G<br>TGTGGGAACTGGTTGTGTA - - - AATAGGCAAG	+1 (×17)<br>+2 (×1)<br>-3 (×7)	chimeric	TGGATCCACAAGTAGGTCGAGATGTATGCTATG	0 (×25)	WT
**Shoot regeneration**	T1-1-8A-8B	TGTGGGAACTGGTTGTGTTTTCt AATAGGCAAG<br>TGTGGGAACTGGT -…	+1 (×11)<br>-31 (×14)	biallelic	TGGATCCACAA a GTAGGTCGAGATGTATGCTATG<br>TGGATCCACAAGTAGGTCGAGATGTATGCTATG	+1 (×15)<br>0 (×10)	heterozygous
		TGTGGGAACTGGTTGTGTTTTCa AATAGGCAAG	+1 (×25)	homozygous	TGGATCCACAAGTAGGTCGAGATGTATGCTATG<br>TGGATCCACAA c<br>GTAGGTCGAGATGTATGCTATG	0 (×23)<br>+1 (×2)	heterozygous
	T1-2-8A-8B	TGTGGGAACTGGTTGTGTTTTC t AATAGGCAAG<br>TGTGGGAACTGGT - - - - - - - - - - - - - - - - - - - …	+1 (×20)<br>-31 (×5)	biallelic	TGGATCCACAA a GTAGGTCGAGATGTATGCTATG<br>TGGATCCACAAGTAGGTCGAGATGTATGCTATG	+1 (×16)<br>0 (×9)	heterozygous
		TGTGGGAACTGGTTGTGTA - - - AATAGGCAAGC<br>… - AATAGGCAAGC	-3 (×3)<br>-689(×22)	biallelic	TGGATCCACAAGTAGGTCGAGATGTATGCTATG<br>TGGATCCACAAA - …	0 (×3)<br>-689 (×22)	heterozygous
	T1-3-8A-8B	TGTGGGAACTGGTTGTGTTTTC t AATAGGCAAG<br>TGTGGGAACTGGT - - - - - - - - - - - - - - - - - - - …	+1 (×9)<br>-31 (×16)	biallelic	TGGATCCACAA a GTAGGTCGAGATGTATGCTATG<br>TGGATCCACAAGTAGGTCGAGATGTATGCTATG	+1 (×9)<br>0 (×16)	heterozygous
		TGTGGGAACTGGTTGTGTTTTCa AATAGGCAAG<br>… -AATAGGCAAGC	+1 (×2)<br>-689(×23)	biallelic	TGGATCCACAAGTAGGTCGAGATGTATGCTATG<br>TGGATCCACAAA - …	0 (×2)<br>-689 (×23)	heterozygous

Table S8 Inheritance of the Cas9/gRNA-induced mutations in the progeny of the *ptrcesa* lines.

ptrcesa **line#**	**Genotype**	**Apical bud**	**Axillary bud**				**Shoot regeneration**		
		**T0**	**T1-1**	**T1-2**	**T2-1**	**T2-2**	**T1-1**	**T1-2**	**T1-3**
ptrcesa4-2#	heterozygous/biallelic	N/N	N/N	N/N	N/N	N/N	Y/ N	N/Y	N/N
ptrcesa4-5#	biallelic/WT	N/N	N/N	N/N	N/N	N/N	N/N	N/N	N/N
***ptrcesa7ab-5#*-7A**	WT/biallelic	N/N	N/N	N/N	N/N	N/N	N/N	N/N	N/N
***-5#*-7B**	WT/biallelic	N/N	N/N	N/N	N/N	N/N	N/N	N/N	N/N
***ptrcesa7ab-6#*-7A**	WT/chimeric	N/N	N/N	N/N	N/N	N/N	Y/Y	N/Y	N/Y
***-6#*-7B**	WT/chimeric	N/N	N/N	N/N	N/N	N/N	N/Y	N/Y	N/Y
***ptrcesa8ab-3#*-8A**	biallelic/WT	N/N	N/ N	N/ N	N /Y	N/N	Y/Y	Y/N	N/Y
***-3#*-8B**	biallelic/chimeric	N/ N	N/ N	N/ N	N/ N	N/ N	N/Y	N/Y	N/Y
***ptrcesa8ab-6#*-8A**	biallelic/heterozygous	N/N	N/ N	N/N	N/ N	N/N	N/N	N/N	N/N
***-6#*-8B**	chimeric/WT	N /N	N /N	N /N	N /N	N/N	Y/Y	Y/Y	Y/Y

Biallelic, two alleles of the gene were edited and the different mutations were induced.
Chimeric, more than three different allelic mutations in a target editing site of the gene.
